# Interplay of four types of RNA modification writers revealed distinct tumor microenvironment and biological characteristics in pancreatic cancer

**DOI:** 10.3389/fimmu.2022.1031184

**Published:** 2022-12-19

**Authors:** Wenzhe Gao, Dongjie Chen, Jixing Liu, Longjun Zang, Tijun Xiao, Xianlin Zhang, Zheng Li, Hongwei Zhu, Xiao Yu

**Affiliations:** ^1^ Department of Hepatopancreatobiliary Surgery, Third Xiangya Hospital, Central South University, Changsha, Hunan, China; ^2^ Department of General Surgery, Pancreatic Disease Center, Ruijin Hospital, Shanghai Jiao Tong University School of Medicine, Shanghai, China; ^3^ Shanghai Key Laboratory of Translational Research for Pancreatic Neoplasms, Research Institute of Pancreatic Diseases, Shanghai Jiaotong University School of Medicine, Shanghai, China; ^4^ State Key Laboratory of Oncogenes and Related Genes, Institute of Translational Medicine, Shanghai Jiaotong University, Shanghai, China; ^5^ Department of Nephrology, Institute of Nephrology, 2nd Affiliated Hospital of Hainan Medical University, Haikou, Hainan, China; ^6^ Department of General Surgery, Shaoyang University Affiliated Second Hospital, Shaoyang University, Shaoyang, Hunan, China; ^7^ Department of General Surgery, Affiliated Renhe Hospital of China Three Gorges University, Yichang, Hubei, China

**Keywords:** RNA modification writers, tumor microenvironment, pancreatic cancer, molecular classification, immunotherapy

## Abstract

**Background:**

Pancreatic cancer (PC) is one of the most lethal malignancies and carries a dismal mortality and morbidity. Four types of RNA modification (namely m6A, m1A, APA and A-to-I) could be catalyzed by distinct enzymatic compounds (“writers”), mediating numerous epigenetic events in carcinogenesis and immunomodulation. We aim to investigate the interplay mechanism of these writers in immunogenomic features and molecular biological characteristics in PC.

**Methods:**

We first accessed the specific expression pattern and transcriptional variation of 26 RNA modification writers in The Cancer Genome Atlas (TCGA) dataset. Unsupervised consensus clustering was performed to divide patients into two RNA modification clusters. Then, based on the differentially expressed genes (DEGs) among two clusters, RNA modification score (WM_Score) model was established to determine RNA modification-based subtypes and was validated in International Cancer Genome Consortium (ICGC) dataset. What’s more, we manifested the unique status of WM_Score in transcriptional and post-transcriptional regulation, molecular biological characteristics, targeted therapies and immunogenomic patterns.

**Results:**

We documented the tight-knit correlations between transcriptional expression and variation of RNA modification writers. We classified patients into two distinct RNA modification patterns (WM_Score_high and _low), The WM_Score_high subgroup was correlated with worse prognosis, Th2/Th17 cell polarization and oncogenic pathways (e.g. EMT, TGF-β, and mTORC1 signaling pathways), whereas the WM_Score_low subgroup associated with favorable survival rate and Th1 cell trend. WM_Score model also proved robust predictive power in interpreting transcriptional and post-transcriptional events. Additionally, the potential targeted compounds with related pathways for the WM_Score model were further identified.

**Conclusions:**

Our research unfolds a novel horizon on the interplay network of four RNA modifications in PC. This WM_Score model demonstrated powerful predictive capacity in epigenetic, immunological and biological landscape, providing a theoretical basis for future clinical judgments of PC.

## 1 Introduction

Pancreatic cancer (PC) is one of the most devastating cancers, with 5-year survival rates of<5% among solid cancers (1). Existing evidence reported that the progression of PC results from multiple activated pathways and crosstalk events in epigenetic levels (2). Epigenetics deals with changes in gene expression resulting directly from mutations of DNA sequences, leading to the formation of inherited traits both intra-generationally and inter-generationally (3). It was also found that RNA modification as a reversible epigenetic mechanism, plays a pivotal role in almost all vital bioprocesses, including tumorigenesis (4).

RNA modification, a molecular process, can make changes to specific nucleotide sequences such as A, C, G, and U residues (5). With the rapid evolution of transcriptome-wide profiling, more than 170 different types of RNA modifications were found including N6-methyladenosine (m6A), 5-methylcytosine (m5C), pseudouridine (Ψ), and N1-methyladenosine (m1A) (6–9). Since the complexity of the epitranscriptome landscape, plenty of studies suggested that there might be some kinds of underlying interactions among those modifications. For example, m6A modification deficiency was confirmed to generate the inflated level of A-to-I editing *via* positive regulation of ADAR with m6A-depleted transcripts (10), while m1A and m5C may also play a relevant part in regulating A-to-I editing (11). Hence, we concentrated on four common adenine-related RNA modifications (including m6A, m1A, APA and A-to-I) to explore the interplay of their promoters termed as “writers”.

m6A refers to the methylation at position N6 of adenosine, which is the most prevalent modification throughout the mammalian RNA transcriptome, regulating the different stages of RNA metabolism including RNA-protein interaction and RNA stability (12). This modification was catalyzed by multicomponent methyltransferases such as METTL3, METTL14, METTL16, WTAP, VIRMA and RBM15 (13). Extensive studies have acknowledged the vital functions of the m6A in numerous physiological processes, especially in cancer progression (14, 15). m1A is developed by adding a methyl group to the nitrogen-1 position of adenosine. Under physiological circumstances, m1A carries a positive charge which influences RNA-protein interaction and RNA structure (9). m1A writers act as methyltransferase complexes contain TRMT6/61A, TRMT61B, and TRMT10C (16). It has been reported that m1A modification comprehensively engaged in the initiation and development of many diseases (17, 18). APA, namely alternative polyadenylation, is an RNA-processing mechanism that generates distinct 3’ ends in transcripts made by RNA polymerase II, thus significantly broadening the diversity of mRNAs and proteins (19). Several 3’-end-processing factors (e.g., CPSF, CSTF and CFIm complex) were proved to regulate the ploy (A) selection and alteration (20). Deficiency in 3’UTR may contribute to the onset of various cancers which in turn accelerate the development of target therapy, for instance, the shortening of the KHDRBS1 mRNA 3’UTR can mediate the upregulation of KHDRBS1 and promote the progression of gastric cancer (21). A-to-I is one of the RNA-editing mechanisms which is mediated by ADAR family members (ADAR, ADARB1 and ADARB2) (22). These modifications can be directly recognized as adenosine-to-guanosine mismatches in transcriptome (23), then make a positive or negative contribution to tumor progression by modifying oncogenes (24). For further understanding the meaning of epigenetic modifications, the exploration of interplay in these four types of RNA modifications is an urgent need.

It is worth noting that research on the role of the above common types of RNA epigenetic modification is still incomplete, let alone the crosstalk between these diverse types. In 2020, Swati V and colleagues (25) have reported the widespread, recurrent and functionally relevant 3’ UTR APA events in PC patients by profiling data and have experimentally validated the effects of several APA events, including CSNK1A1, FLNA and PAF1 on miRNA regulation, protein expression as well as tumor growth. Similarly, phenomenological research on m1A and A-to-I was also archived in PC. ALKBH3 as a m1A demethylase and ADAR1 as an A-to-I editor were found highly expressed in PC compared with normal pancreas (26). However, there is a lack of further studies to explore the mechanism and tumor-promoting functionality of these anomalies in the regulation of specific oncogenes/antioncogenes. Moreover, these published studies have indicated a distinct RNA modification pattern in PC compared with other malignancies. Taking m6A, the most intensively studied type in RNA epigenetics as an example. Several studies have suggested m6A “writer” METTL14, instead of METTL3 which acts as central methylase most of the time, is the key regulator leading to the elevated m6A levels in PC samples. Wang M et al (27) published a m6A-dependent METTL14/PERP/TP53 axis promoting the growth and metastasis of PC. Chen S et al (28) reported that CLK1-SRSF5 axis regulated METTL14 exon10 skipping enhanced the transcriptomic m6A modification level and promoted PC metastasis. The above studies show that the role of RNA epigenetic modification in PC still deserves further exploration.

Given the immunological “cold” characteristics of PC, immunotherapy is facing tremendous challenges and imperatively needs to strive for a breakthrough (29). To facilitating the sensitivity of immunotherapy, investigating the tumor immune microenvironment (TIME) and recognizing the potential resistant mechanism for individual patients should be emphasized (30). In recent years, RNA modifications and their writers were deemed as a novel regulator of the tumor immune system. For example, METTL3- or METTL4-deficient tumors enhanced the infiltration of CD8+T cells and increased the potency of anti-PD1 therapy in colorectal cancer (31). METTL14 was also determined as a target for enhancing immunotherapy in rectal cancer (32). In addition, the shortening 3’UTR served as a significant part in the immunotherapy and targeted therapy of clear cell renal carcinoma (33). However, several studies reveal the distinct TIME pattern in PC *via* combing these four types of RNA modification together. Hence, perceiving the regulatory mechanism of mixed RNA modification writers in TIME cells infiltration help unlock broad prospects for the development of immunotherapy.

In our study, transcriptional variation of PC patients from The Cancer Genome Atlas (TCGA) and International Cancer Genome Consortium (ICGC) was included to access the specific RNA modification patterns. This pattern is either correlated with infiltration of immune cells, or enriched in epithelial-mesenchymal transition (EMT), TGF-β and multiple carcinogenic signaling pathways. Then, we established the writers of the RNA modification score (WM_Score) model based on the differentially expressed genes (DEGs) to evaluate the predictive capacity in the distinct pattern. At last, we manifested the unique status of WM_Score in transcriptional and post-transcriptional regulation, molecular biological characteristics, targeted therapies and immunogenomic patterns. The flowchart of this study was shown in **Figure S1**.

## 2 Materials and methods

### 2.1 Datasets obtaining and processing

The workflow of this study was shown in **Figure S1**. Public transcriptional profiling datasets from patients’, including TCGA_PAAD dataset, GSE62452 GSE57495 and GSE28735 dataset from GEO, ICGC_AU_PAAD dataset were included. For TCGA_PAAD dataset, somatic mutation, copy number variation, RNA expression in FPKM format data, as well as complete clinical information was obtained from UCSC Xena (https://xenabrowser.net/datapages/). RNA-seq data in FPKM format was then transformed into TPM by R. GEO datasets were downloaded from https://www.ncbi.nlm.nih.gov/geo/ in raw data format and further disposed using R package affy; R package sva were then utilized to combine different sourced GEO datasets. ICGC_AU_PAAD dataset were downloaded from https://dcc.icgc.org/, somatic mutation data, FPKM RNA-seq data and clinical information were included in our study. All the above data was analyzed in R (version 4.1.1) and Bioconductor packages for data cleaning and gene signature annotation. The detailed information for these datasets were listed in **Supplementary Table 1**.

### 2.2 Mutation and copy number variation (CNV) analysis

For somatic mutational analysis, SNP6 array data was first obtained from TCGA and ICGC datasets. Then, non-silent mutation types were excluded and the remained data was imported into R package GenVisR for visualization. For CNV analysis, GenePattern platform (https://www.genepattern.org/) was utilized according to its GISTIC2.0 module. The parameter settings were as followed: confidence level 0.99, q-value threshold 0.25, join segment size 4, gene gistic YES, remove X NO, cap value 1.5, max sample segs 2000, gene collapse method exteme.

### 2.3 Unsupervised consensus clustering

After expression matrix was standardized using sweep() function in R, package ConsensusClusterPlus was applied for gene expression clustering. The parameters in this study were set as: maxK=4, reps=100, pItem=0.8, pFeature=1, title=title, clusterAlg=“pam”, distance=“spearman”. Consensus CDF value and delta area of CDF curve were used as evaluation criteria for every single clustering.

### 2.4 Construction of WM_score model

#### 2.4.1 Identification of Writer_clusters-related differentially expressed genes (DEGs)

Based on the two Writer_clusters identified by consensus clustering, we performed differential expression (DE) analysis using R package LIMMA. In brief, we first excluded genes in the dataset that were expressed as 0 in more than 20% of the samples; then functions in LIMMA package including makeContrasts(), lmFit() and eBayes() were applied in turn to build the linear model and extrack results of DE analysis. The standards defined as DEGs were adjusted P value< 0.05 and absolute value of log2FoldChange ≥1.

#### 2.4.2 Model construction by LASSO-cox method

The above DEGs were first introduced into a uni-variate cox regression along with survival information of samples by R package ezcox to first identified writer_clusters-related, differentially expressed prognostic genes, which were defined as candidates for WM_score model. To further narrow down the number of genes included in the final model, LASSO-cox algorithm was then applied. We first randomly divided TCGA_PAAD cohort into train set and internal test set using R package caret. Then, glmnet package was applied for model construction. The finally included genes and their corresponding LASSO-cox coefficients were extracted to calculate WM_score for each sample following the following formula:


$$Risk\;Score=\;\sum_{i=1}^{n}Coef_{i}*x_{i}$$

where Coefi meant the LASSO-cox coefficients for each gene, xi was the TPM value of each gene.

#### 2.4.3 Validation of WM_score model

To further detect the efficacy of WM_score in predicting prognosis of PDAC patients, we performed internal and external validation in both TCGA_PAAD, GSE57495 and ICGC_AU_PAAD datasets. Time-dependent ROC curve and Area Under the Curve (AUC) implemented by R package survivalROC and plotROC was applied to evaluate the predicting ability of WM_score model.

### 2.5 Gene set variation analysis (GSVA) and GSVA based EMT-score

To explore diverse enrichment status in gene function for different clusters and/or subgroups, GSVA was applied by R package GSVA and GSEAbase. Two gene sets were conducted for functional annotation from MsigDB (http://www.gsea-msigdb.org/gsea/msigdb/), which were c2.cp.kegg.v7.4.symbols and h.all.v7.4.symbols, respectively. LIMMA package was then utilized to distinguish the enrichment differences between different subgroups.

### 2.6 Correlation between WM_score and multiple molecular subtypes of PDAC

Based on the literature published by Moffitt, Collisson and Bailey et al (34–36), gene sets for each molecular subtype were first extracted, resulting in 50, 62 and 1939 genes included for Moffitt, Collisson and Bailey subtyping, respectively; while genes for Bailey subtyping were further divided into 240 ADEX, 1,061 squamous, 268 progenitor and 370 immunogenic genes. Then, consistent with the consensus clustering method mentioned above, we manually performed clustering in TCGA_PAAD dataset and assigned subtypes by overlapping the consensus clustering results and expression patterns of the subtyping genes included.

### 2.7 Analysis between WM_Score-high and -low groups for transcriptional and post-transcriptional events

#### 2.7.1 Correlation between WM_score and miRNA targeting

The expression matrix of microRNAs (miRNAs) from TCGA_PAAD dataset was downloaded from UCSC xena as mentioned above. DE analysis for miRNAs was also performed by Limma-Voom method and potential targeting relationship between DE miRNAs and WM_score DEGs was predicted by Diana tools (http://diana.imis.athena-innovation.gr/DianaTools/index.php). Finally, Sankey diagram was applied to depict this targeting relationship by R package ggalluvial.

#### 2.7.2 Association between WM_score and APA events

APA events for TCGA_PAAD cohort were accessed from The Cancer 3′ UTR Atlas (TC3A, http://tc3a.org) and original data were downloaded from (https://www.synapse.org/#!Synapse:syn24982198/files/) (37). Percentage of Distal polyA site Usage Index (PDUI) was evaluated by DaPars2 algorithm to identify the alternative proximal polyA site. Thus, PDUI values were regarded as quantitative indicators to identify 3’UTR lengthening (positive index) or shortening (negative index). DE analysis for PDUI was also performed based on Limma package. FDR<0.05 and PDUI difference > 0.1 were considered as statistically significant.

#### 2.7.3 Association between WM_score and m6A/m1A modification

To identify the m6A or m1A dependent regulation between WM_score related DEGs and all of the m6A/m1A regulators, RMBase online tool was applied and the original data from this database was downloaded from (https://rna.sysu.edu.cn/rmbase/download.php). The regulatory relationships included in this database as high/extremely high reliable were summarized in this study.

### 2.8 Compound resistance and sensitivity analysis

Genomics of Drug Sensitivity in Cancer (GDSC, http://www.cancerrxgene.org/downloads) database, which contained drug sensitivity data (IC50) of 1,000 cell lines, were accessed to get drug sensitivity and resistance information for pancreatic cancer cell lines. Spearman correlation analysis was performed to calculate the correlation between drug sensitivity and WM_Score, the absolute value of correlation coefficient > 0.2 and FDR< 0.1 were regarded as significant.

### 2.9 Analysis for immune cell infiltration and immune signatures

To assess the overall immune infiltration and stromal purity of tumor samples, we first applied ESTIMATE algorithm in R followed standard analysis process. For tumor immune cell infiltration analysis, we adopted two algorithms: CIBERSORT and ssGSEA. We downloaded archives that contained defining gene signatures for every immune cell type from the original manuscripts. For T cell differential evaluation, we applied GSVA algorithm to evaluate based on the MsigDB and Pathcards (https://pathcards.genecards.org/) gene sets, which were BIOCARTA_IL12_PATHWAY and BIOCARTA_IL4_PATHWAY from MsigDB for Th1/Th2 development and Th17 Differentiation pathway from Pathcards. We also referred to the literature published by Eric R L et al. (38) to determine the characteristic markers of various types of immune cells including Treg cells.

### 2.10 Statistical analysis

All statistical analysis were performed in R (version 4.1.1). The comparison of count data was tested by Fisher’s test and Chi-square test. For the measurement data that conformed to the normal distribution, Student-t test was applied; besides, Wilcox test was applied for non-normal distribution data between independent subgroups. Spearman analysis was applied to estimate the correlations between two variables that are note linearly related. K-M test was utilized to validate the fraction of PC patients living for a certain survival time and log-rank test was conducted to compare the significance of difference. R package survival and survival miner were used for depicting Kaplan-Meier survival curve. A two-tailed p-value of less than 0.05 was deemed to be statistically significant unless specifically stated.

## 3 Results

### 3.1 Transcriptional variation of four types of RNA modification writers in PC

**Figure 1:**
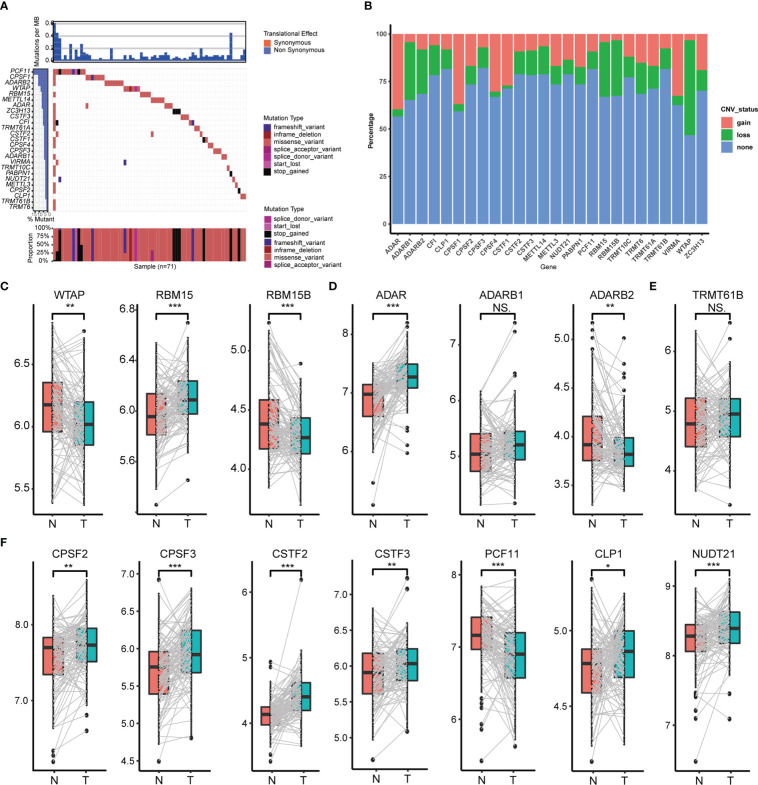
Transcriptional variation of four types of RNA modification writers in PC. **(A)** Mutation statuses of 26 RNA modification writers. **(B)** Somatic CNV of 26 RNA modification writers. Expression of m6A writers **(C)**, A-to-I writers **(D)**, m1A writers **(E)** and APA writers **(F)** between normal and PC tissues. (*p < 0.05; **p < 0.01; ***p < 0.001; ns, p > 0.05).

In total, we screened 26 RNA modification writers (7 m6A writers, 4 m1A writers, 12 APA writers and 3 A-I writers) from the published literature that were currently involved in this study (**Table S2**). To explore potential transcriptional variation in four types of RNA modification writers in PC, we evaluated the frequency of non-synonymous somatic mutations in 26 writers. As is shown in **Figure 1A**, 71 of 821(8.65%) samples gained mutations of RNA modification writers. Among them, the mutation frequency of PCF11 leads first and is followed by CPSF1, ADARB2, WTAP and RBM15. Although the comparison between overall survival among different mutation statuses of these writers was non-significant, PC patients with mutations had a shorter survival rate than those without mutations (**Figure S2E**), implying that transcriptional alteration may play a vital role in the progression of PC. We also assessed the somatic CNV of these writers. Intriguingly, ADAR, CPSF1, TRMT61B, CPSF4 and CSTF1 possessed an extensive prevalence of CNV gain, while WTAP, ADARB1, RBM15, RBM15B and CF1 had less CNV gain (**Figure 1B**).

To further perceive the relationship between the expression of these 26 RNA modification writers and the transcriptional variation status, we compared the mRNA expression of these writers between paired normal and PC tissues, and the most of writers were highly expressed in PC tissues (**Figures 1C–F**, **Figures S2A–D**). Those writers with CNV gain were significantly highly expressed in PC tissues and vice versa (e.g., ADAR, CPSF4). This suggests that CNV may be a crucial factor in the transcriptional process of these writers. Notwithstanding, several writers had widespread expression but with CNV loss. So, to examine the divergence between CNV and mRNA expression in PC, we concentrated on the subgroups of CNV status (CNV gain, CNV loss and CNV stable) among distinct writers which owned CNV loss in more than 20% of the samples. Undoubtedly, PC patients with CNV gain had higher mRNA expression than those with CNV loss in CPSF2, ADAR and TRMT61A (**Figures S2F–H**). All these analyses determined the robust bonds between the transcriptional scenery and mRNA expression in 26 RNA modification writers.

### 3.2 TIME and cancer hallmarks correlated with patterns of RNA modification writers

To probe into interrelations among these RNA modification writers, Pearson correlation coefficients were calculated among them, and we found that majority of the correlations were positive except for TRMT61A (**Figure S3A**). Also, Univariate Cox analysis showed that 10 of 26 writers (CSTF2, CPSF4, METTL3, NUDT21, ADARB2, PABPN1, CPSF1, VIRMA, METTL14 and CFI) were independent prognostic factors in PC patients (**Figure S3B**). Therefore, these detections led us to confirm that some crosstalk relationship probably exists in specific clusters of RNA modification.

**Figure 2:**
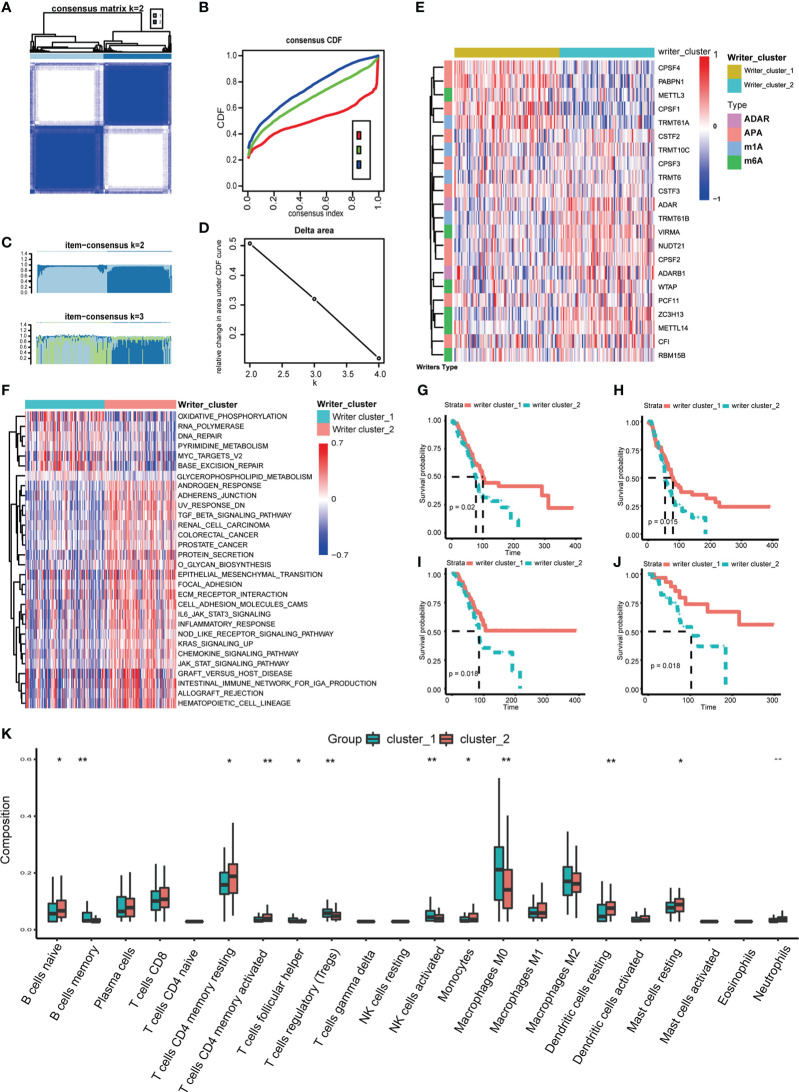
Distinct RNA modification patterns and correlated biological characteristics. Consensus heatmap **(A)**, CDF plot **(B)**, Item-Consensus plot **(C)** and area under CDF **(D)** of unsupervised consensus clustering in 26 RNA modification writers, the optimal k is 2. **(E)** Heatmap shows the expression of writers in distinct RNA modification patterns. **(F)** Heatmap of GSVA analysis shows specific enriched pathways in distinct RNA modification patterns. Comparison of OS **(G)**, DFI **(H)**, PFI **(I)** and DSS **(J)** between Writers_cluster_1 and Writers_cluster_2 pattern. **(K)** The differences in abundances of 22 types of immune cells between Writers_cluster_1 and Writers_cluster_2 pattern. (*p < 0.05; **p < 0.01).

Then, according to the screening standard mentioned above, unsupervised consensus clustering was performed to categorize PC patients into Writer_cluster_1 and Writer_cluster_2 based on the expression matrix of 22 selected RNA modification writers (**Figures 2A–E**; **Table S3**). It should be noticed that Writer_cluster_1 was charactered by the elevated expression of APA writers (CPSF1, CPSF4, PABPN1) and the over expression of m6A writers (METLL14, ZC3H13, VIRMA) always happened in Writer_cluster_2; besides, METTL3 was up regulated in Writer_cluster_1, confirmed the unique m6A regulating pattern in PC (**Figure 2E**). GSVA analysis was then applied to examine the molecular and biological functions of two distinct clusters of RNA modification. Writer_cluster_1 was notably enriched in DNA repair, base excision repair and RNA polymerization, while Writer_cluster_2 was markedly enriched in the period of tumorigenesis and immunoreactions, such as EMT, JAK/STAT signaling pathway and chemokine signaling pathway (**Figure 2F**). By the way, the prognostic endpoint was also appraised based on OS, DFI, PFI and DSS (**Figures 2G–J**). We found that the Writer_cluster_2 pattern of RNA modification exhibited a preferable survival rate than the Writer_cluster_1 pattern.

TIME of different RNA modification patterns was still considered in our study. CIBERSORT algorithm was performed to measure the component discrepancy between two distinct patterns of RNA modification (39). In the bulk, the expression profile of 26 RNA modification writers was highly correlated with tumor immune infiltration (**Figure S3C**). For instance, CF1, ZC3H13 and ADARB1 were prominently negatively correlated with NK cells resting. The abundances of 22 types of immune cells among two patterns were also quantified (**Figure 2K**). We noticed that the Writer_clsuter_1 pattern of RNA modification possessed higher infiltration of immunosuppressive cells (e.g., T cells regulatory), which was consistent with the poor prognostic outcome in **Figures 2G–J**.

### 3.3 Construction and validation of RNA modification writers signature

In order to further investigate the biological mechanism of two distinct RNA modification patterns, differential analysis was conducted to determine 215 DEGs related to different RNA modification statuses (**Table S4**). GO pathway analysis showed these DEGs enriched in several molecular functions including immunoglobulin receptor binding, signaling receptor and growth factor binding (**Figure S4A**), KEGG pathway analysis exhibited focal adhesion, TGF-β signaling pathway and ECM-receptor interaction were enriched (**Figure S4B**). For verifying the heterogeneity in regulation, we applied unsupervised consensus clustering based on these DEGs and stratified PC patients into DEG_cluster_A and DEG_cluster_B (**Figures S4C–F**). Consistent with the clustering of RNA modification writers, most of the patients clustered in Writer_cluster_1 corresponded to DEG_cluster_A, and Writer_cluster_2 to DEG_cluster_B (**Figure S4G** and **Table S3**, Fisher’s test p = 0.044).

**Figure 3:**
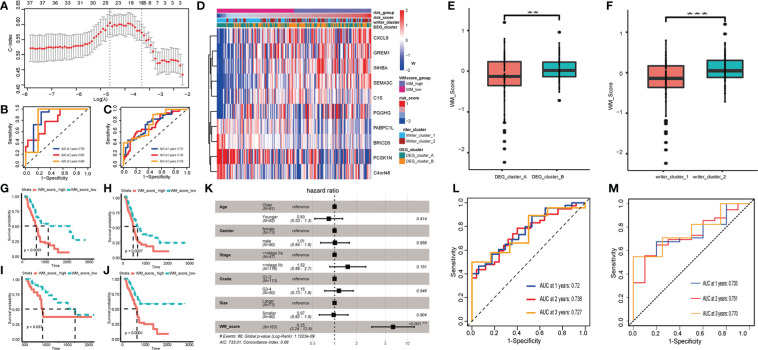
Construction and validation of RNA modification writers signature. **(A)** LASSO-Cox analysis was performed to constructed 10-DEGs based WM_Score model. AUC of WM_Score model in TCGA training **(B)** and testing **(C)** sets. **(D)** Heatmap visualizing the expression of 10 DEGs compared among Writer_cluster_1/2, DEG_cluster_A/B and WM_Score_high/low. Comparison of WM_Score between Writer_cluster_1 and _2 **(E)**, DEG_cluster_A and _B **(F)**. Comparison of OS **(G)**, DFI **(H)**, PFI **(I)** and DSS **(J)** between WM_Score_high and WM_Score_low group. **(K)** Multivariate Cox analysis shows WM_Score is significantly corresponded with prognosis, while the age, gender, stage, grade and tumor size proved to be nonsensical. AUC of WM_Score at 1, 2, 3 year in ICGC **(L)** and GEO **(M)** external validation set. (**p < 0.01; ***p < 0.001).

Patients in the TCGA cohort were randomly assigned to training and testing set at a ratio of 7:3. Based on DEGs, univariate Cox regression was performed to decrease redundancy and 38 prognosis-related DEGs remained. Next, we used the LASSO-Cox algorithm to distinguish two RNA modification patterns in the TCGA training set (**Figures 3A**, **S4H–I**). At last, a 10-DEGs (including CXCL9, GREM1, INHBA, SEMA3C, C1S, PGGHG, PABPC1L, BRICD5, PCSK1N and C4orf48) based model named WM_Score model was established, and PC patients were divided into WM_Score_high (WM_high) and WM_Score_low (WM_low) groups based on the median WM_Score. The forecasting capability of the WM_Score model for overall survival was evaluated by ROC curves, the AUC reached 0.722 at 1 year, 0.743 at 2 years, 0.756 at 3 years in the TCGA training set, and robustly validated in TCGA testing set (**Figures 3B, C**).

Coincidentally, these three clusters (Writer_cluster_1/2, DEG_cluster_A/B and WM_Score_high/low) indicated a high coherence through different calculative strategies (**Figure 3D**). As is shown in **Figure S4J**, 61.96% patients in Writer_cluster_1 overlap with patients in WM_Score_low group, 63.1% patients in Writer_cluster_2 overlap with patients in WM_Score_high group. 57.61% patients in DEG_cluster_A overlap with patients in WM_Score_low group, 59.52% patients in DEG_cluster_B overlap with patients in WM_Score_high group (**Figure S4K**). What’s more, we found that Writer_cluster_2 had higher WM_Score than Writer_cluster_1. By the same token, WM_Score of DEG_cluster_B were higher than DEG_cluster_A (**Figures 3E, F**).

Subsequently, the prognostic and clinicopathological features in WM_Score_high and WM_Score_low groups were compared. Patients with low WM_Score exhibited a preferable survival rate than those in the WM_Score_high group (**Figures 3G–J**).

In order to clarify the interdependency of WM_Score, we further conducted multivariate Cox analysis. The result manifested that the WM_Score significantly corresponded with prognosis, while the age, gender, stage, grade and tumor size proved to be nonsensical (**Figure 3K**). To further verify the reliability and practicability of the WM_Score model, ICGC and GEO external validation set was selected and AUC reached 0.72 (ICGC)/0.73 (GEO) at 1 year, 0.736 (ICGC)/0.751 (GEO) at 2 years, 0.727 (ICGC)/0.77 (GEO) at 3 years (**Figures 3L, M** and **Table S5**).

### 3.4 The interaction between WM_Score model and molecular biological features

**Figure 4:**
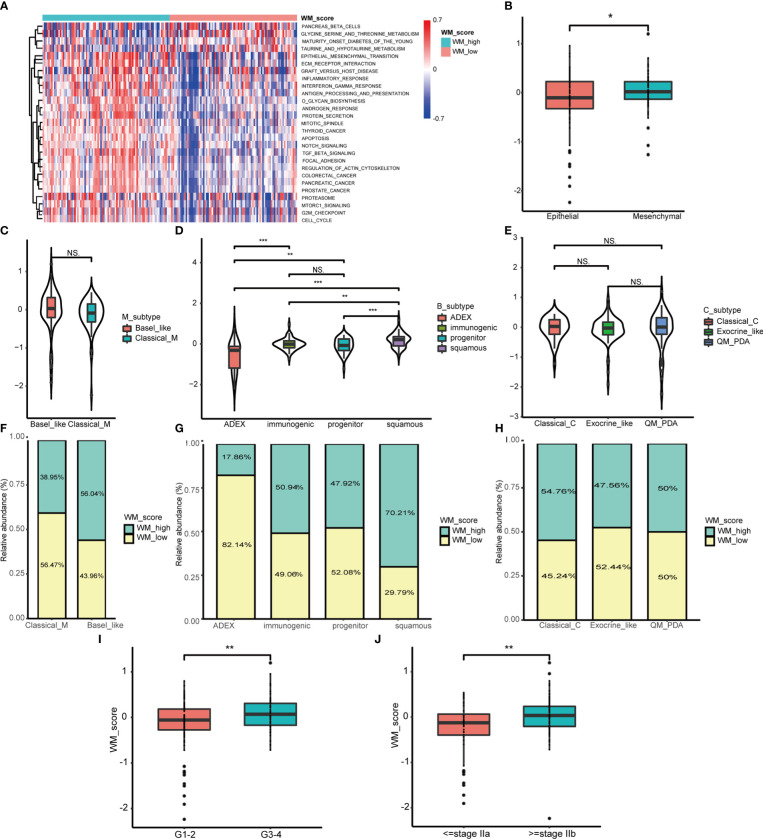
Biological characteristics of WM_Score model in PC. **(A)** GSVA analysis between WM_Score_high and _low group. **(B)** Differences in the WM_Score between mesenchymal trend and epithelial trend. WM_Score differences among Moffitt classification **(C)**, Collisson classification **(D)** and Bailey classification **(E)** based on patients in TCGA cohorts. **(F–H)** Overlap analysis of these three classifications and WM_Score based on the histogram of frequency distribution. Differences of WM_Score in specific grade **(I)** and stage **(J)** of patients in TCGA dataset. (*p < 0.05; **p < 0.01; ***p < 0.001; NS, p > 0.05).

To explore the functional role of distinct WM_Score subgroups mentioned above, GSVA analysis was applied. We found that the WM_Score_high group enriched in EMT, TGF-β, and mTORC1 signaling pathways (**Figure 4A**). For examining the correlation with EMT pathway, we computed the EMT score based on the expression of epithelial and mesenchymal marker genes. The stronger the tendency to mesenchymal, the higher the WM_Score, which may explain the poorer survival rate of the WM_Score_high group (**Figure 4B** and **Table S3**).

From published data, PC can be divided into three transcriptome classifications of molecular subtypes (MS) including Moffitt classification, Collisson classification and Bailey classification (40). Moffitt classification contains Classical and Basal-like subtypes, the latter subtype was confirmed to be linked to worse overall survival in PC (34). Collisson classification encompasses Classical with adhesion and epithelization, Exocrine-like with mesenchymal transition and quasi-mesenchymal (QM-PDA) with tumor cell derived digestive enzyme (35). Bailey classification includes aberrantly differentiated endocrine exocrine (ADEX) with KRAS activation and endocrine differentiation, Immunogenic with acquired immune suppression, Pancreatic progenitor with early pancreatic development and Squamous with hypermethylation of pancreatic endodermal cell-fate determining genes and have the worst prognosis (36). Based on the hallmark gene signatures in these three classifications of MS from the literature (34–36, 40), unsupervised consensus clustering was performed to classify PC patients into distinct MS (**Figures S5A–F** and **Table S3**). To assess the relationships between MS and WM_Score, we analyzed the WM_Score of MS in the TCGA dataset. Among overall nine MS, Basal-like, QM-PDA and Squamous subtypes acquired comparatively high WM_Score which may be associated with their unfavorable prognosis (**Figures 4C–E**).

We also implemented overlap analysis of these three classifications which were visualized by the histogram of distribution. In consistent, patients with a high degree of malignant MS (e.g. Basal-like and Squamous subtype) tended to be determined as WM_Score_high group and vice versa (**Figures 4F–H**). Furthermore, we found that WM_Score was higher in advanced PC than those in early grades and stages (**Figures 4I, J**), implying that this WM_Score model may be more sensitive to preclinical diagnostic. However, there were no significant WM_Score differences among old, gender and tumor size (**Figures S5G–I**).

### 3.5 Transcriptional and post-transcriptional regluation associated with WM_Score

RNA modifications have been historically identified as a transcriptional and post-transcriptional regulator, whereas the WM_Score model was conducted based on RNA modification writers. So, we concentrated on the transcriptional and post-transcriptional events (e.g. APA, m6A and m1A) related to WM_Score.

**Figure 5:**
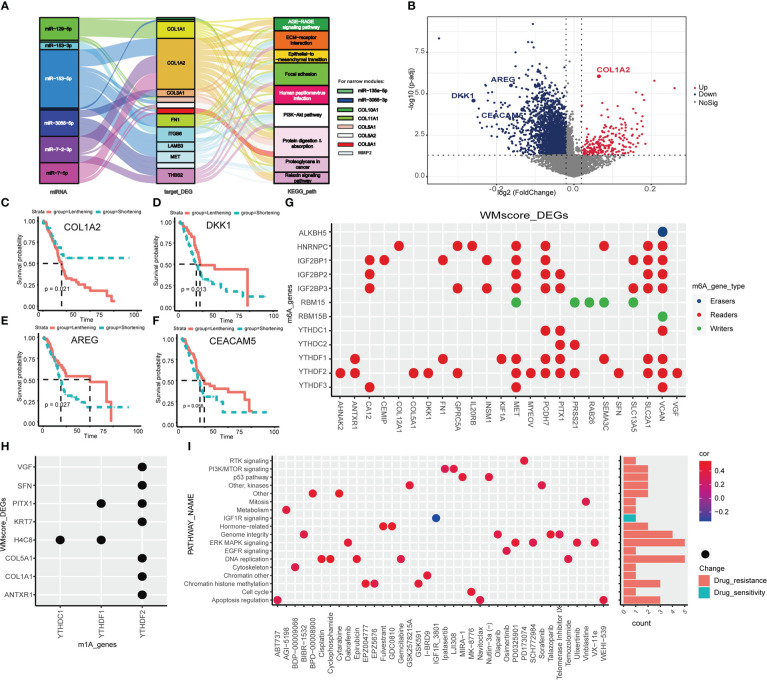
Transcriptional and post-transcriptional characteristics related to WM_Score. **(A)** Sankey diagram based on 8 miRNAs, 14 mRNAs and 9 enriched pathways. **(B)** The differences in the PDUI of each gene between WM_Score_high and WM_Score_low groups. Kaplan-Meler plot shows overall survival between 3’UTR lengthening and 3’UTR shortening of COL1A2 **(C)**, DKK1 **(D)**, AREG **(E)** and CEACAM5 **(F)**. Relationships between DEGs and m6A **(G)** or m1A **(H)** regulators *via* the RMVar database. **(I)** Signaling pathways targeted by drugs which are resistant or sensitivity to WM_Score.

It is well-established that APA promotes transcriptional alteration by providing mRNA with 3’UTRs where binding sites for miRNAs targeted (41), we proposed that two RNA modification statuses may have specific miRNA features based on the regulation of distinct writers. First of all, we performed differential analysis between WM_Score_high and _low group,42 miRNAs were screened out and pathway analysis of their target genes was operated (**Table S6**). Then, 8 miRNAs, 14 mRNAs and 9 enriched pathways were determined (**Figure 5A**; **Table S7**). For further identify the mechanism of RNA modification writers, we assessed the APA events of each gene in TCGA dataset to explore the post-transcriptional attributes. We identified the genes between two RNA modifications with distinct PDUI and found that most of genes with negative PDUI (shortening 3’UTR) enriched in the WM_Score_high group (**Figure 5B** and **Supplementary Table 8**). *Via* univariate Cox analysis, we selected 5 prognosis-related top genes (COL1A2, DKK1, AREG and CEACAM5) for verification. COL1A2 was with significantly lengthening 3’UTR in the WM_Score_high group, while DKK1, AREG and CEACAM5 were with markedly shortening 3’UTR. For those genes with lengthening 3’UTR, patients in the lengthening group had a worse survival rate than those in the shortening group, the same phenomenon was seen for genes with shortening 3’UTR (**Figures 5C–F**). As a side note, we can hypothesize that in the WM_Score_high group, the 3’UTR may work together with the miRNA-targeting system to facilitate the progression of PC.

To examine whether WM_Score was associated with m6A and m1A, we explored the corresponding relationships between DEGs and m6A or m1A regulators *via* the RMVar database. Among m6A and m1A regulators, readers binding with DEGs were the most, suggesting that WM_Score is definitely an integrated predictive model based on RNA modification writers (**Figures 5G, H** and **Table S9**-**10**).

### 3.6 Identification of potential compounds targeting the WM_Score model

Aiming at recognizing the impacts of WM_Score on drug sensitivity, Spearman correlation analysis was performed to compute the relationship index between WM_Score and the response to drugs based on the GDSC dataset. We found 38 potential compounds were markedly related to WM_Score (**Figure S6A** and **Table S11**). Among them, most of the compounds showed drug resistance on WM_Score, suggesting that patients with higher WM_Score probably lead to higher resistance to these targeted therapies, including Gemcitabine and Cisplatin, except for IGF1R_3801. Furthermore, we explored the targeted pathway of these compounds. The results showed that compounds targeted the WM_Score_low patients may regulate the MAPK, DNA replication and Genome integrity to strengthen the sensitivity of themselves (**Figure 5I**). In summary, the WM_Score proved to be an innovative therapeutic target for PC.

### 3.7 The WM_Score predicts distinct TIME and immunogenomic patterns

For examing the distinct TIME of the WM_Score model, CIBERSORT and ESTIMATE algorithms were applied based on the expression profile of patients in the TCGA dataset (**Figure S6B–E** and **Table S12**). No major differences were observed according to the abundances of 22 types of immune cells (42) between WM_Score_high and WM_Score_low group, but the WM_score was positively related to ESTIMATEScore, ImmnueScore and StromaScore, implying that the infiltration of immune cells in WM_Score model was highly abundant (**Figure S6C-E**).

**Figure 6:**
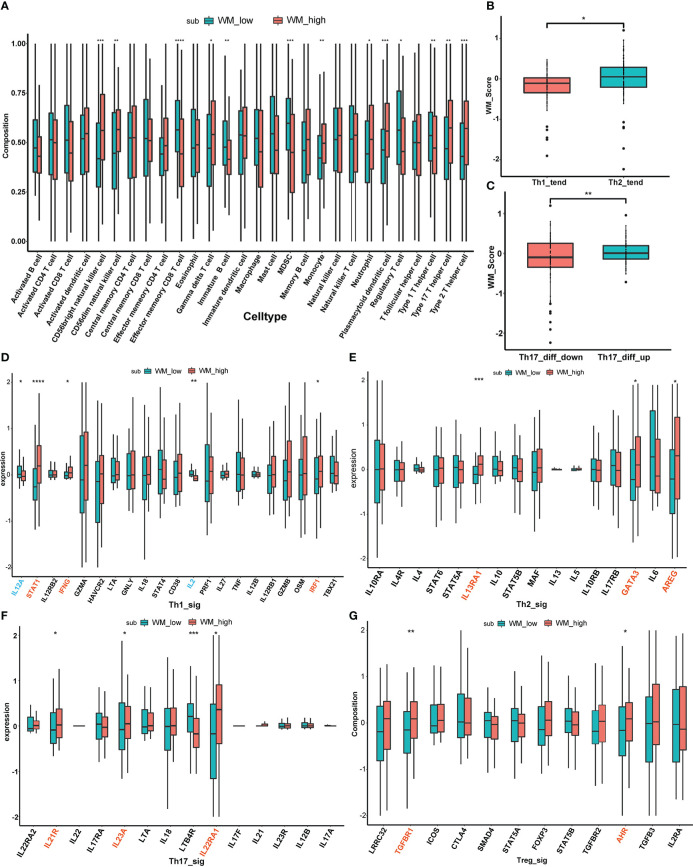
The relationship between WM_Score and immunogenomic patterns. **(A)** The immune cell infiltration between different WM_Score groups based on 28 stromal and immune cell types. Differences of WM_Score in Th1/Th2 trend **(B)** and Th17_diff_down/Th17_diff_up trend **(C)**. Expressional differences of distinct cytokines between WM_Score_high and _low group in Th1 **(D)**, Th2 **(E)**, Th17 **(F)**, Treg **(G)** signaling pathways. (*p < 0.05; **p < 0.01; ***p < 0.001; ****p > 0.0001).

To further validate the immune cell infiltration between different WM_Score subgroups, ssGSEA was performed based on 28 stromal and immune cell types according to the gene signature “LM22”, and multiple T cells infiltrations were found among two distinct WM_Score subgroups (e.g., Regulatory T cell, Th1 cell, Th2 cell and Th17 cell, **Figure 6A** and **Table S13**).

Considered PC is an immunologically cold tumor, exploration of immunogenomic patterns of PC was urgent to be emphasized based on WM_Score. Four types of T cells (Th1 cell, Th2 cell, Th17 cell and Treg cell) were involved in the following stratification analysis. Extensive evidence has documented that shifting Th1/Th2 balance toward to Th2 polarization may contribute to the tumor immune escape, while IL-12 can suppress Th2 differentiation and promote Th1 production and the case in IL-4 is the opposite (38, 43, 44). So, we extracted BIOCARTA_IL12_PATHAY and BIOCARTA_IL4_PATHWAY from the MSigDB database as background gene sets, ssGSEA scores based on these two gene sets were calculated separately. Then, the median value of the subtraction of these two ssGSEA scores was determined as a cutoff point to distinguish patients from Th1_trend and Th2_trend (**Table S14**). As is shown in **Figure 6B**, patients in Th2_trend have higher WM_Score than those in Th1_trend. On the other part, the Th17 cell differentiation gene set from the PathCards database was selected to access the differences between WM_Score subgroups, and patients were divided into Th17_diff_up and Th17_diff_down groups based on the median value of ssGSEA score computed *via* this gene set (**Table S14**). We found that patients in the Th17_diff_up group tend to gain higher WM_Score (**Figure 6C**). To further confirm the mechanism of the T cell infiltration in WM_Score, we screened several cytokine markers of those four types of T cells from published literature and compared their expression patterns between distinct WM_Score subgroups (38). As an overall perspective, WM_Score_high group gathered the more abundant infiltration of these T cells, including 3 cytokines (STAT1, IFNG and IRF1) in Th1 signaling (**Figure 6D**), 3 cytokines (IL13RA1, GATA3 and AREG) in Th2 signaling (**Figure 6E**), 3 cytokines (IL21R, IL23A and IL22RA1) in Th17 signaling (**Figure 6F**) and 2 cytokines (TGFBR1 and AHR) in Treg signaling (**Figure 6G**). At last, we evaluated the expression of common immune checkpoint markers to predict the response to immunotherapy (**Figure S6F**). The expression of PD-L1 was higher in the WM_Score_high group, indicating that patients in WM_Score high group may be more sensitive to immunotherapy.

## 4 Discussion

Owing to the emerging advancement of methods in whole-transcriptome sequencing and high-performance mass spectrometry, qualitative and quantitative detection in characterization of the RNA modification enzymes (e.g. writers and erasers) have achieved a breakthrough. As a crucial subunit facilitating catalysis and conjugation of RNA, writers plays an essential regulatory role in carcinogenesis, immune response and alternative splicing (45). Despite lots of efforts have been exerted to explore the systematic mechanism of writers in single RNA modification, the underlying interaction of multiple RNA modification writers in PC have not been clarified. Thus, our study focused on four types of RNA modification writers (m6A, m1A, APA and A-to-I) for further analyses. We first evaluated the transcriptional variation and mutational statuses of these RNA modification writers in PC. Then, based on the expression profile of these 26 writers and machine learning algorithm, two distinct RNA modification patterns were determined. To make the results more practical, we performed LASSO-Cox analysis to construct a score-based model, WM_Score model, and appraised the predictive capacity of RNA modification writers in different subgroups.

Via LASSO-Cox algorithm, WM_Score model was established based on 10 DEGs (CXCL9, GREM1, INHBA, SEMA3C, C1S, PGGHG, PABPC1L, BRICD5, PCSK1N and C4orf48). Linkage evidences suggested that most of these DEGs correlated with immunity and tumorigenesis. As a member of chemokine family, CXCL9 promotes the progression of PC *via* STAT3-dependent cytotoxic T lymphocyte suppression (46). GREM1 as functionally opposing BMP signaling pathway gene, was confirmed to promote the advancement and progression of colorectal cancer (47). In PC, INHBA/TGF-β regulatory network enhanced the stem cell-like properties and stromal microenvironment, leading to resistance to chemotherapy (48). SEMA3C regulated the autophagy process and tumor immune microenvironment, which in turn promoted pancreatic cancer cell growth (49). Silencing of C1S also resulted in decreased proliferation and viability of cancer cells and strengthened aggregation of T cells (50). Based on the median value of WM_Score, PC patients was divided into WM_Score_high and WM_Score_low group. We found that WM_Score_high group displayed the worse prognosis, and significantly enriched in EMT, TGF-β and mTORC1 pathways. It is generally known that EMT is essential for the initiation of metastasis in cancer progression (51), and TGF-β was one of the most well-known promoters of EMT-inducing transcription factors and a major contributor to immunosuppression (52). What’s more, mTOR was considered as a mediator in TGF-β pathway that intensified stemness and drug resistance in cancer (53). We can hypothesize that mTOR pathway activation induces TGF-β, in turn, enhanced the EMT signaling pathway in WM_Score_high group. This chain reaction may explain the poor survival rate of this group and validate the efficacy of our WM_Score model. More recently, Guo Y, et al (54) also discovered a six-gene prognostic signature (METTL16, WTAP, IGF2BP2, IGF2BP3, YTHDC2 and YTHDF2) in PC. No overlap was identified between the 10-gene WM_Score model we constructed and those previously defined. Besides, the methodology of signature construction we adopt is a more comprehensive way which included four types of RNA modifications. Taken together, our WM_Score model was identified to be superior or comparable to the previous defined signatures.

Known as “immune desert”, with limited T cell infiltration, the polarized PC immunity approached with a barrage of challenges (55). Together, TIME, cancer-associated fibroblasts (CAFs) and extracellular matrix proteins constitute the pro-tumor environment (56). Thus, we examined the TIME and immunogenomic patterns among distinct WM_Score subgroups, and WM_Score_high group was correlated with higher infiltration of immunosuppressive cells, including Th2 cell and Th17 cell, which was contributive to the systemic immune dysfunction. Considered the heterogeneous population of T cells in PC, we focused on three T helper cells (Th1, Th2 and Th17 cell) for subgroup analyses. Naive CD4+T cells can differentiate into two subsets: Th1 cells, which tend to enhance the proinflammatory responses and activate autoimmune responses; Th2 cells, which induce humoral immune responses by secreting IL-4, -5, -6, -9, -10 and -13 (56). In coculture studies, PC secreted IL-10 and TGF-β suppressed the development of Th1 responses, whereas promoted the shift from Th1 to Th2 trend that is correlated to worse survival (57). In addition to Th1 and Th2 cells, Th17 cells, characterized by secretion of IL-17, played a distinguished role in PC (58, 59). Although the function of Th17 remained controversial, emerging evidences have illustrated that Th17 seems to be a tumor promotor in the progression of PC (60). In consistent, patients in Th2_trend and Th17_diff_up subgroups achieved conspicuously higher WM_Score, bolstering the consequences of aforementioned works. On the other part, production of multiple cytokines by PC cells also resulted in the general immunosuppressive microenvironment of PC by swapping the balance from a Th1 to a Th2 status (61). Taken this phenomenon into consideration, we conducted an in-depth analysis of the relationship between four types of T cells (Th1, Th2, Th17 and Treg cells) signaling pathway and their distinct chemokines. The result elucidated that, in WM_Score_high group, high expression of chemokine STAT1, IFNG, IRF1, IL13RA1, GATA3 and AREG and low expression of chemokine IL12A and IL2 may act together to break the balance between Th1 and Th2 cells. By the same token, the increased expression of chemokine IL21R, IL23A, IL22RA1, TGFBR1 and AHR might coordinately regulate the recruitment of Th17 and Treg cells in WM_Score_high group. Given the complicated and heterogeneous regulatory mechanism in TIME mentioned above, this study provided a basis for future studies on RNA modification target therapy.

Additionally, as one type of RNA modifications, APA could regulate transcript stability by altering the miRNA-mediated activities at a post-transcriptional level. And the length of 3’UTR was utilized to measure the APA events, shortening 3’UTR generally related to oncogene activation and tumor metastasis (41). Based on this, we accessed the characteristics in miRNA-mediated RNA modification in the WM_Score model. In WM_Score_high group, the EMT, PI3K-Akt and protein digesting pathways targeted by DE miRNAs were enriched, and the length of 3’UTR was shorter than those in WM_Score_low group. We can present the hypotheses that for patients with higher WM_Score, the shortening 3’UTR of regulatory genes prevented the targeted accidents of miRNA, resulting in the normal transcription of these genes and leading to the development of PC. Finally, we explored the potential therapeutic targets of RNA modification writers in PC. The result shown that WM_score was mainly correlated with resistance to compounds targeted MAPK, DNA replication and Genome integrity pathways, and sensitivity to compounds targeted IGF1R signaling pathway. In other words, WM_Score_high group will benefit from the therapy which targeted IGF1R pathway, and several studies have already showed the target therapies against stromal insulin/IGF-1 pathway can have negative effects on PC progression (62). By the way, prediction of response to immunotherapy was considered in this study. All the above proved that WM_Score model based on distinct RNA modification pattern, was not only an efficient predictor to interpreter the transcriptional and post-transcriptional events, but also a classifier to access the clinical outcome of targeted therapy and immunotherapy, shedding new light on the adjuvant treatment for PC.

Nevertheless, this study still has several limitations. First, the interplay mechanism among four types of RNA modifications should be further validated *in vivo* and vitro. Second, as a consequence of limited patents receiving immunotherapy and the complexity and difficulty in assembling specimens, the association between WM_Score and immunotherapy response should be identified based on immunotherapy cohorts. Third, it should be noted that similar methodologies have been used in another study for colorectal cancer (CRC) (63). However, distinctions in results between the two studies and novelty of this study should be highlighted. 1) we discovered that the expression of APA writers CPSF1, CPSF4 and PABPN1 were enriched in Writer_Cluster1 with better prognosis whereas m6A writers METTL14, WTAP and ZC3H13 did the exact opposite, indicating that APA and m6A might be the decisive types of RNA modification in PC pathogenesis. 2) given the different cancer types and biological behavior between PC and CRC, our WM_score model that matched well with existing molecular subtypes of PC could provide a new sequencing-based tool for precise diagnosis/therapy and prognosis prediction as well as some novel molecular targets for future mechanism research of PC. 3) considering that the immunotherapy of CRC has been progressing much better than that in PC, our results indicating the interaction between RNA epigenetics and Th cells differentiation/polarization might exert unique effects on the mechanism research of PC immunity in the future. Fourth, this study did not fully integrate results of targeted drug screening with nanotechnology, which showed significant potential to improve treatment for PC patients (64). A combination of multi-omics research and nanotechnology held considerable promise in PC research in recent years. For instance, Kong C et al (65) developed an ultra-pH-sensitive micelle (UPSM) system targeting lysosomal catabolism activation of PC cells to achieve rapid drug release, they proved the therapeutic efficiency of this system through both transcriptional and amino acid profiling. Zhou S et al (66) screened miRNA biomarkers by exosome sequencing and designed a virus-mimicking fusogenic vesicle system to achieve accurate detection of these markers, improving diagnostic accuracy and therapeutic efficiency in PC patients. In the future, our research will strive to integrate our innovative WM_score model with the study of nanoparticle drug delivery, to improve the treatment of PC patients with poorer prognosis.

## 5 Conclusion

In conclusion, our integrated multi-omics analyses based on four types of RNA modification writers unveiled a convoluted regulatory network in immune infiltration and prognostic statuses of PC. We developed WM_Score model that served as a predictor of writers in transcriptional and post-transcriptional regulation, targeted therapies and immunotherapy. This study provided insights into the underlying interplay mechanism of RNA modifications, unfurling the novel therapeutic strategies for PC patients.

## Data availability statement

The datasets presented in this study can be found in online repositories. The names of the repository/repositories and accession number(s) can be found in the article/**Supplementary Material**.

## Author contributions

HZ and XY conceived and supervised the study. WG, DC, and JL analyzed the data. DC and WG wrote the draft. LZ, TX, XZ, and ZL revised and validated the manuscript. All authors read and approved the final manuscript.
