# Supplementary material for: Interplay of four types of RNA modification writers revealed distinct tumor microenvironment and biological characteristics in pancreatic cancer
Source: Front Immunol. 2022 Dec 19;13:1031184. doi: 10.3389/fimmu.2022.1031184 (PMC9806142; doi:10.3389/fimmu.2022.1031184)
Supplement: Supplementary file 1 [file DataSheet_1.pdf]

Figure S1. Flowchart of this study.

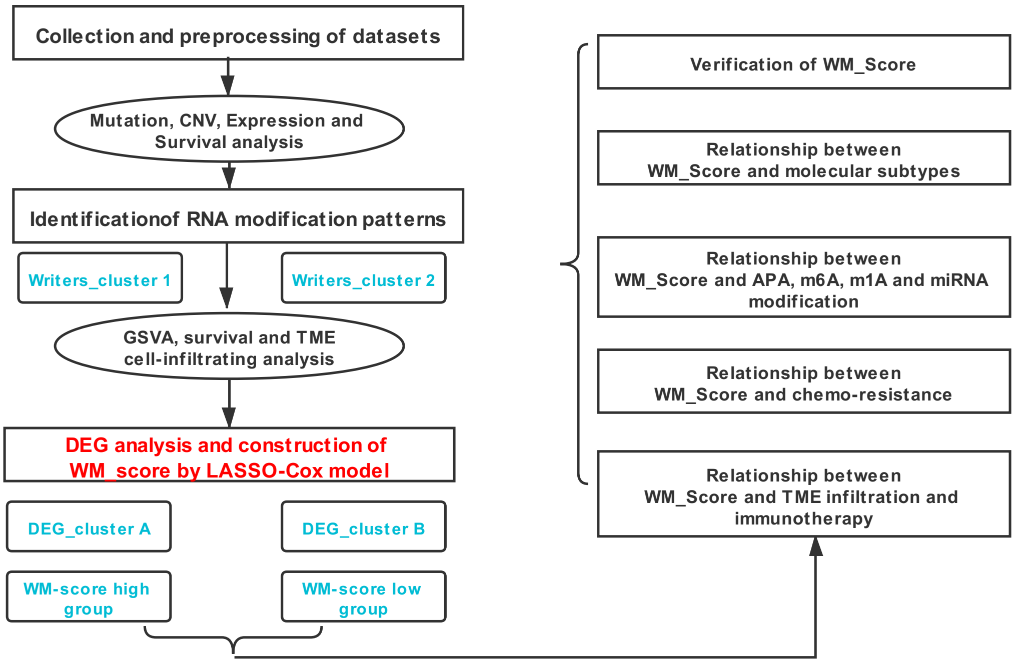

Figure S2. Expression pattern and CNV status of 26 RNA modification writers. Expression of A-to-I writers (A), m[1]A writers (B), m[6]A writers (C) and APA writers (D) between normal and PC tissues with non-significance (NS). The survival rate between mutation statuses of RNA modification writers (E). Expression of APA writers (F), A-to-I writers (G), m[6]A writers and m[1]A writers (H) among CNV gain, CNV loss and CNV stable subgroups.

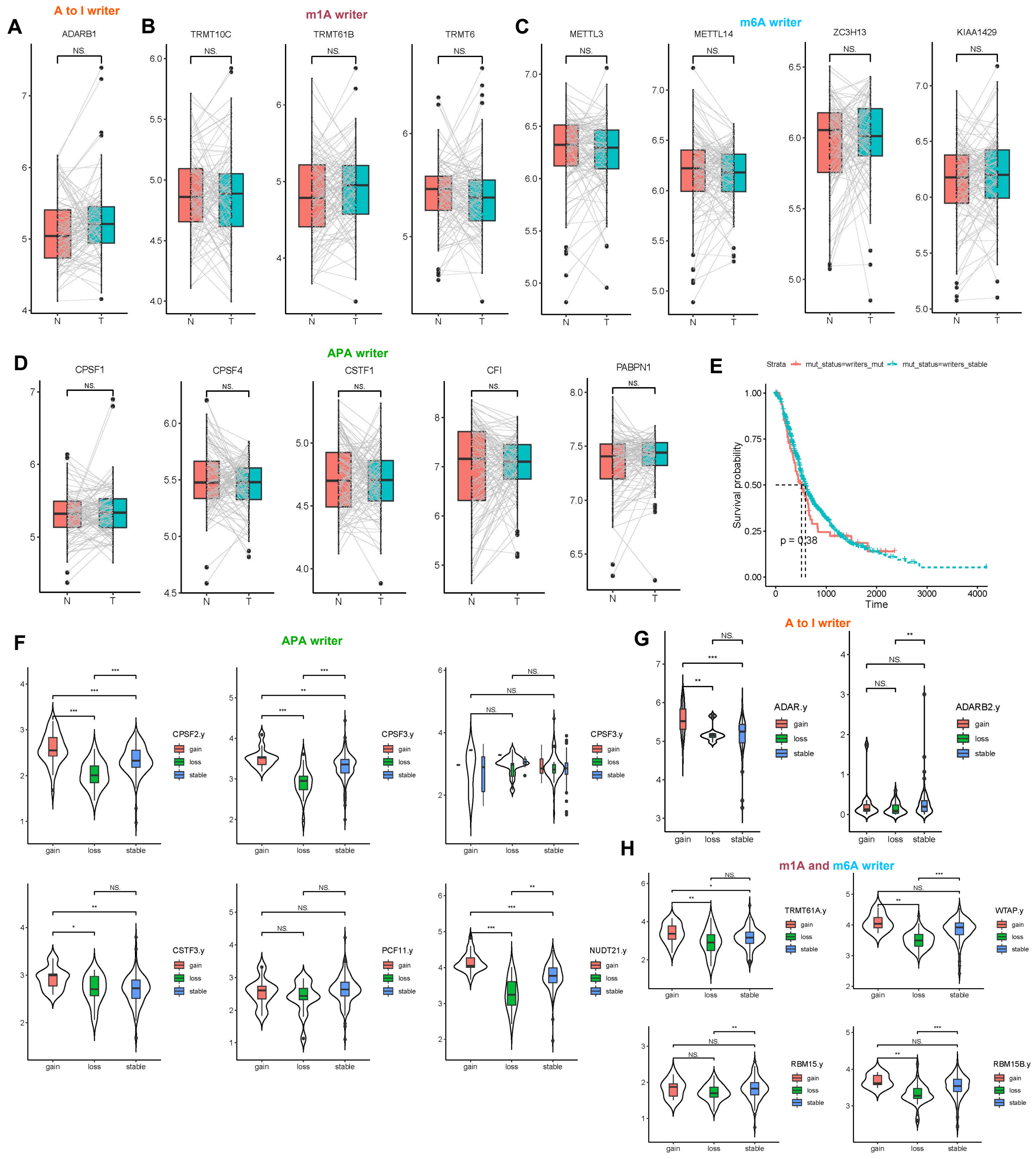

Figure S3. Correlation analysis of RNA modification writers at expression, prognosis and TIME levels. (A) Heatmap shows positive and negative correlation among 26 RNA modifications in PC. (B) Univariate Cox analysis shows 10 of 26 RNA modification writers play an independent role in prognosis of PC patients. (C) Correlation analysis between expression and tumor immune infiltration of 26 RNA modification writers.

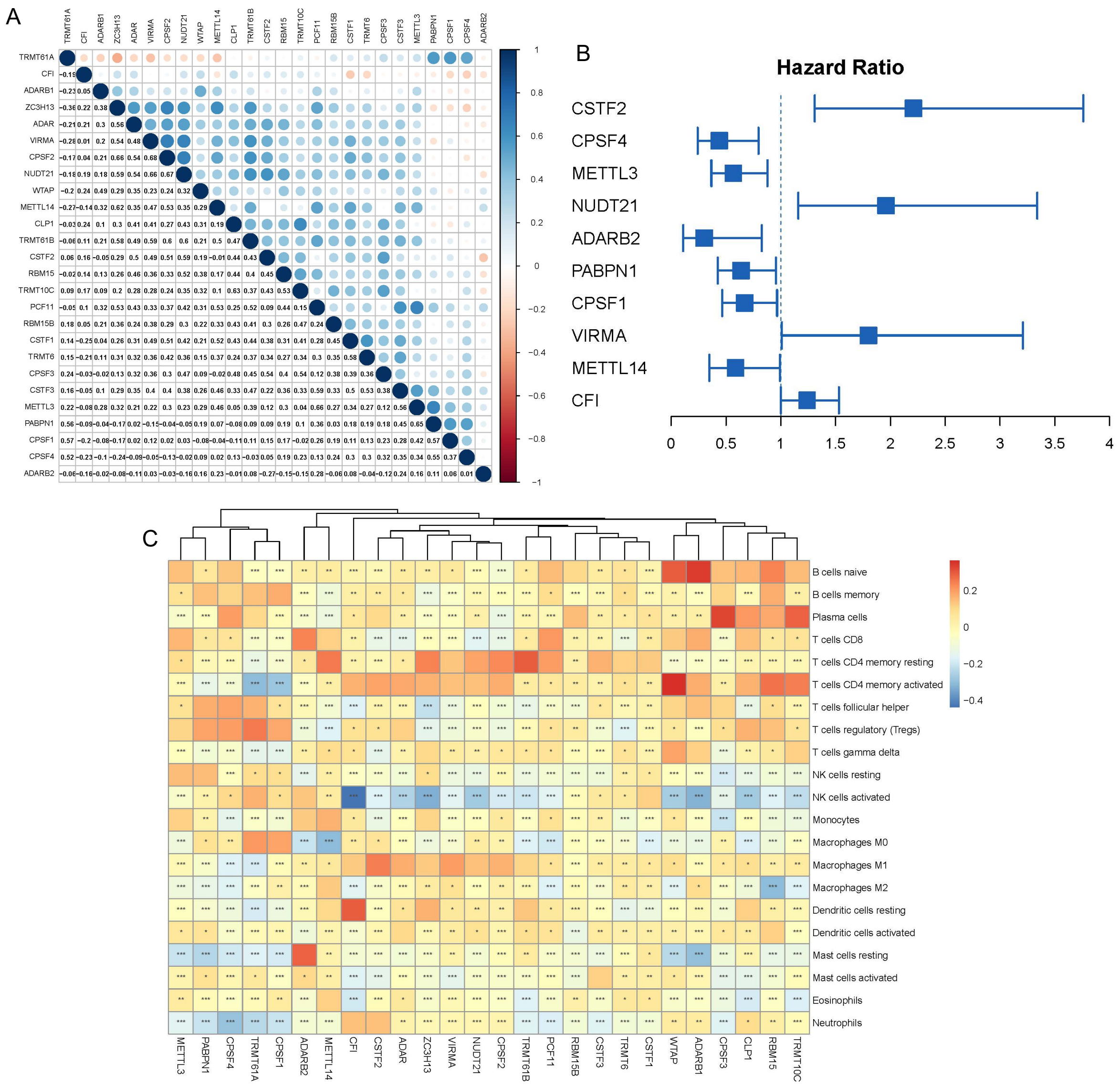

Figure S4. Construction and biological function of the DEG_cluster. GO (A) and KEGG (B) pathway analysis was conducted based on 222 DEGs. (C-F) Unsupervised consensus clustering algorithm classified PC patients into DEG_cluster_A and DEG_cluster_B. (G) Heatmap shows the expression of 222 DEGs in Writer_cluster_1/2, DEG_cluster_A/B. (H) Results of LASSO-Cox analysis based on 39 prognosis-related DEGs. (J-K) Overlap analysis based on Writer_cluster_1/2, DEG_cluster_A/B and WM_Score_high/low.

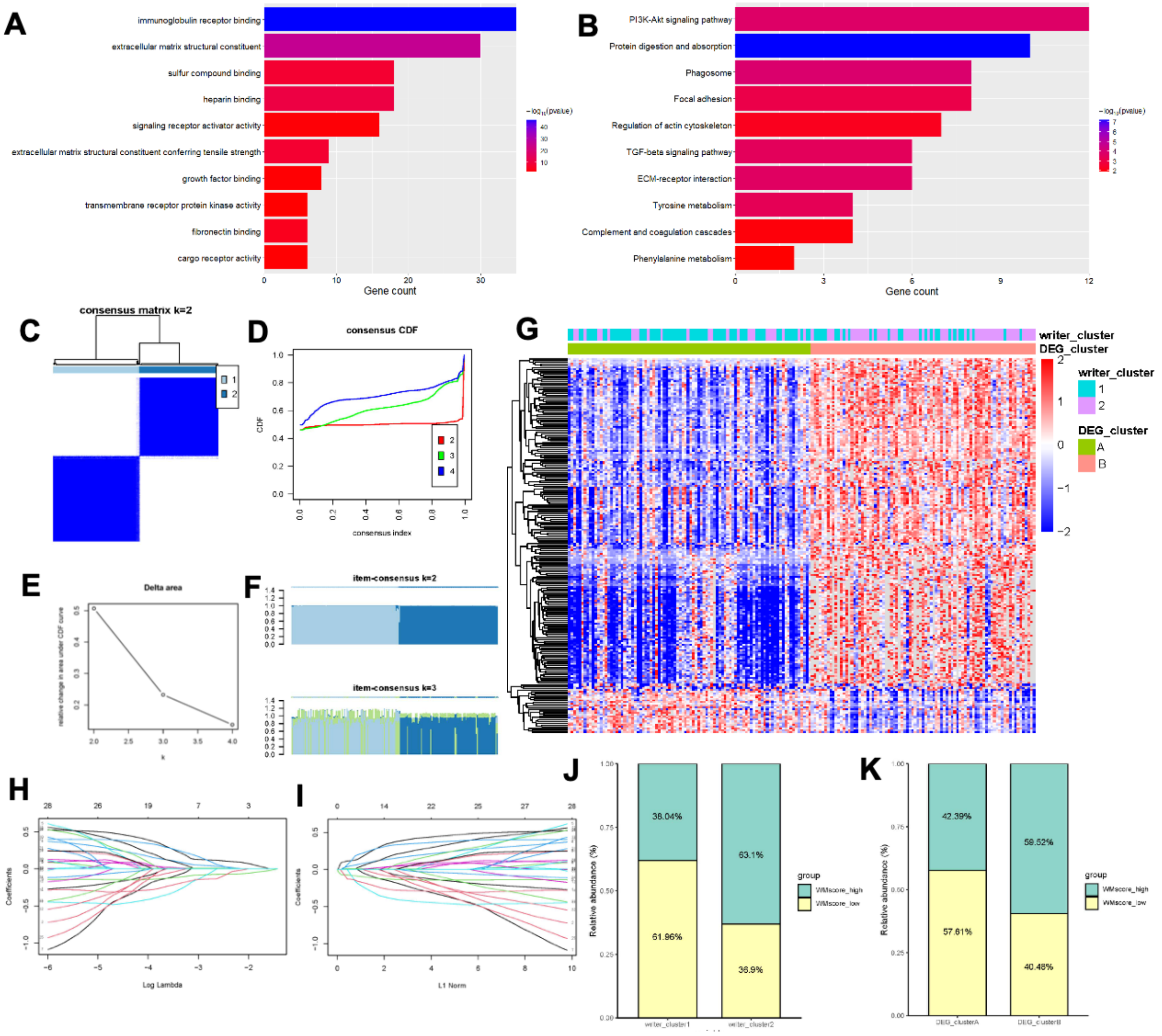

Figure S5. Molecular subtypes and clinical features of WM_Score model. Unsupervised consensus clustering performed based on Moffitt classification (A, B), Collisson classification (C, D) and Bailey classification (E, F). Differences in WM_Score in specific age (G), gender (H) and tumor site (I) of patients in TCGA dataset.

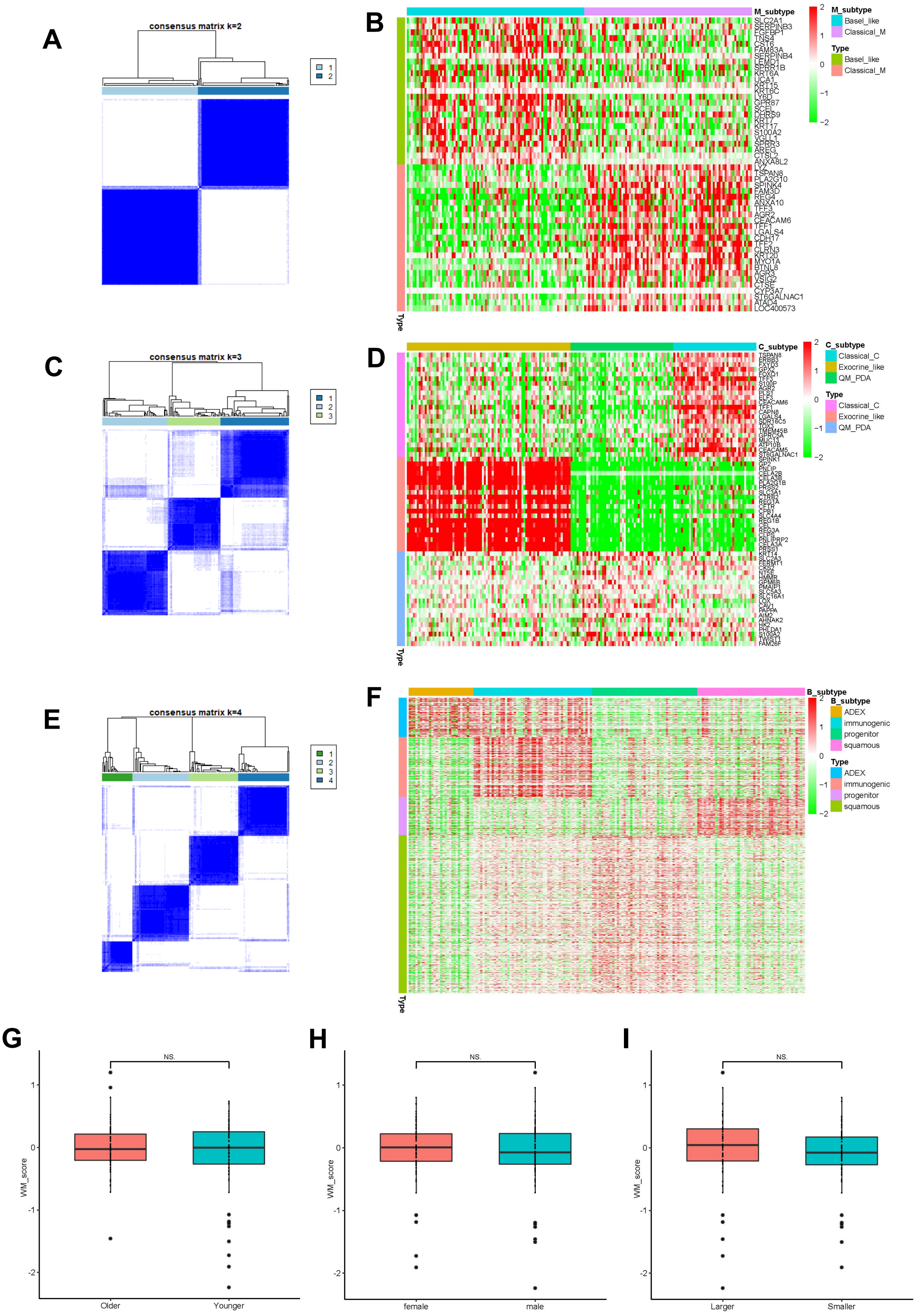

Figure S6. Correlation between WM_Score and drug sensitivity and TIME. (A) The realtionship between WM_Score and drug sensitivity. (B) The differences in abundances of 22 types of immune cells between WM_Socre_high and WM_Score_low group. CIBERSORT was performed to evaluate the correlation between WM_Score and ESTIMATEScore (C), ImmuneScore (D) and StromalScore (E). Expression of common immune checkpoint markers to predict the response to immunotherapy (F).

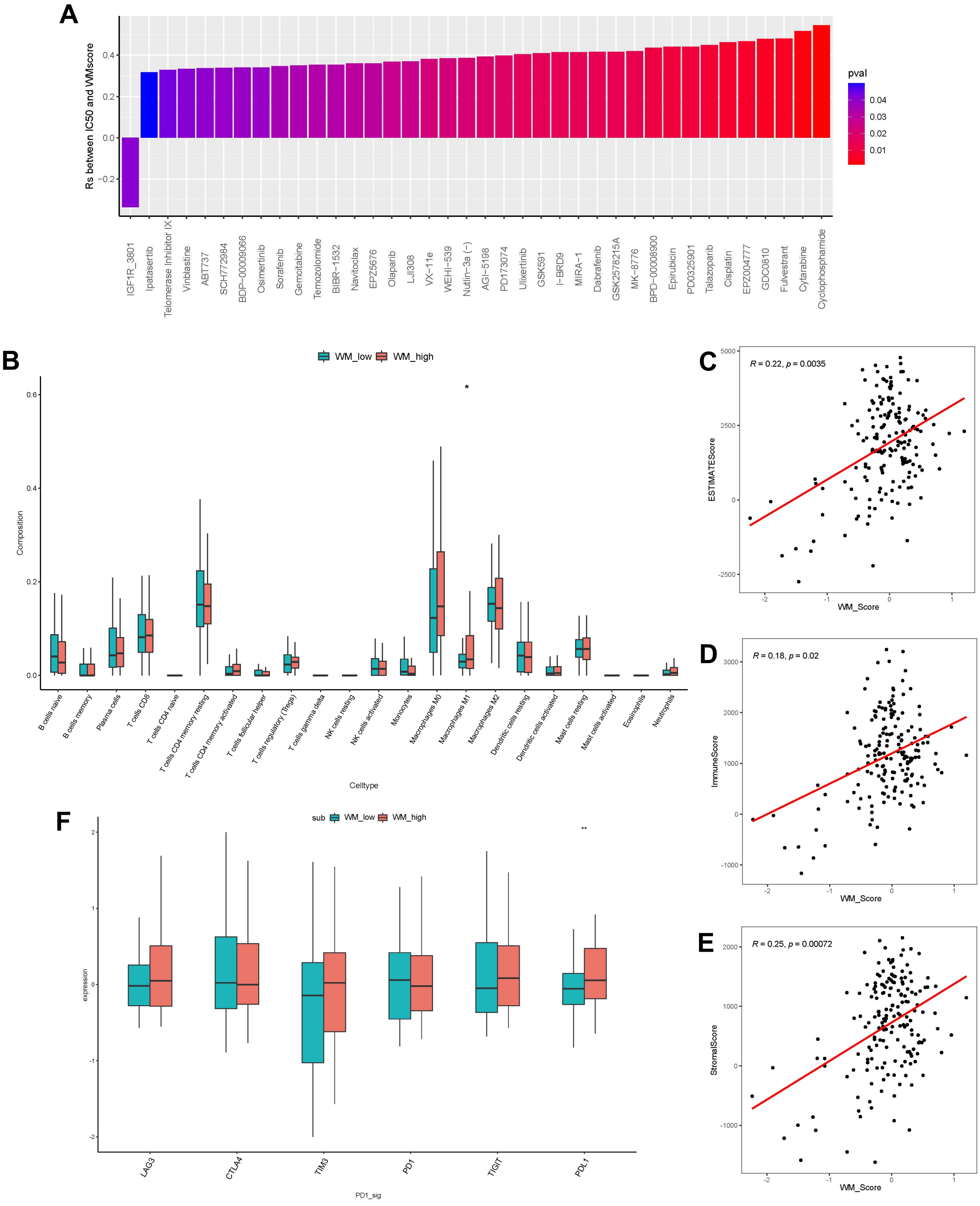

Supplementary Table 1: Sample Information and Usage of PAAD Datasets Included in This Study.

Accession number	Tumor samples	Normal samples	Survival data	Stage	Gender	Data type applied	Download link
TCGA_PAAD	178	4	181	stage i-iv: 178	F: 82, M:100	RNA-seq (HT-seq FPKM); Somatic mutation (MuTect2) and CNV (Gistic); Clinical information	https://xenabrowser.net/datapages/?cohort=GDC%20TCGA%20Pancreatic%20Cancer%20(PAAD)&removeHub=https%3A%2F%2Fxena.treehouse.gi.ucsc.edu%3A443
ICGC_AU_PAAD	83	0	83	NA	F: 37, M:46	RNA-seq (HT-seq FPKM); Somatic mutation (MuTect2); Clinical information	https://dcc.icgc.org/releases/current/Projects/PACA-AU
GSE62452	60	60 (Paired)	60	stage i-iv: 60	NA	HuGene-Array (GPL 6244, RMA Standardized)	https://www.ncbi.nlm.nih.gov/geo/query/acc.cgi
GSE28735	45	45 (Paired)	42	NA	NA	HuGene-Array (GPL 6244, RMA Standardized)	https://www.ncbi.nlm.nih.gov/geo/query/acc.cgi?acc=GSE28735
E-MTAB-3610	1018 Cancer cell lines; 29 PAAD cell lines	NA	NA	NA	NA	HG-U219 Array (GPL13667, RMA Standardized)	https://www.ebi.ac.uk/arrayexpress/experiments/E-MTAB-3610/

Supplementary Table 2: RNA Modification Writers Included in This Study.

Type	Writer
m6A	METTL3
m6A	METTL14
m6A	WTAP
m6A	RBM15
m6A	RBM15B
m6A	ZC3H13
m6A	KIAA1429
m1A	TRMT61A
m1A	TRMT10C
m1A	TRMT61B
m1A	TRMT6
APA	CPSF1
APA	CPSF2
APA	CPSF3
APA	CPSF4
APA	CSTF1
APA	CSTF2
APA	CSTF3
APA	CFI
APA	PCF11
APA	CLP1
APA	NUDT21
APA	PABPN1
A-I	ADAR
A-I	ADARB1
A-I	ADARB2

Supplementary Table 3: Samples clustering and Molecular Subtype Classification in TCGA_PAAD cohorts.

TCGA_ID	Writer_cluster	DEG_cluster	WM_score	WM_score_risk_group	B_subtype	C_subtype	M_subtype	E_score	M_score	EMT_group
TCGA.2J.AAB1.01A	writer_cluster_2	DEG_cluster_A	0.424727731	high_risk	progenitor	Classical_C	Classical_M	0.431452	-0.41345	Epithelial
TCGA.2J.AAB4.01A	writer_cluster_1	DEG_cluster_A	-0.242112711	low_risk	progenitor	Exocrine_like	Classical_M	0.193965	-0.11976	Epithelial
TCGA.2J.AAB6.01A	writer_cluster_1	DEG_cluster_A	-0.124526962	low_risk	squamous	QM_PDA	Basel_like	0.248189	0.228838	Epithelial
TCGA.2J.AAB8.01A	writer_cluster_2	DEG_cluster_B	0.17040116	high_risk	squamous	QM_PDA	Classical_M	0.150838	0.415438	Mesenchymal
TCGA.2J.AAB9.01A	writer_cluster_1	DEG_cluster_B	-0.207855341	low_risk	immunogenic	Exocrine_like	Basel_like	0.239508	0.211137	Epithelial
TCGA.2J.AABA.01A	writer_cluster_2	DEG_cluster_B	0.038353374	high_risk	progenitor	QM_PDA	Basel_like	0.348823	0.382656	Epithelial
TCGA.2J.AABE.01A	writer_cluster_1	DEG_cluster_A	-0.086436866	low_risk	squamous	Classical_C	Basel_like	0.038082	0.325697	Mesenchymal
TCGA.2J.AABF.01A	writer_cluster_1	DEG_cluster_B	0.099909052	high_risk	immunogenic	Exocrine_like	Classical_M	0.24122	0.031195	Epithelial
TCGA.2J.AABH.01A	writer_cluster_2	DEG_cluster_A	-0.331615859	low_risk	progenitor	Exocrine_like	Classical_M	0.25614	-0.25993	Epithelial
TCGA.2J.AABI.01A	writer_cluster_1	DEG_cluster_A	-0.081735142	low_risk	squamous	QM_PDA	Basel_like	0.076958	-0.17242	Epithelial
TCGA.2J.AABK.01A	writer_cluster_1	DEG_cluster_A	-0.542273192	low_risk	progenitor	Exocrine_like	Classical_M	0.332043	-0.26789	Epithelial
TCGA.2J.AABO.01A	writer_cluster_1	DEG_cluster_A	0.208319315	high_risk	squamous	Exocrine_like	Basel_like	0.347961	0.40808	Epithelial
TCGA.2J.AABP.01A	writer_cluster_1	DEG_cluster_B	0.030631565	high_risk	squamous	QM_PDA	Basel_like	-0.47361	0.289612	Mesenchymal
TCGA.2J.AABR.01A	writer_cluster_2	DEG_cluster_B	0.017397143	high_risk	immunogenic	Exocrine_like	Basel_like	0.143971	0.276804	Mesenchymal

TCGA.2J.AABT.01A	writer_cluster_2	DEG_cluster_A	-0.142834086	low_risk	ADEX	QM_PDA	Classical_M	-0.27483	0.219645	Mesenchymal
TCGA.2J.AABU.01A	writer_cluster_2	DEG_cluster_A	0.738088217	high_risk	squamous	QM_PDA	Basel_like	0.193237	0.251179	Epithelial
TCGA.2J.AABV.01A	writer_cluster_1	DEG_cluster_A	-0.393790632	low_risk	ADEX	Exocrine_like	Classical_M	-0.10605	-0.47332	Epithelial
TCGA.2L.AAQA.01A	writer_cluster_1	DEG_cluster_A	0.301824032	high_risk	progenitor	Classical_C	Classical_M	0.417184	-0.30301	Epithelial
TCGA.2L.AAQE.01A	writer_cluster_1	DEG_cluster_A	0.31806214	high_risk	squamous	QM_PDA	Basel_like	0.338826	-0.20868	Epithelial
TCGA.2L.AAQI.01A	writer_cluster_2	DEG_cluster_A	0.307416708	high_risk	progenitor	Exocrine_like	Classical_M	0.423645	-0.26073	Epithelial
TCGA.2L.AAQJ.01A	writer_cluster_1	DEG_cluster_A	0.432289224	high_risk	progenitor	Classical_C	Classical_M	0.474843	-0.10289	Epithelial
TCGA.2L.AAQL.01A	writer_cluster_1	DEG_cluster_A	0.135106552	high_risk	progenitor	Exocrine_like	Classical_M	0.326798	-0.26611	Epithelial
TCGA.2L.AAQM.01A	writer_cluster_1	DEG_cluster_A	-2.237296226	low_risk	ADEX	QM_PDA	Classical_M	-0.2958	-0.46304	Epithelial
TCGA.3A.A9I5.01A	writer_cluster_1	DEG_cluster_A	-1.193843421	low_risk	ADEX	Exocrine_like	Basel_like	0.068878	-0.28658	Epithelial
TCGA.3A.A9I7.01A	writer_cluster_1	DEG_cluster_B	0.107757264	high_risk	immunogenic	Exocrine_like	Classical_M	0.280328	0.354527	Epithelial
TCGA.3A.A9I9.01A	writer_cluster_1	DEG_cluster_A	-0.276057616	low_risk	ADEX	Exocrine_like	Basel_like	-0.11048	-0.46168	Epithelial
TCGA.3A.A9IB.01A	writer_cluster_1	DEG_cluster_A	0.213773989	high_risk	squamous	Exocrine_like	Basel_like	0.318911	0.363413	Epithelial
TCGA.3A.A9IC.01A	writer_cluster_1	DEG_cluster_A	-0.239823993	low_risk	squamous	QM_PDA	Basel_like	0.128814	0.43416	Mesenchymal
TCGA.3A.A9IH.01A	writer_cluster_2	DEG_cluster_A	0.414035746	high_risk	progenitor	Classical_C	Basel_like	0.419619	-0.20026	Epithelial
TCGA.3A.A9IJ.01A	writer_cluster_1	DEG_cluster_A	-1.262889887	low_risk	ADEX	QM_PDA	Basel_like	-0.42502	-0.32096	Mesenchymal

TCGA.3A.A9IL.01A	writer_cluster_1	DEG_cluster_A	-1.906319292	low_risk	ADEX	Exocrine_like	Basel_like	-0.46184	-0.4434	Epithelial
TCGA.3A.A9IN.01A	writer_cluster_1	DEG_cluster_A	-1.184111131	low_risk	ADEX	QM_PDA	Basel_like	-0.44744	-0.41787	Epithelial
TCGA.3A.A9IO.01A	writer_cluster_1	DEG_cluster_A	-1.503359503	low_risk	ADEX	Exocrine_like	Basel_like	-0.15456	-0.30115	Epithelial
TCGA.3A.A9IR.01A	writer_cluster_1	DEG_cluster_A	-1.724038996	low_risk	ADEX	QM_PDA	Basel_like	-0.17011	-0.4812	Epithelial
TCGA.3A.A9IS.01A	writer_cluster_1	DEG_cluster_A	-1.457359183	low_risk	ADEX	QM_PDA	Basel_like	-0.25156	-0.29286	Epithelial
TCGA.3A.A9IU.01A	writer_cluster_2	DEG_cluster_B	0.521300551	high_risk	squamous	Exocrine_like	Basel_like	0.378104	0.004051	Epithelial
TCGA.3A.A9IV.01A	writer_cluster_1	DEG_cluster_A	-1.074790604	low_risk	ADEX	QM_PDA	Classical_M	-0.41086	-0.29767	Mesenchymal
TCGA.3A.A9IX.01A	writer_cluster_2	DEG_cluster_B	-0.011409002	high_risk	immunogenic	Exocrine_like	Basel_like	0.047254	0.343499	Mesenchymal
TCGA.3A.A9IZ.01A	writer_cluster_1	DEG_cluster_A	0.162677514	high_risk	squamous	Exocrine_like	Basel_like	0.341156	-0.02418	Epithelial
TCGA.3A.A9J0.01A	writer_cluster_2	DEG_cluster_B	-0.108437035	low_risk	squamous	Classical_C	Basel_like	0.301455	0.219646	Epithelial
TCGA.3E.AAAY.01A	writer_cluster_1	DEG_cluster_B	-0.324536777	low_risk	immunogenic	Exocrine_like	Classical_M	0.158053	0.063352	Epithelial
TCGA.3E.AAAZ.01A	writer_cluster_1	DEG_cluster_B	-0.124128044	low_risk	progenitor	Exocrine_like	Classical_M	0.370645	0.137518	Epithelial
TCGA.F2.6879.01A	writer_cluster_2	DEG_cluster_A	0.249919185	high_risk	progenitor	Classical_C	Classical_M	0.224774	0.213806	Epithelial
TCGA.F2.6880.01A	writer_cluster_2	DEG_cluster_A	-0.351252024	low_risk	ADEX	Exocrine_like	Basel_like	-0.35425	-0.48421	Epithelial

TCGA.F2.7273.01A	writer_cluster_2	DEG_cluster_B	0.013666123	high_risk	immunogenic	Exocrine_like	Classical_M	-0.44622	0.450893	Mesenchymal
TCGA.F2.7276.01A	writer_cluster_2	DEG_cluster_B	-0.008928099	high_risk	immunogenic	Exocrine_like	Basel_like	-0.09327	0.445695	Mesenchymal
TCGA.F2.A44G.01A	writer_cluster_2	DEG_cluster_A	-0.139727823	low_risk	squamous	Exocrine_like	Basel_like	0.280949	0.207878	Epithelial
TCGA.F2.A44H.01A	writer_cluster_2	DEG_cluster_A	-0.192761932	low_risk	ADEX	Exocrine_like	Classical_M	-0.36776	0.475625	Mesenchymal
TCGA.F2.A7TX.01A	writer_cluster_2	DEG_cluster_B	0.957024047	high_risk	immunogenic	Exocrine_like	Basel_like	0.287031	-0.27254	Epithelial
TCGA.F2.A8YN.01A	writer_cluster_1	DEG_cluster_A	0.325391408	high_risk	squamous	Classical_C	Basel_like	0.066174	0.25771	Mesenchymal
TCGA.FB.A4P5.01A	writer_cluster_1	DEG_cluster_B	0.018095362	high_risk	immunogenic	Exocrine_like	Basel_like	-0.11516	0.353223	Mesenchymal
TCGA.FB.A4P6.01A	writer_cluster_1	DEG_cluster_A	-0.368175989	low_risk	ADEX	Exocrine_like	Classical_M	0.039923	0.04927	Epithelial
TCGA.FB.A545.01A	writer_cluster_1	DEG_cluster_A	-0.234493472	low_risk	squamous	QM_PDA	Basel_like	0.317683	0.1633	Epithelial
TCGA.FB.A5VM.01A	writer_cluster_1	DEG_cluster_A	-0.602403212	low_risk	squamous	QM_PDA	Basel_like	0.090208	-0.09011	Epithelial
TCGA.FB.A78T.01A	writer_cluster_1	DEG_cluster_A	-0.385263957	low_risk	progenitor	Exocrine_like	Classical_M	0.366042	-0.4868	Epithelial
TCGA.FB.A7DR.01A	writer_cluster_2	DEG_cluster_B	0.324713836	high_risk	squamous	Exocrine_like	Basel_like	0.235541	0.405047	Mesenchymal
TCGA.FB.AAPP.01A	writer_cluster_1	DEG_cluster_A	-0.265892604	low_risk	progenitor	Classical_C	Classical_M	0.358595	-0.48912	Epithelial
TCGA.FB.AAPQ.01A	writer_cluster_2	DEG_cluster_A	0.056543663	high_risk	progenitor	Classical_C	Classical_M	0.260038	-0.30426	Epithelial
TCGA.FB.AAPS.01A	writer_cluster_1	DEG_cluster_B	-0.103559256	low_risk	immunogenic	Exocrine_like	Basel_like	-0.46641	0.495968	Mesenchymal

TCGA.FB.AAPU.01A	writer_cluster_2	DEG_cluster_A	-0.324783939	low_risk	progenitor	Classical_C	Classical_M	0.406777	-0.48938	Epithelial
TCGA.FB.AAPY.01A	writer_cluster_1	DEG_cluster_A	-0.270938866	low_risk	progenitor	Exocrine_like	Classical_M	0.280145	-0.19075	Epithelial
TCGA.FB.AAPZ.01A	writer_cluster_2	DEG_cluster_A	-0.203375627	low_risk	squamous	Exocrine_like	Basel_like	0.364138	-0.15626	Epithelial
TCGA.FB.AAQ0.01A	writer_cluster_1	DEG_cluster_A	-0.322120524	low_risk	progenitor	Classical_C	Classical_M	0.379025	-0.42364	Epithelial
TCGA.FB.AAQ1.01A	writer_cluster_1	DEG_cluster_A	0.283811716	high_risk	ADEX	Exocrine_like	Basel_like	0.420533	-0.3062	Epithelial
TCGA.FB.AAQ2.01A	writer_cluster_1	DEG_cluster_A	0.320428618	high_risk	squamous	QM_PDA	Basel_like	0.320763	-0.14256	Epithelial
TCGA.FB.AAQ3.01A	writer_cluster_1	DEG_cluster_A	0.188841845	high_risk	progenitor	Exocrine_like	Classical_M	0.282142	0.046163	Epithelial
TCGA.FB.AAQ6.01A	writer_cluster_1	DEG_cluster_A	-0.058595023	low_risk	progenitor	Classical_C	Classical_M	0.343345	-0.45944	Epithelial
TCGA.H6.8124.01A	writer_cluster_2	DEG_cluster_A	0.350459823	high_risk	squamous	QM_PDA	Basel_like	0.247882	0.302789	Epithelial
TCGA.H6.A45N.01A	writer_cluster_1	DEG_cluster_B	-0.035133123	low_risk	immunogenic	Exocrine_like	Classical_M	0.204658	0.324464	Mesenchymal
TCGA.H8.A6C1.01A	writer_cluster_1	DEG_cluster_A	-0.274217478	low_risk	progenitor	Exocrine_like	Classical_M	0.13349	-0.05006	Epithelial
TCGA.HV.A5A3.01A	writer_cluster_1	DEG_cluster_A	0.039662577	high_risk	squamous	Classical_C	Basel_like	0.262935	-0.42025	Epithelial
TCGA.HV.A5A4.01A	writer_cluster_1	DEG_cluster_B	-0.1520726	low_risk	progenitor	Classical_C	Classical_M	0.162445	0.260818	Epithelial
TCGA.HV.A5A5.01A	writer_cluster_1	DEG_cluster_B	-0.502563382	low_risk	immunogenic	Exocrine_like	Classical_M	0.100003	0.132081	Epithelial
TCGA.HV.A5A6.01A	writer_cluster_1	DEG_cluster_A	-0.361981249	low_risk	squamous	Classical_C	Classical_M	0.243914	0.167775	Epithelial

TCGA.HV.A7OL.01A	writer_cluster_1	DEG_cluster_A	0.447943977	high_risk	progenitor	Classical_C	Basel_like	0.29874	-0.42148	Epithelial
TCGA.HV.A7OP.01A	writer_cluster_1	DEG_cluster_A	-0.714738825	low_risk	progenitor	Classical_C	Classical_M	0.066207	-0.49536	Epithelial
TCGA.HV.AA8V.01A	writer_cluster_1	DEG_cluster_B	-0.263185268	low_risk	squamous	Classical_C	Basel_like	-0.13806	0.489147	Mesenchymal
TCGA.HV.AA8X.01A	writer_cluster_2	DEG_cluster_A	0.130737073	high_risk	progenitor	Classical_C	Classical_M	0.204296	-0.47239	Epithelial
TCGA.HZ.7289.01A	writer_cluster_2	DEG_cluster_A	0.283104224	high_risk	progenitor	Classical_C	Classical_M	0.367369	-0.3172	Epithelial
TCGA.HZ.7918.01A	writer_cluster_2	DEG_cluster_B	0.300466821	high_risk	immunogenic	Exocrine_like	Classical_M	-0.10812	0.29941	Mesenchymal
TCGA.HZ.7919.01A	writer_cluster_2	DEG_cluster_B	0.300944829	high_risk	progenitor	Exocrine_like	Classical_M	0.359338	0.334599	Epithelial
TCGA.HZ.7920.01A	writer_cluster_2	DEG_cluster_B	-0.132221467	low_risk	immunogenic	QM_PDA	Basel_like	-0.24866	0.203293	Mesenchymal
TCGA.HZ.7922.01A	writer_cluster_2	DEG_cluster_B	0.169002732	high_risk	immunogenic	Exocrine_like	Classical_M	0.241204	0.46242	Mesenchymal
TCGA.HZ.7923.01A	writer_cluster_2	DEG_cluster_B	-0.434820794	low_risk	immunogenic	Exocrine_like	Classical_M	-0.2216	0.380878	Mesenchymal
TCGA.HZ.7924.01A	writer_cluster_2	DEG_cluster_B	-0.385435194	low_risk	progenitor	Classical_C	Classical_M	0.364699	-0.15883	Epithelial
TCGA.HZ.7925.01A	writer_cluster_2	DEG_cluster_B	0.154074728	high_risk	squamous	QM_PDA	Basel_like	-0.25272	0.47775	Mesenchymal
TCGA.HZ.7926.01A	writer_cluster_2	DEG_cluster_B	0.197821273	high_risk	immunogenic	Exocrine_like	Classical_M	0.426077	0.066451	Epithelial
TCGA.HZ.8001.01A	writer_cluster_2	DEG_cluster_B	-0.207611115	low_risk	immunogenic	Exocrine_like	Basel_like	-0.25284	0.443274	Mesenchymal
TCGA.HZ.8002.01A	writer_cluster_2	DEG_cluster_B	-0.135950598	low_risk	immunogenic	Exocrine_like	Basel_like	-0.02372	0.401612	Mesenchymal
TCGA.HZ.8003.01A	writer_cluster_2	DEG_cluster_B	0.000802351	high_risk	immunogenic	Exocrine_like	Basel_like	-0.25845	0.101795	Mesenchymal

TCGA.HZ.8005.01A	writer_cluster_2	DEG_cluster_A	1.197784865	high_risk	squamous	QM_PDA	Basel_like	0.208678	0.434878	Mesenchymal
TCGA.HZ.8315.01A	writer_cluster_2	DEG_cluster_B	0.538036879	high_risk	immunogenic	Exocrine_like	Basel_like	0.269979	0.39056	Mesenchymal
TCGA.HZ.8317.01A	writer_cluster_2	DEG_cluster_A	0.009847626	high_risk	ADEX	Exocrine_like	Classical_M	-0.11056	-0.03817	Epithelial
TCGA.HZ.8519.01A	writer_cluster_2	DEG_cluster_B	-0.515360162	low_risk	ADEX	QM_PDA	Classical_M	-0.4156	0.203562	Mesenchymal
TCGA.HZ.8636.01A	writer_cluster_2	DEG_cluster_B	0.57519342	high_risk	squamous	QM_PDA	Basel_like	0.315692	0.45493	Mesenchymal
TCGA.HZ.8637.01A	writer_cluster_2	DEG_cluster_B	-0.043996833	low_risk	immunogenic	QM_PDA	Classical_M	-0.25279	0.088528	Mesenchymal
TCGA.HZ.8638.01A	writer_cluster_2	DEG_cluster_B	-0.112986514	low_risk	progenitor	Classical_C	Classical_M	0.279998	-0.13784	Epithelial
TCGA.HZ.A49G.01A	writer_cluster_1	DEG_cluster_B	-0.261842989	low_risk	immunogenic	Exocrine_like	Classical_M	-0.07049	0.061931	Mesenchymal
TCGA.HZ.A49H.01A	writer_cluster_1	DEG_cluster_B	-0.281201302	low_risk	immunogenic	Exocrine_like	Classical_M	-0.23254	0.027506	Mesenchymal
TCGA.HZ.A49I.01A	writer_cluster_1	DEG_cluster_A	-0.104349162	low_risk	progenitor	Classical_C	Classical_M	0.254469	0.037793	Epithelial
TCGA.HZ.A4BH.01A	writer_cluster_1	DEG_cluster_B	0.262625693	high_risk	squamous	QM_PDA	Basel_like	0.228936	0.372316	Mesenchymal
TCGA.HZ.A4BK.01A	writer_cluster_1	DEG_cluster_B	-0.002959487	high_risk	progenitor	Exocrine_like	Classical_M	0.349783	-0.08797	Epithelial
TCGA.HZ.A77O.01A	writer_cluster_1	DEG_cluster_A	0.799852419	high_risk	ADEX	Exocrine_like	Basel_like	0.179243	-0.30931	Epithelial
TCGA.HZ.A77P.01A	writer_cluster_2	DEG_cluster_A	-0.128292268	low_risk	ADEX	Exocrine_like	Classical_M	-0.07688	0.051161	Mesenchymal
TCGA.HZ.A77Q.01A	writer_cluster_2	DEG_cluster_B	0.44023879	high_risk	squamous	QM_PDA	Basel_like	-0.13957	0.435488	Mesenchymal

TCGA.HZ.A8P1.01A	writer_cluster_1	DEG_cluster_A	-0.129718222	low_risk	progenitor	Classical_C	Classical_M	0.435555	-0.47425	Epithelial
TCGA.HZ.A9TJ.01A	writer_cluster_1	DEG_cluster_A	-0.612000525	low_risk	progenitor	Classical_C	Classical_M	0.420417	-0.26899	Epithelial
TCGA.IB.7644.01A	writer_cluster_2	DEG_cluster_A	0.011400152	high_risk	progenitor	Classical_C	Classical_M	0.303301	0.161435	Epithelial
TCGA.IB.7645.01A	writer_cluster_2	DEG_cluster_B	0.183745424	high_risk	immunogenic	QM_PDA	Classical_M	-0.16658	0.48113	Mesenchymal
TCGA.IB.7646.01A	writer_cluster_2	DEG_cluster_B	0.580889834	high_risk	squamous	QM_PDA	Basel_like	0.108243	0.419145	Mesenchymal
TCGA.IB.7649.01A	writer_cluster_2	DEG_cluster_B	0.040740459	high_risk	immunogenic	Exocrine_like	Classical_M	-0.04854	0.292219	Mesenchymal
TCGA.IB.7651.01A	writer_cluster_2	DEG_cluster_B	0.455729725	high_risk	immunogenic	Exocrine_like	Classical_M	-0.22713	0.418722	Mesenchymal
TCGA.IB.7652.01A	writer_cluster_2	DEG_cluster_B	0.152791466	high_risk	immunogenic	Exocrine_like	Basel_like	0.093754	0.347221	Mesenchymal
TCGA.IB.7654.01A	writer_cluster_2	DEG_cluster_B	0.231666754	high_risk	ADEX	Exocrine_like	Basel_like	-0.04095	0.389358	Mesenchymal
TCGA.IB.7885.01A	writer_cluster_2	DEG_cluster_B	0.317448886	high_risk	squamous	QM_PDA	Basel_like	0.233467	0.430008	Mesenchymal
TCGA.IB.7886.01A	writer_cluster_2	DEG_cluster_B	0.38464341	high_risk	progenitor	Classical_C	Classical_M	0.133398	0.392315	Mesenchymal
TCGA.IB.7887.01A	writer_cluster_2	DEG_cluster_B	0.695622606	high_risk	squamous	QM_PDA	Basel_like	0.186644	0.361632	Mesenchymal
TCGA.IB.7888.01A	writer_cluster_2	DEG_cluster_B	0.017269424	high_risk	immunogenic	Classical_C	Classical_M	-0.42612	0.432158	Mesenchymal
TCGA.IB.7889.01A	writer_cluster_2	DEG_cluster_B	-0.012389419	low_risk	immunogenic	Exocrine_like	Basel_like	0.217226	-0.16967	Epithelial
TCGA.IB.7890.01A	writer_cluster_2	DEG_cluster_B	0.250258668	high_risk	squamous	QM_PDA	Basel_like	0.107733	0.487585	Mesenchymal
TCGA.IB.7891.01A	writer_cluster_2	DEG_cluster_B	0.168868257	high_risk	immunogenic	Exocrine_like	Classical_M	-0.22749	0.445799	Mesenchymal
TCGA.IB.7893.01A	writer_cluster_2	DEG_cluster_B	0.309570203	high_risk	squamous	QM_PDA	Basel_like	-0.03762	0.495401	Mesenchymal
TCGA.IB.7897.01A	writer_cluster_2	DEG_cluster_B	-0.047521232	low_risk	immunogenic	QM_PDA	Basel_like	-0.26345	0.344874	Mesenchymal

TCGA.IB.8126.01A	writer_cluster_2	DEG_cluster_B	-0.105390652	low_risk	ADEX	Exocrine_like	Classical_M	-0.05664	-0.24788	Epithelial
TCGA.IB.8127.01A	writer_cluster_2	DEG_cluster_B	0.411408952	high_risk	immunogenic	Exocrine_like	Basel_like	0.421633	0.367827	Epithelial
TCGA.IB.A5SO.01A	writer_cluster_2	DEG_cluster_B	0.055557458	high_risk	immunogenic	Classical_C	Classical_M	0.185197	0.384043	Mesenchymal
TCGA.IB.A5SP.01A	writer_cluster_1	DEG_cluster_A	-0.162938496	low_risk	progenitor	Exocrine_like	Classical_M	0.419498	-0.49996	Epithelial
TCGA.IB.A5SQ.01A	writer_cluster_2	DEG_cluster_B	-0.075446625	low_risk	immunogenic	QM_PDA	Basel_like	0.086592	0.423721	Mesenchymal
TCGA.IB.A5SS.01A	writer_cluster_2	DEG_cluster_A	0.70717065	high_risk	squamous	QM_PDA	Basel_like	0.287846	0.484897	Mesenchymal
TCGA.IB.A5ST.01A	writer_cluster_2	DEG_cluster_B	0.025436554	high_risk	immunogenic	QM_PDA	Classical_M	-0.20464	0.412846	Mesenchymal
TCGA.IB.A6UF.01A	writer_cluster_1	DEG_cluster_A	-0.148435104	low_risk	ADEX	Exocrine_like	Basel_like	0.196175	-0.22665	Epithelial
TCGA.IB.A6UG.01A	writer_cluster_1	DEG_cluster_A	0.226550831	high_risk	ADEX	Exocrine_like	Basel_like	0.101269	-0.49261	Epithelial
TCGA.IB.A7LX.01A	writer_cluster_1	DEG_cluster_A	0.502644612	high_risk	squamous	QM_PDA	Basel_like	0.263016	-0.44752	Epithelial
TCGA.IB.A7M4.01A	writer_cluster_1	DEG_cluster_A	0.213634224	high_risk	squamous	Classical_C	Basel_like	0.422522	-0.28009	Epithelial
TCGA.IB.AAUM.01A	writer_cluster_1	DEG_cluster_A	-0.185626285	low_risk	ADEX	Exocrine_like	Classical_M	-0.23326	-0.4531	Epithelial
TCGA.IB.AAUN.01A	writer_cluster_2	DEG_cluster_B	0.482836369	high_risk	immunogenic	Exocrine_like	Basel_like	0.273972	-0.04567	Epithelial
TCGA.IB.AAUO.01A	writer_cluster_1	DEG_cluster_A	0.377723688	high_risk	squamous	QM_PDA	Basel_like	0.278262	-0.09519	Epithelial
TCGA.IB.AAUP.01A	writer_cluster_2	DEG_cluster_B	0.217095284	high_risk	immunogenic	QM_PDA	Basel_like	-0.32376	0.441032	Mesenchymal
TCGA.IB.AAUQ.01A	writer_cluster_1	DEG_cluster_B	-0.212191177	low_risk	squamous	QM_PDA	Basel_like	0.255985	0.297596	Epithelial

TCGA.IB.AAUR.01A	writer_cluster_1	DEG_cluster_B	-0.225855572	low_risk	immunogenic	QM_PDA	Basel_like	-0.24529	0.283317	Mesenchymal
TCGA.IB.AAUS.01A	writer_cluster_2	DEG_cluster_B	0.172408395	high_risk	immunogenic	Exocrine_like	Basel_like	-0.24099	0.460071	Mesenchymal
TCGA.IB.AAUT.01A	writer_cluster_1	DEG_cluster_B	-0.257530789	low_risk	immunogenic	Exocrine_like	Classical_M	-0.13043	0.26637	Mesenchymal
TCGA.IB.AAUU.01A	writer_cluster_2	DEG_cluster_A	0.097222021	high_risk	progenitor	Exocrine_like	Classical_M	0.476103	-0.32284	Epithelial
TCGA.IB.AAUV.01A	writer_cluster_2	DEG_cluster_B	-0.191970468	low_risk	immunogenic	Exocrine_like	Basel_like	-0.21017	0.48942	Mesenchymal
TCGA.IB.AAUW.01A	writer_cluster_2	DEG_cluster_B	-0.205565544	low_risk	immunogenic	Exocrine_like	Classical_M	-0.02505	-0.21481	Epithelial
TCGA.L1.A7W4.01A	writer_cluster_1	DEG_cluster_A	-0.318573551	low_risk	squamous	QM_PDA	Basel_like	0.354556	-0.0787	Epithelial
TCGA.LB.A7SX.01A	writer_cluster_1	DEG_cluster_A	0.042656907	high_risk	progenitor	Classical_C	Basel_like	0.114204	-0.3867	Epithelial
TCGA.LB.A8F3.01A	writer_cluster_1	DEG_cluster_A	-1.073128966	low_risk	ADEX	Exocrine_like	Classical_M	0.185923	-0.46505	Epithelial
TCGA.LB.A9Q5.01A	writer_cluster_2	DEG_cluster_A	-0.08621539	low_risk	ADEX	Exocrine_like	Basel_like	-0.22223	-0.25497	Epithelial
TCGA.M8.A5N4.01A	writer_cluster_1	DEG_cluster_A	0.500460038	high_risk	squamous	QM_PDA	Basel_like	0.364935	0.212924	Epithelial
TCGA.OE.A75W.01A	writer_cluster_1	DEG_cluster_A	0.319941712	high_risk	progenitor	Classical_C	Classical_M	0.285501	-0.11166	Epithelial
TCGA.PZ.A5RE.01A	writer_cluster_1	DEG_cluster_A	0.36743783	high_risk	squamous	Classical_C	Classical_M	0.438094	0.074361	Epithelial
TCGA.Q3.A5QY.01A	writer_cluster_1	DEG_cluster_B	0.178153707	high_risk	immunogenic	QM_PDA	Basel_like	-0.39456	0.036655	Mesenchymal
TCGA.Q3.AA2A.01A	writer_cluster_1	DEG_cluster_A	0.253563024	high_risk	progenitor	Classical_C	Classical_M	0.369682	-0.45852	Epithelial
TCGA.RB.A7B8.01A	writer_cluster_1	DEG_cluster_B	0.151145351	high_risk	squamous	Classical_C	Classical_M	0.019079	0.406714	Mesenchymal

TCGA.RB.AA9M.01A	writer_cluster_2	DEG_cluster_A	-0.133875239	low_risk	ADEX	QM_PDA	Basel_like	0.337797	-0.04257	Epithelial
TCGA.RL.AAAS.01A	writer_cluster_2	DEG_cluster_B	-0.157764769	low_risk	immunogenic	Exocrine_like	Classical_M	-0.15011	0.364422	Mesenchymal
TCGA.S4.A8RM.01A	writer_cluster_2	DEG_cluster_A	-0.7152017	low_risk	progenitor	Classical_C	Classical_M	0.216832	-0.47006	Epithelial
TCGA.S4.A8RO.01A	writer_cluster_1	DEG_cluster_A	-0.53743379	low_risk	progenitor	QM_PDA	Basel_like	0.296118	-0.42424	Epithelial
TCGA.S4.A8RP.01A	writer_cluster_1	DEG_cluster_B	0.24146707	high_risk	progenitor	Exocrine_like	Classical_M	0.321482	0.190382	Epithelial
TCGA.US.A774.01A	writer_cluster_2	DEG_cluster_B	0.107472309	high_risk	immunogenic	Exocrine_like	Basel_like	0.03863	0.473596	Mesenchymal
TCGA.US.A776.01A	writer_cluster_1	DEG_cluster_A	-1.218076515	low_risk	progenitor	Classical_C	Classical_M	0.313588	-0.48525	Epithelial
TCGA.US.A779.01A	writer_cluster_1	DEG_cluster_A	-0.528834983	low_risk	progenitor	Classical_C	Classical_M	0.279561	-0.49707	Epithelial
TCGA.US.A77E.01A	writer_cluster_1	DEG_cluster_A	-0.088454464	low_risk	progenitor	Classical_C	Classical_M	0.258102	0.377221	Mesenchymal
TCGA.US.A77G.01A	writer_cluster_2	DEG_cluster_A	-0.501862601	low_risk	progenitor	Exocrine_like	Classical_M	0.333318	-0.50403	Epithelial
TCGA.US.A77J.01A	writer_cluster_1	DEG_cluster_B	-0.393505464	low_risk	immunogenic	QM_PDA	Basel_like	-0.08083	0.152253	Mesenchymal
TCGA.XD.AAUG.01A	writer_cluster_2	DEG_cluster_B	-0.015575377	low_risk	immunogenic	Exocrine_like	Classical_M	-0.03882	0.436407	Mesenchymal
TCGA.XD.AAUH.01A	writer_cluster_2	DEG_cluster_B	-0.718427267	low_risk	immunogenic	QM_PDA	Basel_like	-0.4907	0.265275	Mesenchymal
TCGA.XD.AAUI.01A	writer_cluster_1	DEG_cluster_B	-0.420426721	low_risk	immunogenic	Exocrine_like	Basel_like	0.311075	0.103236	Epithelial

TCGA.XD.AAUL.01A	writer_cluster_1	DEG_cluster_A	-0.205914405	low_risk	squamous	Exocrine_like	Basel_like	0.420884	0.456192	Epithelial
TCGA.XN.A8T3.01A	writer_cluster_1	DEG_cluster_A	0.136213781	high_risk	squamous	Exocrine_like	Basel_like	0.158615	0.394549	Mesenchymal
TCGA.XN.A8T5.01A	writer_cluster_2	DEG_cluster_B	-0.018583139	low_risk	immunogenic	QM_PDA	Basel_like	-0.28992	0.354199	Mesenchymal
TCGA.YB.A89D.01A	writer_cluster_2	DEG_cluster_B	0.155212469	high_risk	squamous	Classical_C	Classical_M	0.237813	0.466762	Mesenchymal
TCGA.YH.A8SY.01A	writer_cluster_1	DEG_cluster_B	0.255363357	high_risk	squamous	QM_PDA	Basel_like	-0.13543	0.472999	Mesenchymal
TCGA.YY.A8LH.01A	writer_cluster_1	DEG_cluster_A	0.03959132	high_risk	progenitor	Classical_C	Classical_M	0.467244	-0.49202	Epithelial
TCGA.Z5.AAPL.01A	writer_cluster_1	DEG_cluster_B	-0.087613694	low_risk	immunogenic	QM_PDA	Classical_M	-0.30785	-0.27542	Epithelial

Supplementary Table 4: Differentially Expressed Genes between the two Writers_clusters and Univariate Cox Analysis for DEGs.

DEG_symbol	logFC	AveExpr	adj.P.Val	cox_HR	Upper_95_CI	Lower_95_CI	cox_p.val
SNORD99	-1.353492	3.9556694	1.04E-13	0.982	0.788	1.22	0.871
BRICD5	-1.0128629	2.6151614	3.05E-13	0.605	0.449	0.816	0.000974
RHPN1	-1.2528236	4.1880255	1.87E-12	0.805	0.665	0.973	0.0253
CPED1	1.19007531	2.8059846	2.00E-11	1.1	0.862	1.39	0.454
HSF4	-1.1123783	3.3232625	2.29E-11	0.79	0.63	0.99	0.0407
LMNTD2	-1.0258132	2.6541999	3.94E-11	0.876	0.656	1.17	0.37
HMCN1	1.13472816	2.4388436	6.33E-11	1.2	0.902	1.61	0.208
PCDH18	1.03376363	3.0838761	6.33E-11	1.11	0.85	1.46	0.433
FGF10	1.00653378	1.3490094	7.23E-11	1.26	0.877	1.81	0.211
KIFC2	-1.099162	3.8097451	9.42E-11	0.838	0.675	1.04	0.11
SNORD104	-1.3275257	5.2218192	1.01E-10	0.83	0.711	0.97	0.019
DDR2	1.20105764	3.9191975	1.84E-10	1.18	0.949	1.48	0.135
LAMA2	1.28996609	4.1398861	3.54E-10	1.17	0.968	1.4	0.106
RASSF7	-1.0705193	6.5385758	3.83E-10	1.01	0.836	1.22	0.926
FBN1	1.42590768	5.5174122	4.30E-10	1.19	1.01	1.41	0.0412
SVEP1	1.16585282	2.9800631	4.85E-10	1.07	0.849	1.34	0.584
SLIT2	1.1135763	3.0507221	5.22E-10	0.974	0.767	1.24	0.833
HEG1	1.00453845	4.7601194	5.43E-10	1.06	0.839	1.35	0.61
PABPC1L	-1.0006983	3.7415281	5.56E-10	0.778	0.62	0.978	0.0313
CPA3	1.40317457	4.7282323	6.01E-10	1.06	0.9	1.25	0.48
MZT2B	-1.0538155	6.2442044	8.81E-10	0.826	0.702	0.972	0.021
NIBAN1	1.00111237	3.9177947	1.04E-09	1.07	0.859	1.33	0.548
NOXA1	-1.2361169	5.0167854	1.10E-09	0.875	0.743	1.03	0.113
NEXN	1.18683501	3.7766123	1.27E-09	1.07	0.867	1.32	0.538
KIT	1.01920897	3.1405385	1.60E-09	0.986	0.775	1.25	0.908
PRR7	-1.001979	2.789243	1.87E-09	0.688	0.536	0.884	0.0034
OMD	1.43180629	3.187445	2.34E-09	1.08	0.91	1.28	0.385
PLXDC2	1.12553221	4.8003273	2.57E-09	1.16	0.94	1.42	0.169
SPON1	1.42419872	5.48991	2.72E-09	1.12	0.958	1.31	0.155
EVC	1.01827556	3.4124393	3.07E-09	1.22	0.947	1.59	0.123
C4orf48	-1.5657834	4.7507874	3.19E-09	0.869	0.761	0.993	0.0389

SELENOP	1.05301199	4.762727	4.52E-09	0.879	0.719	1.07	0.208
SNORD14A	-1.0348828	3.974815	4.57E-09	0.826	0.662	1.03	0.0894
SCX	-1.177781	2.6177573	6.56E-09	0.847	0.662	1.08	0.184
HES4	-1.0330392	4.4753312	8.27E-09	1.01	0.829	1.24	0.898
PDGFRA	1.12184675	4.560883	9.11E-09	1.14	0.939	1.39	0.181
ABI3BP	1.13573833	3.2853492	9.41E-09	1.06	0.863	1.3	0.592
CCDC85B	-1.0052793	5.2617794	1.24E-08	0.867	0.717	1.05	0.141
MSRB3	1.07680932	4.5690722	1.49E-08	1.13	0.915	1.39	0.257
EFEMP1	1.2452956	6.145482	1.60E-08	1.13	0.959	1.32	0.148
FGL2	1.16845657	4.2981677	1.95E-08	1.01	0.849	1.21	0.874
CYBRD1	1.10895674	6.4554951	2.05E-08	1.16	0.964	1.4	0.116
ITGBL1	1.32881044	4.0563783	3.11E-08	1.17	0.988	1.38	0.0691
ABCA8	1.00569482	2.0368385	3.58E-08	0.804	0.606	1.07	0.13
DCN	1.21104344	7.1874404	3.75E-08	1.14	0.967	1.34	0.121
SEMA3C	1.24995718	5.1472829	3.78E-08	1.42	1.18	1.7	0.000166
CYP1B1	1.36026724	4.6902074	3.83E-08	1.05	0.91	1.22	0.485
CCDC80	1.35998603	5.8525195	3.90E-08	1.11	0.966	1.28	0.137
OGN	1.57072695	3.5255369	4.01E-08	0.989	0.86	1.14	0.879
F13A1	1.33766908	5.0175967	4.80E-08	1.14	0.98	1.34	0.0873
COL14A1	1.31445756	5.2034237	5.04E-08	0.979	0.844	1.14	0.783
MIF	-1.21225	6.2979405	5.45E-08	0.923	0.799	1.07	0.277
IL1R1	1.03580476	5.8833298	5.59E-08	1.19	0.979	1.44	0.0805
CALD1	1.06449273	6.8608465	5.65E-08	1.16	0.957	1.4	0.13
F2R	1.00159092	6.1807468	5.97E-08	1.11	0.913	1.35	0.292
LTBP1	1.06438927	5.7522903	6.38E-08	1.33	1.08	1.64	0.00778
MOXD1	1.14495944	4.5162407	6.61E-08	1.1	0.922	1.31	0.291
CPXM2	1.0529057	4.4729729	1.05E-07	1.04	0.861	1.26	0.667
FGF7	1.13570665	2.6454998	1.14E-07	1.16	0.941	1.43	0.166
HSD11B1	1.09964324	4.0775776	1.15E-07	1.16	0.972	1.39	0.1
FPR3	1.16358293	4.1935543	1.21E-07	1.06	0.894	1.26	0.494
GPR34	1.04045243	3.2538893	1.41E-07	0.982	0.795	1.21	0.868
MSR1	1.05915502	3.7188262	1.50E-07	1.16	0.957	1.41	0.129
MYLK	1.09695437	3.9715562	1.90E-07	1.08	0.895	1.29	0.433

THBS1	1.36701212	7.3307215	2.27E-07	1.15	0.995	1.32	0.0583
PTPRC	1.23720326	3.9313577	2.76E-07	1	0.856	1.18	0.969
PLN	1.22867772	3.3470155	2.88E-07	1.05	0.88	1.24	0.613
MIR429	-1.2872671	4.2899662	3.12E-07	0.904	0.779	1.05	0.181
SYNPO2	1.13646233	3.1426325	3.15E-07	0.948	0.783	1.15	0.585
CRISPLD2	1.04339681	6.0709639	3.18E-07	1.11	0.928	1.33	0.256
MPEG1	1.10853956	4.1495946	3.30E-07	0.903	0.753	1.08	0.271
MNDA	1.03480093	3.7967843	4.50E-07	1.08	0.893	1.3	0.441
EPHA3	1.00964451	3.4315636	4.59E-07	1.01	0.822	1.23	0.959
ANGPTL1	1.05624851	2.6868088	4.60E-07	0.94	0.759	1.16	0.574
CHRDL1	1.47708135	3.3732149	5.89E-07	0.902	0.779	1.04	0.163
CD163	1.2345933	4.8740961	8.62E-07	1.12	0.96	1.3	0.154
AKAP12	1.01203867	5.0160121	8.64E-07	1.05	0.871	1.26	0.622
GPNMB	1.11986696	6.517478	1.00E-06	1.1	0.946	1.28	0.216
MIR200A	-1.1650705	2.6166055	1.01E-06	0.87	0.718	1.06	0.159
RSPO3	1.05353081	2.3189806	1.09E-06	0.983	0.785	1.23	0.879
CLMP	1.08421508	3.7231383	1.11E-06	1.23	1.02	1.5	0.0348
FPR1	1.00616064	3.9274288	1.18E-06	1.07	0.889	1.3	0.465
SLIT3	1.01342604	3.1976629	1.41E-06	0.886	0.72	1.09	0.256
LUM	1.1671797	9.7105597	1.52E-06	1.2	1.02	1.4	0.0252
CCN2	1.09098438	8.7195384	1.54E-06	1.03	0.89	1.2	0.667
C1S	1.00470466	7.8376177	1.67E-06	1.19	1	1.42	0.0474
CXCL12	1.10973217	4.7715639	1.89E-06	0.985	0.838	1.16	0.852
CDH11	1.05805712	4.7738067	1.99E-06	1.26	1.04	1.52	0.02
LOX	1.05972221	4.8767802	2.10E-06	1.33	1.09	1.61	0.00408
CTSG	1.04116823	1.9270462	2.51E-06	0.88	0.691	1.12	0.3
COL6A3	1.21920395	7.4054689	2.56E-06	1.24	1.06	1.44	0.00742
ANTXR1	1.10444363	6.3964289	2.66E-06	1.22	1.03	1.44	0.0233
COL8A1	1.23409388	5.1159826	2.97E-06	1.23	1.05	1.45	0.0102
HLA-DQA1	1.04887349	5.5080149	3.37E-06	1.1	0.937	1.28	0.25
MIR200B	-1.070113	2.0264706	3.54E-06	0.856	0.687	1.07	0.165
VCAN	1.17209629	6.3444245	3.65E-06	1.23	1.05	1.44	0.0112
MFAP4	1.08438099	7.1896444	3.92E-06	1.06	0.917	1.22	0.436

CYBB	1.02170192	4.8942023	4.31E-06	1.06	0.902	1.24	0.489
CLEC10A	1.0168399	2.7274905	4.32E-06	0.883	0.719	1.08	0.234
AOX1	1.02504771	3.0090996	4.56E-06	0.922	0.765	1.11	0.397
IL7R	1.15666535	3.8794872	5.34E-06	1	0.857	1.18	0.957
INHBA	1.15305678	5.0909555	6.16E-06	1.28	1.08	1.51	0.00424
FOLR2	1.00546139	4.6877371	6.64E-06	0.966	0.814	1.15	0.693
FCER1A	1.05143798	3.1286056	6.74E-06	0.942	0.781	1.14	0.529
MRC1	1.14189228	4.6161591	6.76E-06	0.956	0.821	1.11	0.569
MIR4768	-1.1850134	2.4824813	6.85E-06	1.11	0.931	1.32	0.249
COLEC12	1.04635216	3.3190682	7.20E-06	1.12	0.934	1.35	0.217
MIR210	-1.1315059	1.8864923	7.95E-06	1.04	0.87	1.25	0.648
FIBIN	1.0455886	4.5532609	1.10E-05	1.14	0.958	1.35	0.144
PTGIS	1.16463053	4.9307349	1.13E-05	1.13	0.985	1.3	0.0807
SFRP4	1.26273961	6.8392288	1.25E-05	1.05	0.932	1.17	0.45
THBS4	1.15387853	5.6539878	1.27E-05	0.986	0.865	1.12	0.832
IGFBP5	1.05176814	8.7010292	1.42E-05	1.04	0.909	1.2	0.552
FNDC1	1.18344706	4.8878684	1.60E-05	1.13	0.976	1.3	0.103
CILP	1.3075583	3.6607629	1.61E-05	1.06	0.925	1.22	0.399
COL5A2	1.07696573	7.4375446	1.70E-05	1.24	1.05	1.45	0.00924
ASPN	1.04354507	6.5110163	1.76E-05	1.16	0.988	1.36	0.0693
IGHV3-72	1.47104881	3.8331547	1.87E-05	0.992	0.885	1.11	0.895
GREM1	1.26227231	4.7804171	2.02E-05	1.2	1.04	1.37	0.0097
C7	1.37612022	6.6928021	2.39E-05	0.952	0.855	1.06	0.368
SCARA5	1.10291682	1.7237448	2.45E-05	0.906	0.741	1.11	0.336
DPT	1.15241494	5.112523	2.45E-05	1.02	0.893	1.17	0.741
COL3A1	1.16381105	10.847158	3.02E-05	1.19	1.04	1.37	0.0137
SULF1	1.12553372	6.7641664	3.26E-05	1.18	1.03	1.36	0.0196
IGHV3-66	1.23895065	2.9779653	3.75E-05	0.971	0.845	1.12	0.677
PGGHG	-1.0107862	5.513853	3.93E-05	1.16	1.01	1.34	0.0404
MMP2	1.09125603	8.3750726	4.42E-05	1.22	1.06	1.42	0.00718
THBS2	1.10559349	7.6587012	5.86E-05	1.21	1.05	1.4	0.0107
MFAP5	1.11077258	4.6975041	6.02E-05	1.18	1.02	1.36	0.0251
ADAM12	1.0148126	3.4211474	6.26E-05	1.16	0.973	1.39	0.0978

MATN3	1.11408228	4.7127237	6.28E-05	1.12	0.981	1.29	0.0918
IGHV1-18	1.68101861	6.1076378	7.26E-05	1.02	0.934	1.11	0.692
IGKV1D-8	1.02391879	2.0990325	7.56E-05	1	0.847	1.19	0.972
IGHV3-30	1.55789483	5.5182018	8.42E-05	1.02	0.93	1.12	0.688
IGHV3-20	1.04920099	2.2047276	9.36E-05	0.989	0.841	1.16	0.89
IGHV3-74	1.39467824	5.0563015	0.000106	1	0.903	1.11	0.991
ADH1B	1.1714726	3.4450596	0.000119	0.878	0.758	1.02	0.0807
IGHV2-5	1.28843288	3.1453684	0.000121	1.01	0.896	1.14	0.862
IGHV4-4	1.14870216	2.2081799	0.00013	1.05	0.911	1.21	0.5
SFRP2	1.27790835	9.4384836	0.000146	1.16	1.04	1.31	0.0107
IGHV3-15	1.46746412	5.9436312	0.000157	1.02	0.933	1.12	0.617
IGKV2-30	1.123712	2.7728194	0.000158	0.989	0.863	1.13	0.874
COL12A1	1.01818675	6.1454446	0.000184	1.24	1.07	1.45	0.00497
IGHV1-24	1.42200235	4.5609035	0.000202	1.04	0.94	1.15	0.454
IGHV3-21	1.386693	5.3119008	0.000204	1.03	0.928	1.13	0.617
IGHV3-73	1.31254613	3.7417561	0.000206	1.05	0.943	1.17	0.367
IGHV3-49	1.37714073	4.6736335	0.00021	0.967	0.874	1.07	0.511
IGHV2-26	1.39232384	3.7265699	0.00022	1.05	0.95	1.16	0.348
IGKV1-27	1.43271291	4.7581592	0.000221	1.04	0.948	1.15	0.374
IGHV2-70	1.30554611	3.166998	0.000241	0.999	0.888	1.12	0.985
MYH11	1.02938331	4.701062	0.000242	1.01	0.889	1.15	0.847
IGHV3-53	1.18932947	3.6353577	0.000247	1	0.887	1.14	0.942
IGHJ3	1.35390845	4.3100835	0.000257	0.986	0.884	1.1	0.799
IGHV3-64	1.14833949	2.0720694	0.000281	0.978	0.849	1.13	0.757
IGHV4-28	1.12908732	3.1632748	0.000301	1.03	0.901	1.17	0.7
IGHV1-46	1.310141	4.7529428	0.000304	1.02	0.92	1.12	0.745
IGHV4-59	1.39584305	5.5049954	0.000309	0.996	0.906	1.09	0.929
JCHAIN	1.40454368	7.2827503	0.000329	1	0.923	1.09	0.914
IGLV7-43	1.25763349	3.889658	0.00034	0.985	0.878	1.1	0.79
CXCL9	1.08665338	4.0645391	0.000341	1.18	1.05	1.31	0.00394
IGHV3-13	1.2653385	3.4090855	0.000472	1.03	0.927	1.15	0.549
COL10A1	1.24280084	6.4704861	0.0005	1.19	1.05	1.35	0.00492
IGHV4-61	1.09641796	3.0498946	0.000508	1	0.875	1.14	0.997

IGHV5-51	1.4475764	6.5880818	0.000522	1.02	0.936	1.11	0.675
IGHJ1	1.00027457	1.9037924	0.000527	0.972	0.826	1.14	0.736
IGLV3-21	1.43572396	6.1134329	0.000539	1.01	0.927	1.1	0.796
IGHV4-34	1.29524726	5.0841596	0.000556	1.02	0.923	1.13	0.673
IGKV4-1	1.39738574	7.2761332	0.000578	1.03	0.949	1.13	0.44
IGHV3-33	1.29931538	4.8130035	0.000594	1.01	0.916	1.12	0.808
IGKV1-5	1.37194514	7.0456344	0.000693	1.02	0.94	1.12	0.583
IGLV1-40	1.39092567	6.9063228	0.000732	1.04	0.954	1.13	0.38
IGLV1-44	1.35556118	5.9213436	0.000762	1.01	0.924	1.1	0.827
IGLV3-19	1.40169023	6.368446	0.000798	1.01	0.925	1.1	0.858
IGKV1-17	1.25659182	4.8884222	0.000879	1.04	0.941	1.15	0.438
IGHV4-39	1.38640409	6.4240637	0.000885	1.01	0.93	1.1	0.789
IGHV3-43	1.09042022	3.0224971	0.000954	1.02	0.896	1.15	0.804
IGHV3-11	1.30834402	5.0675563	0.000997	1.01	0.915	1.11	0.882
IGLV3-25	1.33334784	6.0143929	0.001027	0.987	0.905	1.08	0.772
IGHV4-30-2	1.23569175	4.2399042	0.001031	1.05	0.951	1.17	0.32
IGKV1-6	1.14041892	4.5128683	0.001033	0.999	0.896	1.11	0.986
IGHV1-69D	1.28096664	4.9350084	0.001068	1.02	0.924	1.13	0.691
IGHV1-69	1.30408527	3.5639526	0.001182	0.971	0.876	1.08	0.571
IGLV5-45	1.18757937	3.2296828	0.001225	1.01	0.9	1.13	0.87
IGHV3-23	1.28678337	6.9011615	0.001441	1.04	0.953	1.14	0.367
IGKJ5	1.03631672	2.6421012	0.001888	0.991	0.86	1.14	0.899
IGLV2-18	1.00342919	3.1125012	0.001915	0.971	0.857	1.1	0.647
IGLV2-23	1.20598166	6.4371128	0.002019	1.02	0.925	1.11	0.749
IGLV1-51	1.17539549	6.3505927	0.002415	1.01	0.921	1.1	0.863
IGLV6-57	1.16320647	4.9028462	0.002486	1.01	0.916	1.12	0.819
IGLV1-47	1.16593849	5.5964567	0.002492	1.03	0.939	1.14	0.499
IGLV3-9	1.11810536	3.543321	0.002718	1.02	0.918	1.14	0.7
IGKV3-20	1.26845412	7.8957657	0.002781	1.03	0.949	1.12	0.453
IGKV1-16	1.12357101	4.8959346	0.003001	0.978	0.885	1.08	0.665
IGKV3-15	1.11669302	5.8174528	0.003037	1.04	0.947	1.15	0.386
IGLV8-61	1.25314223	4.2394291	0.003077	0.998	0.906	1.1	0.966
IGLV2-8	1.08598873	4.9611321	0.003105	1	0.901	1.11	1

IGKV3D-20	1.01239797	3.5087814	0.003292	1.02	0.904	1.14	0.789
IGLV2-11	1.16514596	6.0778782	0.003338	1.01	0.926	1.11	0.762
IGKV1-9	1.13995169	5.1069695	0.003469	1.04	0.949	1.15	0.373
IGHV3-48	1.03978995	3.6745358	0.003669	1.03	0.916	1.16	0.629
IGKV2-24	1.04483782	4.2423475	0.003762	1.02	0.914	1.14	0.702
IGLV2-14	1.20659416	7.0831117	0.003793	0.995	0.913	1.08	0.902
PCSK1N	-1.0960933	5.3840881	0.005622	0.818	0.737	0.908	0.000156
CEACAM6	1.17982371	9.4455488	0.005668	1.19	1.07	1.33	0.00197
IGLV3-27	1.00383447	2.9054843	0.005713	1.02	0.91	1.14	0.754
PLA2G2A	1.26295395	4.2576789	0.008072	0.956	0.879	1.04	0.296
COL11A1	1.01819534	4.9790753	0.008494	1.16	1.04	1.3	0.00863
IGLV10-54	1.07017864	3.834294	0.010813	0.958	0.865	1.06	0.409
IGHV1-2	1.09848068	4.8807415	0.010908	1.02	0.933	1.12	0.662
IGHA1	1.05630011	10.418272	0.011479	1.02	0.943	1.11	0.593
IGLC2	1.00776133	8.4366136	0.011592	1.02	0.936	1.12	0.618
IGKV3-11	1.00728093	6.8931559	0.011937	1.01	0.921	1.11	0.823
IGLV3-10	1.0471257	4.5277953	0.012834	1.01	0.913	1.11	0.919
CXCL5	1.09488207	5.7828033	0.015406	1.12	1.03	1.22	0.00682
GABRP	1.043477	5.4050634	0.021047	1.14	1.04	1.25	0.00418

Supplementary Table 5: Samples clustering for WM_score validation in ICGC_AU_PAAD Cohort.

ICGC_ID	risk_score	risk_group
DO32829	-0.24846955	low
DO32860	-0.0283001	high
DO32863	-0.190574805	low
DO32875	0.023817975	high
DO32878	-0.141157507	low
DO32887	0.265433166	high
DO32900	0.013410217	high
DO32936	0.026735543	high
DO33091	-0.110367846	low
DO33128	-0.27156279	low
DO33168	0.366898288	high
DO33256	-0.035320104	high
DO33336	-0.093030017	low
DO33344	0.03516065	high
DO33368	-0.174257444	low
DO33376	-0.166213991	low
DO33392	-0.018524721	high
DO33400	-0.218955233	low
DO33408	-0.369049262	low
DO33472	-0.06158563	low
DO33480	-0.282758831	low
DO33512	-0.112790447	low
DO33528	-0.234839908	low
DO33544	-0.189942993	low
DO33600	-0.194791027	low
DO33632	-0.042486403	high
DO33656	-0.025095768	high
DO33984	-0.243777853	low
DO34240	0.021569431	high
DO34264	-0.214407926	low
DO34288	-0.01202278	high

DO34312	0.071972548	high
DO34336	-0.087088204	low
DO34376	0.093414388	high
DO34432	-0.138039527	low
DO34448	-0.224389864	low
DO34504	0.130491093	high
DO34600	-0.210575673	low
DO34608	-0.199105901	low
DO34640	0.020290247	high
DO34656	-0.409772114	low
DO34680	-0.070142342	low
DO34696	-0.17290272	low
DO34720	-0.016640011	high
DO34728	-0.073910655	low
DO34736	-0.232157305	low
DO34785	-0.125337426	low
DO34793	-0.094736512	low
DO34801	-0.159367195	low
DO34817	-0.247745448	low
DO34849	0.193675687	high
DO34905	-0.100111231	low
DO34945	-0.016511372	high
DO34961	-0.009063645	high
DO49074	0.364365606	high
DO49076	-0.282796023	low
DO49078	-0.212947998	low
DO49079	-0.033547433	high
DO49080	0.033669536	high
DO49087	0.05642924	high
DO49090	0.133406689	high
DO49105	0.138271514	high
DO49113	-0.213012804	low
DO49127	-0.028121161	high

DO49129	-0.241862776	low
DO49130	0.087215282	high
DO49133	-0.110771866	low
DO49135	-0.046775037	high
DO49138	0.078291974	high
DO49164	-0.128797514	low
DO49166	0.062449341	high
DO49168	-0.058291428	high
DO49170	0.018991033	high
DO49175	-0.038033841	high
DO49178	-0.195150285	low
DO49181	-0.239814724	low
DO49183	0.119535709	high
DO49184	0.09359497	high
DO49185	0.075446509	high
DO49193	-0.034211763	high
DO49198	0.301642817	high
DO49204	0.061596842	high

Supplementary Table 6: KEGG Enrichment Analysis for miRNA targeted DEGs.

KEGG_pathway_ID	Description	GeneRatio	BgRatio	pvalue	geneID	Count
hsa04974	Protein digestion and absorption	10/58	103/8105	2.22E-09	1301/1300/1290/1289/1278/1281/1293/1277/1295/1296	10
hsa04512	ECM-receptor interaction	8/58	88/8105	1.72E-07	3694/7058/2335/3673/1278/3914/1293/1277	8
hsa04510	Focal adhesion	9/58	201/8105	1.10E-05	4233/3694/7058/2335/3673/1278/3914/1293/1277	9
hsa04151	PI3K-Akt signaling pathway	11/58	354/8105	3.39E-05	4233/3694/7058/2335/3673/1278/3914/1293/1277/1969/2069	11
hsa05205	Proteoglycans in cancer	8/58	205/8105	9.45E-05	4233/2335/3673/5328/1278/1277/4313/7476	8
hsa05165	Human papillomavirus infection	10/58	331/8105	0.000102297	3694/7058/2335/3673/1278/3914/1293/1277/5743/7476	10
hsa04061	Viral protein interaction with cytokine and cytokine receptor	5/58	100/8105	0.000705962	53833/3627/6374/4283/9547	5
hsa04933	AGE-RAGE signaling pathway in diabetic complications	5/58	100/8105	0.000705962	2335/1278/1281/1277/4313	5
hsa05146	Amoebiasis	5/58	102/8105	0.000772572	2335/1278/3914/1281/1277	5

Supplementary Table 7: miRNA targeted DEGs and its Located Signalling Pathways between Different WM_score Subgroups.

miRNA	target_DEG	pathway
hsa-miR-3065-3p	COL11A1	Protein digestion and absorption
hsa-miR-7-5p	COL1A2	Protein digestion and absorption
hsa-miR-7-5p	COL1A2	ECM-receptor interaction
hsa-miR-7-5p	COL1A2	Focal adhesion
hsa-miR-7-5p	COL1A2	Epithelial-to-mesenchymal transition
hsa-miR-7-5p	COL1A2	PI3K-Akt signaling pathway
hsa-miR-7-5p	COL1A2	Proteoglycans in cancer
hsa-miR-7-5p	COL1A2	Human papillomavirus infection
hsa-miR-7-5p	COL1A2	Relaxin signaling pathway
hsa-miR-153-5p	COL1A2	Protein digestion and absorption
hsa-miR-153-5p	COL1A2	ECM-receptor interaction
hsa-miR-153-5p	COL1A2	Focal adhesion
hsa-miR-153-5p	COL1A2	Epithelial-to-mesenchymal transition
hsa-miR-153-5p	COL1A2	PI3K-Akt signaling pathway
hsa-miR-153-5p	COL1A2	Proteoglycans in cancer
hsa-miR-153-5p	COL1A2	Human papillomavirus infection
hsa-miR-153-5p	COL1A2	Relaxin signaling pathway
hsa-miR-153-5p	COL5A2	Protein digestion and absorption
hsa-miR-153-5p	FN1	ECM-receptor interaction
hsa-miR-153-5p	FN1	Focal adhesion
hsa-miR-153-5p	FN1	AGE-RAGE signaling pathway
hsa-miR-153-5p	FN1	PI3K-Akt signaling pathway
hsa-miR-153-5p	FN1	Proteoglycans in cancer
hsa-miR-153-5p	FN1	Human papillomavirus infection
hsa-miR-153-5p	FN1	Relaxin signaling pathway
hsa-miR-7-5p	COL8A1	Protein digestion and absorption
hsa-miR-135a-5p	COL5A1	Protein digestion and absorption
hsa-miR-3065-5p	THBS2	ECM-receptor interaction
hsa-miR-3065-5p	THBS2	Focal adhesion
hsa-miR-3065-5p	THBS2	AGE-RAGE signaling pathway
hsa-miR-3065-5p	THBS2	PI3K-Akt signaling pathway

hsa-miR-129-5p	COL1A1	Protein digestion and absorption
hsa-miR-129-5p	COL1A1	ECM-receptor interaction
hsa-miR-129-5p	COL1A1	Focal adhesion
hsa-miR-129-5p	COL1A1	Epithelial-to-mesenchymal transition
hsa-miR-129-5p	COL1A1	PI3K-Akt signaling pathway
hsa-miR-129-5p	COL1A1	Proteoglycans in cancer
hsa-miR-129-5p	COL1A1	Human papillomavirus infection
hsa-miR-129-5p	COL1A1	Relaxin signaling pathway
hsa-miR-153-5p	ITGB6	ECM-receptor interaction
hsa-miR-153-5p	ITGB6	Focal adhesion
hsa-miR-153-5p	ITGB6	AGE-RAGE signaling pathway
hsa-miR-153-5p	ITGB6	PI3K-Akt signaling pathway
hsa-miR-3065-5p	COL3A1	Protein digestion and absorption
hsa-miR-3065-5p	COL3A1	Human papillomavirus infection
hsa-miR-3065-5p	COL3A1	Relaxin signaling pathway
hsa-miR-7-2-3p	THBS2	ECM-receptor interaction
hsa-miR-7-2-3p	THBS2	Focal adhesion
hsa-miR-7-2-3p	THBS2	AGE-RAGE signaling pathway
hsa-miR-7-2-3p	THBS2	PI3K-Akt signaling pathway
hsa-miR-153-5p	LAMB3	ECM-receptor interaction
hsa-miR-153-5p	LAMB3	Focal adhesion
hsa-miR-153-5p	LAMB3	Epithelial-to-mesenchymal transition
hsa-miR-153-5p	LAMB3	Proteoglycans in cancer
hsa-miR-153-5p	LAMB3	Relaxin signaling pathway
hsa-miR-129-5p	COL5A1	Protein digestion and absorption
hsa-miR-153-5p	MET	Focal adhesion
hsa-miR-153-5p	MET	AGE-RAGE signaling pathway
hsa-miR-153-5p	MET	PI3K-Akt signaling pathway
hsa-miR-3065-5p	MET	Focal adhesion
hsa-miR-3065-5p	MET	AGE-RAGE signaling pathway
hsa-miR-3065-5p	MET	PI3K-Akt signaling pathway
hsa-miR-3065-5p	MMP2	PI3K-Akt signaling pathway
hsa-miR-3065-5p	MMP2	Human papillomavirus infection

hsa-miR-153-5p	COL8A1	Protein digestion and absorption
hsa-miR-7-2-3p	COL1A2	Protein digestion and absorption
hsa-miR-7-2-3p	COL1A2	ECM-receptor interaction
hsa-miR-7-2-3p	COL1A2	Focal adhesion
hsa-miR-7-2-3p	COL1A2	Epithelial-to-mesenchymal transition
hsa-miR-7-2-3p	COL1A2	PI3K-Akt signaling pathway
hsa-miR-7-2-3p	COL1A2	Proteoglycans in cancer
hsa-miR-7-2-3p	COL1A2	Human papillomavirus infection
hsa-miR-7-2-3p	COL1A2	Relaxin signaling pathway
hsa-miR-153-5p	COL5A1	Protein digestion and absorption
hsa-miR-153-3p	ITGB6	ECM-receptor interaction
hsa-miR-153-3p	ITGB6	Focal adhesion
hsa-miR-153-3p	ITGB6	AGE-RAGE signaling pathway
hsa-miR-153-3p	ITGB6	PI3K-Akt signaling pathway
hsa-miR-129-5p	COL10A1	Protein digestion and absorption
hsa-miR-7-2-3p	COL5A2	Protein digestion and absorption
hsa-miR-7-5p	COL5A2	Protein digestion and absorption
hsa-miR-129-5p	COL8A1	Protein digestion and absorption

Supplementary Table 8: Differential and Cox Analysis for PDUI (APA events) in TCGA_PAAD Cohort.

Gene_symbol	logFC	limma_p.value	HR	lower_95_CI	upper_95_CI	cox_p.value
COL1A2	0.06181818	7.63E-07	147	8.11	2680	0.00074
SDR16C5	-0.14375	0.001195502	0.15	0.0416	0.542	0.0038
KLK10	-0.1205682	0.013590135	0.107	0.0216	0.527	0.00604
DKK1	-0.2605682	2.24E-05	0.432	0.234	0.795	0.00698
S100A2	-0.1384091	0.004817054	0.183	0.0482	0.695	0.0126
TMPRSS4	-0.0651136	0.037815716	0.0513	0.00453	0.581	0.0165
LAMC2	-0.0946591	0.001029381	0.127	0.0218	0.737	0.0214
AREG	-0.1645455	2.67E-06	0.299	0.105	0.848	0.0233
CEACAM5	-0.1328409	9.03E-05	0.259	0.0773	0.87	0.0288
PLAUR	-0.1023864	0.000212531	0.182	0.0356	0.934	0.0412
ANXA10	-0.0856818	0.013806513	0.324	0.109	0.961	0.0422
BST2	0.025	0.03560755	10.1	1.07	95.5	0.0438

Supplementary Table 9: m6A targeting relationship between DEGs and m6A regulatory genes.

DEG_symbol	RBP	m6A_gene_type	tar_change
VCAN	ALKBH5	Erasers	UP
VCAN	ALKBH5	Erasers	UP
VCAN	ALKBH5	Erasers	UP
PCDH7	HNRNPC	Readers	UP
PCDH7	YTHDC1	Readers	UP
PCDH7	HNRNPC	Readers	UP
PCDH7	HNRNPC	Readers	UP
PCDH7	HNRNPC	Readers	UP
PCDH7	IGF2BP1	Readers	UP
PCDH7	IGF2BP2	Readers	UP
PCDH7	IGF2BP3	Readers	UP
PCDH7	YTHDF1	Readers	UP
PCDH7	YTHDF2	Readers	UP
PCDH7	YTHDF1	Readers	UP
PCDH7	YTHDF1	Readers	UP
PCDH7	YTHDF2	Readers	UP
PCDH7	YTHDF1	Readers	UP
PCDH7	YTHDF2	Readers	UP
PCDH7	YTHDF1	Readers	UP
PCDH7	YTHDF2	Readers	UP
PCDH7	YTHDF1	Readers	UP
PCDH7	YTHDF1	Readers	UP
AHNAK2	YTHDF2	Readers	UP
AHNAK2	YTHDF2	Readers	UP
DKK1	YTHDF2	Readers	UP
DKK1	YTHDF2	Readers	UP
DKK1	YTHDF2	Readers	UP
KIF1A	YTHDF1	Readers	DOWN
MET	IGF2BP1	Readers	UP
MET	IGF2BP3	Readers	UP
MET	HNRNPC	Readers	UP

MET	YTHDF2	Readers	UP
MET	IGF2BP1	Readers	UP
MET	IGF2BP3	Readers	UP
MET	YTHDF2	Readers	UP
MET	IGF2BP1	Readers	UP
MET	IGF2BP3	Readers	UP
MET	YTHDF2	Readers	UP
MET	IGF2BP3	Readers	UP
MET	YTHDF2	Readers	UP
MET	IGF2BP3	Readers	UP
MET	YTHDF2	Readers	UP
MET	IGF2BP3	Readers	UP
MET	YTHDF2	Readers	UP
MET	IGF2BP3	Readers	UP
MET	YTHDF2	Readers	UP
MET	IGF2BP3	Readers	UP
MET	YTHDF2	Readers	UP
MET	IGF2BP1	Readers	UP
MET	IGF2BP2	Readers	UP
MET	IGF2BP3	Readers	UP
MET	IGF2BP1	Readers	UP
MET	IGF2BP2	Readers	UP
MET	IGF2BP3	Readers	UP
MET	IGF2BP1	Readers	UP
MET	IGF2BP3	Readers	UP
MET	IGF2BP1	Readers	UP
MET	IGF2BP3	Readers	UP
MET	HNRNPC	Readers	UP
MET	IGF2BP1	Readers	UP
MET	YTHDF1	Readers	UP
MET	YTHDF2	Readers	UP
MET	HNRNPC	Readers	UP
MET	IGF2BP1	Readers	UP

MET	YTHDF1	Readers	UP
MET	YTHDF2	Readers	UP
MET	YTHDF2	Readers	UP
MET	YTHDF2	Readers	UP
MET	IGF2BP2	Readers	UP
MET	IGF2BP1	Readers	UP
MET	IGF2BP3	Readers	UP
MET	IGF2BP2	Readers	UP
MET	HNRNPC	Readers	UP
MET	YTHDF1	Readers	UP
MET	IGF2BP1	Readers	UP
MET	IGF2BP2	Readers	UP
MET	IGF2BP3	Readers	UP
MET	YTHDF3	Readers	UP
MET	IGF2BP1	Readers	UP
MET	IGF2BP2	Readers	UP
MET	IGF2BP3	Readers	UP
MET	YTHDF3	Readers	UP
MET	YTHDF1	Readers	UP
MET	YTHDF2	Readers	UP
SFN	YTHDF2	Readers	UP
VCAN	IGF2BP3	Readers	UP
VCAN	IGF2BP3	Readers	UP
VCAN	YTHDC1	Readers	UP
VCAN	YTHDC1	Readers	UP
VCAN	HNRNPC	Readers	UP
VCAN	YTHDF1	Readers	UP
VCAN	YTHDF3	Readers	UP
VCAN	YTHDF1	Readers	UP
VCAN	YTHDF1	Readers	UP
VCAN	YTHDF1	Readers	UP
VCAN	YTHDF1	Readers	UP
VCAN	YTHDF1	Readers	UP

VCAN	YTHDF1	Readers	UP
VCAN	YTHDC1	Readers	UP
VCAN	IGF2BP1	Readers	UP
VCAN	IGF2BP2	Readers	UP
VCAN	IGF2BP3	Readers	UP
VCAN	YTHDC1	Readers	UP
VCAN	YTHDC1	Readers	UP
VCAN	IGF2BP1	Readers	UP
VCAN	IGF2BP2	Readers	UP
VCAN	IGF2BP3	Readers	UP
VCAN	IGF2BP1	Readers	UP
VCAN	IGF2BP2	Readers	UP
VCAN	IGF2BP3	Readers	UP
VCAN	IGF2BP2	Readers	UP
VCAN	IGF2BP2	Readers	UP
VCAN	IGF2BP2	Readers	UP
VCAN	IGF2BP2	Readers	UP
VCAN	YTHDF1	Readers	UP
VCAN	IGF2BP2	Readers	UP
VCAN	YTHDF1	Readers	UP
VCAN	HNRNPC	Readers	UP
VCAN	HNRNPC	Readers	UP
VCAN	IGF2BP2	Readers	UP
VCAN	IGF2BP2	Readers	UP
VCAN	IGF2BP1	Readers	UP
VCAN	IGF2BP3	Readers	UP
VCAN	IGF2BP1	Readers	UP
VCAN	IGF2BP3	Readers	UP
VCAN	HNRNPC	Readers	UP
VCAN	YTHDF1	Readers	UP
VCAN	YTHDF2	Readers	UP
VCAN	YTHDF1	Readers	UP
VCAN	YTHDF2	Readers	UP

VCAN	YTHDF1	Readers	UP
VCAN	YTHDF2	Readers	UP
VCAN	IGF2BP2	Readers	UP
VCAN	HNRNPC	Readers	UP
VCAN	YTHDF1	Readers	UP
VCAN	HNRNPC	Readers	UP
VCAN	YTHDC1	Readers	UP
VCAN	YTHDC1	Readers	UP
VCAN	YTHDC1	Readers	UP
VCAN	IGF2BP3	Readers	UP
VCAN	IGF2BP3	Readers	UP
VCAN	IGF2BP1	Readers	UP
VCAN	IGF2BP2	Readers	UP
VCAN	IGF2BP3	Readers	UP
VCAN	IGF2BP1	Readers	UP
VCAN	IGF2BP2	Readers	UP
VCAN	IGF2BP3	Readers	UP
VCAN	IGF2BP1	Readers	UP
VCAN	IGF2BP2	Readers	UP
VCAN	IGF2BP3	Readers	UP
VCAN	IGF2BP1	Readers	UP
VCAN	IGF2BP2	Readers	UP
VCAN	IGF2BP3	Readers	UP
VCAN	IGF2BP1	Readers	UP
VCAN	IGF2BP3	Readers	UP
SEMA3C	HNRNPC	Readers	UP
SEMA3C	HNRNPC	Readers	UP
VGF	YTHDF2	Readers	DOWN
GPRC5A	YTHDF2	Readers	UP
GPRC5A	YTHDF2	Readers	UP
GPRC5A	YTHDF2	Readers	UP
GPRC5A	YTHDF2	Readers	UP
GPRC5A	YTHDF2	Readers	UP

GPRC5A	YTHDF2	Readers	UP
GPRC5A	YTHDF2	Readers	UP
GPRC5A	YTHDF2	Readers	UP
GPRC5A	YTHDF2	Readers	UP
GPRC5A	YTHDF2	Readers	UP
GPRC5A	YTHDF2	Readers	UP
GPRC5A	YTHDF2	Readers	UP
GPRC5A	YTHDF2	Readers	UP
GPRC5A	HNRNPC	Readers	UP
GPRC5A	IGF2BP3	Readers	UP
FN1	YTHDF1	Readers	UP
FN1	YTHDF1	Readers	UP
FN1	IGF2BP1	Readers	UP
FN1	IGF2BP1	Readers	UP
SLC2A1	IGF2BP1	Readers	UP
SLC2A1	YTHDF2	Readers	UP
SLC2A1	IGF2BP1	Readers	UP
SLC2A1	IGF2BP2	Readers	UP
SLC2A1	IGF2BP3	Readers	UP
SLC2A1	YTHDF1	Readers	UP
SLC2A1	YTHDF2	Readers	UP
SLC2A1	IGF2BP1	Readers	UP
SLC2A1	IGF2BP2	Readers	UP
SLC2A1	IGF2BP3	Readers	UP
SLC2A1	YTHDF1	Readers	UP
SLC2A1	YTHDF2	Readers	UP
SLC2A1	IGF2BP1	Readers	UP
SLC2A1	YTHDF1	Readers	UP
SLC2A1	YTHDF2	Readers	UP
SLC2A1	IGF2BP1	Readers	UP
SLC2A1	YTHDF1	Readers	UP
SLC2A1	YTHDF2	Readers	UP
SLC2A1	IGF2BP1	Readers	UP

SLC2A1	YTHDF1	Readers	UP
SLC2A1	YTHDF2	Readers	UP
SLC2A1	IGF2BP1	Readers	UP
SLC2A1	YTHDF1	Readers	UP
SLC2A1	YTHDF2	Readers	UP
SLC2A1	IGF2BP1	Readers	UP
SLC2A1	YTHDF1	Readers	UP
SLC2A1	IGF2BP1	Readers	UP
SLC2A1	YTHDF1	Readers	UP
SLC2A1	HNRNPC	Readers	UP
SLC2A1	IGF2BP1	Readers	UP
SLC2A1	IGF2BP2	Readers	UP
SLC2A1	IGF2BP3	Readers	UP
SLC2A1	YTHDF1	Readers	UP
SLC2A1	YTHDF2	Readers	UP
CA12	IGF2BP2	Readers	UP
CA12	IGF2BP3	Readers	UP
CA12	IGF2BP3	Readers	UP
CA12	IGF2BP3	Readers	UP
CA12	YTHDF3	Readers	UP
CA12	IGF2BP1	Readers	UP
CA12	IGF2BP2	Readers	UP
CA12	YTHDF3	Readers	UP
ANTXR1	YTHDF1	Readers	UP
ANTXR1	YTHDF2	Readers	UP
ANTXR1	YTHDF2	Readers	UP
SLC13A5	IGF2BP1	Readers	UP
SLC13A5	IGF2BP3	Readers	UP
CEMIP	IGF2BP1	Readers	UP
CEMIP	IGF2BP1	Readers	UP
COL5A1	YTHDF2	Readers	UP
COL12A1	HNRNPC	Readers	UP
PITX1	YTHDF1	Readers	UP

PITX1	YTHDF2	Readers	UP
PITX1	YTHDF1	Readers	UP
PITX1	YTHDF2	Readers	UP
PITX1	YTHDF1	Readers	UP
PITX1	YTHDF2	Readers	UP
PITX1	YTHDF1	Readers	UP
PITX1	YTHDF2	Readers	UP
PITX1	YTHDF2	Readers	UP
PITX1	IGF2BP2	Readers	UP
PITX1	YTHDC1	Readers	UP
PITX1	YTHDF1	Readers	UP
PITX1	YTHDF2	Readers	UP
PITX1	IGF2BP2	Readers	UP
PITX1	YTHDC1	Readers	UP
PITX1	YTHDF1	Readers	UP
PITX1	YTHDF2	Readers	UP
PITX1	IGF2BP2	Readers	UP
PITX1	YTHDC1	Readers	UP
PITX1	YTHDF1	Readers	UP
PITX1	YTHDF2	Readers	UP
PITX1	IGF2BP2	Readers	UP
PITX1	YTHDC1	Readers	UP
PITX1	YTHDF1	Readers	UP
PITX1	YTHDF2	Readers	UP
PITX1	IGF2BP2	Readers	UP
PITX1	YTHDC1	Readers	UP
PITX1	YTHDF1	Readers	UP
PITX1	YTHDF2	Readers	UP
PITX1	IGF2BP2	Readers	UP
PITX1	YTHDC1	Readers	UP
PITX1	YTHDF1	Readers	UP
PITX1	YTHDF2	Readers	UP
PITX1	IGF2BP2	Readers	UP

PITX1	IGF2BP3	Readers	UP
PITX1	YTHDF1	Readers	UP
PITX1	YTHDF2	Readers	UP
PITX1	IGF2BP2	Readers	UP
PITX1	IGF2BP3	Readers	UP
PITX1	YTHDF1	Readers	UP
PITX1	YTHDF2	Readers	UP
PITX1	YTHDF2	Readers	UP
PITX1	YTHDC2	Readers	UP
PITX1	YTHDF2	Readers	UP
PITX1	YTHDC2	Readers	UP
PITX1	YTHDF2	Readers	UP
PCDH7	YTHDF1	Readers	UP
PCDH7	YTHDF1	Readers	UP
PCDH7	YTHDF2	Readers	UP
PCDH7	YTHDF1	Readers	UP
PCDH7	HNRNPC	Readers	UP
PCDH7	YTHDF1	Readers	UP
PCDH7	YTHDF2	Readers	UP
PCDH7	YTHDF1	Readers	UP
PCDH7	YTHDF1	Readers	UP
PCDH7	YTHDF2	Readers	UP
PCDH7	HNRNPC	Readers	UP
PCDH7	HNRNPC	Readers	UP
PCDH7	YTHDF1	Readers	UP
PCDH7	YTHDF2	Readers	UP
PRSS21	YTHDC2	Readers	UP
PRSS21	YTHDF2	Readers	UP
COL12A1	HNRNPC	Readers	UP
COL12A1	HNRNPC	Readers	UP
FN1	YTHDF1	Readers	UP
VCAN	HNRNPC	Readers	UP
VCAN	IGF2BP2	Readers	UP

VCAN	IGF2BP2	Readers	UP
VCAN	YTHDC1	Readers	UP
VCAN	IGF2BP1	Readers	UP
VCAN	IGF2BP3	Readers	UP
VCAN	YTHDC1	Readers	UP
VCAN	YTHDF1	Readers	UP
VCAN	YTHDF1	Readers	UP
VCAN	YTHDF1	Readers	UP
VCAN	YTHDF2	Readers	UP
VCAN	IGF2BP1	Readers	UP
VCAN	IGF2BP2	Readers	UP
VCAN	IGF2BP3	Readers	UP
VCAN	YTHDC1	Readers	UP
PITX1	YTHDF1	Readers	UP
PITX1	YTHDF2	Readers	UP
MET	HNRNPC	Readers	UP
MET	IGF2BP1	Readers	UP
MET	YTHDF1	Readers	UP
MET	YTHDF2	Readers	UP
MET	IGF2BP1	Readers	UP
MET	IGF2BP3	Readers	UP
MET	IGF2BP1	Readers	UP
MET	IGF2BP3	Readers	UP
MET	YTHDF2	Readers	UP
MET	IGF2BP1	Readers	UP
MET	IGF2BP2	Readers	UP
MET	IGF2BP3	Readers	UP
MET	YTHDF2	Readers	UP
MET	IGF2BP1	Readers	UP
MET	IGF2BP3	Readers	UP
MET	IGF2BP1	Readers	UP
MET	IGF2BP3	Readers	UP
MET	IGF2BP1	Readers	UP

MET	IGF2BP3	Readers	UP
GPRC5A	YTHDF2	Readers	UP
GPRC5A	YTHDF2	Readers	UP
GPRC5A	YTHDF2	Readers	UP
INSM1	IGF2BP1	Readers	DOWN
INSM1	IGF2BP3	Readers	DOWN
COL5A1	YTHDF2	Readers	UP
MYEOV	YTHDF2	Readers	UP
SFN	YTHDF2	Readers	UP
SFN	YTHDF2	Readers	UP
SFN	YTHDF2	Readers	UP
SFN	YTHDF2	Readers	UP
SFN	YTHDF2	Readers	UP
PRSS21	YTHDF2	Readers	UP
PRSS21	YTHDF2	Readers	UP
PRSS21	YTHDF2	Readers	UP
SEMA3C	YTHDF1	Readers	UP
VGF	YTHDF2	Readers	DOWN
VGF	YTHDF2	Readers	DOWN
IL20RB	HNRNPC	Readers	UP
IL20RB	HNRNPC	Readers	UP
COL5A1	YTHDF2	Readers	UP
COL5A1	YTHDF2	Readers	UP
COL5A1	YTHDF2	Readers	UP
COL5A1	YTHDF2	Readers	UP
COL5A1	YTHDF2	Readers	UP
SFN	YTHDF2	Readers	UP
SEMA3C	YTHDF1	Readers	UP
COL5A1	YTHDF2	Readers	UP
COL5A1	YTHDF2	Readers	UP
MET	RBM15	Writers	UP
VCAN	RBM15B	Writers	UP
VCAN	RBM15B	Writers	UP

RAB26	RBM15	Writers	DOWN
RAB26	RBM15	Writers	DOWN
SEMA3C	RBM15	Writers	UP
SEMA3C	RBM15	Writers	UP
SLC13A5	RBM15	Writers	UP
PRSS21	RBM15	Writers	UP
VCAN	RBM15B	Writers	UP
VCAN	RBM15B	Writers	UP
VCAN	RBM15B	Writers	UP
VCAN	RBM15B	Writers	UP

Supplementary Table 10: m1A targeting relationship between DEGs and m6A regulatory genes.

DEG_symbol	RBP	m1A_gene_type	tar_change
SFN	YTHDF2	Readers	UP
H4C8	YTHDC1	Readers	UP
H4C8	YTHDF1	Readers	UP
H4C8	YTHDC1	Readers	UP
H4C8	YTHDF1	Readers	UP
H4C8	YTHDF1	Readers	UP
H4C8	YTHDF1	Readers	UP
VGF	YTHDF2	Readers	DOWN
COL1A1	YTHDF2	Readers	UP
ANTXR1	YTHDF2	Readers	UP
ANTXR1	YTHDF2	Readers	UP
COL5A1	YTHDF2	Readers	UP
COL5A1	YTHDF2	Readers	UP
COL5A1	YTHDF2	Readers	UP
PITX1	YTHDF1	Readers	UP
PITX1	YTHDF2	Readers	UP
H4C8	YTHDF1	Readers	UP
H4C8	YTHDC1	Readers	UP
H4C8	YTHDF1	Readers	UP
KRT7	YTHDF2	Readers	UP
KRT7	YTHDF2	Readers	UP
COL5A1	YTHDF2	Readers	UP
COL5A1	YTHDF2	Readers	UP

Supplementary Table 11: IC50 for compounds and WM_score in PAAD cell lines.

PAAD cells / Compounds	AsPC-1	BxPC-3	CAPAN-1	CFPAC-1	DAN-G	HPAC	HPAF-II	Hs-766T	HuP-T3	HuP-T4	KP-1N	KP-2	KP-4	MIA-PaCa-2	PA-TU-8902	PA-TU-8988T	PANC-02-03	PANC-03-27	PANC-04-03	PANC-08-13	PANC-10-05	PL18	PL4	PSN1	SU8686	SUIT-2	SW1990	YAPC
risk_score	-0.234472155	0.216903	-0.29849	0.013644	-0.31554	0.685437	-0.21302	0.0060116	-0.13183	-0.45015	0.598122	-0.07917	0.166661	-0.49948	0.397096	-0.69267	0.092502	0.239194	0.486432	0.219977	0.01386	-0.54822	-0.74388	-0.85531	-0.29911	-0.30852	-0.39507	-0.16488
5-Fluorouracil	1.1079315	-1.365	0.0252238	-2.06519	-2.05101	1.711218	0.997478	1.534631	0.013253	-0.07252	-1.08119	0.963768	-1.36106	-0.86807	0.970846	-0.15429	-1.56969	-1.95873	0.677272	1.734909	0.787457	-1.32375	-0.35909	-3.79357	3.299826	0.132237	0.834208	-0.01325
ABT737	1.79272	0.051187	-0.62679	-0.12468	-0.98359	-0.42353	-2.5206	-0.71282	0.223434	0.711537	1.887004	-1.4472	0.799142	1.817366	-0.2086	-0.72979	0.518592	-0.05119	2.675161	0.053638	0.273547	-1.47625	-1.52602	-0.58537	-0.65757	0.894099	0.619422	1.131255
Acetalax	3.9359155	-2.92488	-1.75707	0.667958	-0.33185	1.006694	-0.389	-3.8639	-2.62424	-2.1477	0.331846	3.255408	0.772096	-0.98129	-1.5033	2.677235	2.61592	-3.8639	-3.8639	2.619327	2.45138	2.488623	-2.54286	2.401149	-0.80719	3.787657	-3.8639	0.940539
Afatinib	1.248125	-0.6645	-0.70803	-2.15485	-1.69768	0.318689	-0.12198	1.517098	0.093117	0.645024	-0.41508	0.028518	-0.34156	-0.26311	0.968506	-0.48315	-1.15362	-0.92356	0.720295	0.934336	-0.58728	1.369869	0.532896	-0.23942	1.896248	1.782942	1.092679	-0.02852
Afuresertib	1.70444	-0.53471	0.144336	-0.39288	-1.15717	0.439193	0.073001	-0.34779	-0.37857	0.005976	-0.42957	0.773321	-0.16215	-0.3189	-0.70933	-0.90458	-0.00598	0.441714	0.873376	0.833844	-0.88946	0.01774	-0.16359	-0.3382	1.279143	1.804756	0.292038	0.948809
AGI-5198	2.6289855	-0.69781	0.104384	-1.17604	-1.26723	0.407656	0.230404	0.629797	-0.06164	-1.16224	0.061638	0.386151	-1.78296	-0.54972	0.633041	-1.03877	-1.18715	0.332403	1.609766	0.153355	0.918928	-0.30899	-0.71893	-1.87553	0.206905	-0.51162	-0.68856	0.744773
AGI-6780	1.953055	-1.4	-0.8	0.250	-1.1	-0.9	0.454	-4.8	0.301	0.938	0.518	-0.1	-1.0	0.506	-0.0	-0.3	0.302	-4.8	-4.8	0.441	0.851	-2.0	0.821	0.015	2.123	-0.7	-4.8	0.223

		8913	926	613	6318	0481	007	0791	848	421	108	165	8459	747	1592	2186	002	0791	0791	684	837	0576	906	924	309	4649	0791	413
Alisertib	4.1685025	-1.25539	0.27194	0.28632	-1.29	-0.66854	2.4731116	-2.83512	-0.27194	-0.98126	1.345789	0.804025	-3.34673	-0.60459	0.825333	1.914637	-0.45593	-2.83512	-2.83512	-0.68376	1.340809	1.280455	-1.36358	0.354534	3.944404	3.181168	-2.83512	2.472233
Alpelisib	1.0129935	-1.61144	-0.25633	-0.38344	-2.57023	1.56501	0.914776	0.819576	-1.04672	-0.2336	0.787919	0.267427	0.66157	-1.11935	-1.09525	0.818912	-1.33069	-0.58174	1.325568	-0.41649	-0.02144	0.035803	-1.13436	0.021439	1.228689	2.533315	1.491955	-1.43448
AMG-319	1.254498	-0.73352	-0.12831	-0.93809	-2.39982	0.780787	0.130118	-0.12573	0.610355	-0.16602	0.040781	-0.0145	-1.76845	-1.94614	0.936912	0.13417	-0.45837	-0.86725	2.499114	0.42367	-0.34906	-0.7179	-0.9816	0.014497	1.431186	0.367057	0.024483	0.163157
AT13148	0.661981	-2.44161	-0.07362	0.353386	-2.0904	0.360879	0.775202	-1.30372	0.073623	-0.70687	-0.7118	-2.20242	-1.20806	-2.00671	-0.08425	-3.243	0.727683	0.178878	1.693297	0.828436	0.529846	-0.78252	-0.73937	-2.01955	1.720749	1.182867	1.131365	0.652443
AZ6102	0.6223265	-1.44773	-1.24893	-2.30048	-1.86982	-0.01831	1.709264	3.223634	0.563933	-1.02208	-0.90047	-1.3007	-0.77532	0.5184	0.743076	-1.08413	0.018314	-1.09457	1.007252	-0.08227	-1.43618	0.605295	-2.29749	0.426124	0.386936	1.308073	0.694927	1.007255
AZ960	3.9034585	-0.1707	2.201758	-1.27352	-1.7508	1.919175	1.05499	-3.24599	0.397227	-0.35244	-0.11326	2.292952	-2.17229	-1.10653	0.532675	0.18793	-0.8858	-3.24599	-3.24599	0.181245	0.11326	0.315344	-0.81307	-0.8972	1.973352	3.029075	-3.24599	1.548058
AZD1208	1.678206	0.169861	0.626414	0.526289	-0.54762	1.087033	0.424087	-5.92531	-0.77602	-0.1488	-0.21697	0.736424	-0.54709	-0.37781	0.872768	-0.29205	0.913061	-5.92531	-5.92531	0.201264	1.121064	0.148804	-0.17924	-0.53372	0.728401	0.94698	-5.92531	-0.42532
AZD1332	3.0065845	-0.25826	0.579725	-0.8729	-1.23004	0.835308	0.358767	-3.89689	-0.34454	0.170601	-0.15062	0.327078	1.390463	-0.48154	0.25875	-0.23307	0.150617	-3.89689	-3.89689	-1.25413	2.81658	0.397988	-1.38154	-0.99534	1.711944	2.938615	-3.89689	0.966384
AZD2014	1.571233	-0.66168	0.954093	0.742292	-2.3567	0.191238	-1.11172	-2.59973	1.789397	1.793195	0.450854	1.431743	-0.88193	0.561145	0.012441	-0.40811	-0.01244	-2.59973	-2.59973	-0.34946	-0.29893	-0.17964	-0.97899	0.182581	2.177892	1.347449	-2.59973	1.040018

AZD3759	1.141955	-1.69904	-0.70386	-0.59604	-1.09512	-0.27718	-0.09859	0.937666	0.449297	-0.09141	0.892308	0.927729	-0.71336	-0.47488	0.35766	0.792108	-0.56125	-0.80416	0.66259	-0.07752	0.730664	-0.00592	0.332226	-0.36479	0.005919	1.019013	0.874883	0.469089
AZD4547	2.617255	-0.5512	-1.22894	-1.43241	-0.91623	1.379807	0.256135	1.597756	-2.22433	-1.74557	-0.00218	0.142261	0.109886	-0.03802	-0.14947	-1.09675	0.24783	0.00176	2.299727	-0.5537	1.114154	0.580361	-1.34544	-0.14793	1.500071	-0.00018	0.979212	0.901183
AZD5153	4.060316	-0.12375	1.12503	-0.77769	0.498984	0.123746	1.686278	-1.90485	0.711361	1.472368	-0.18368	3.006264	-0.59864	-3.33549	2.226773	-1.78414	-1.60268	-1.90485	-1.90485	-2.07237	-0.5003	-2.4685	0.231194	2.047103	4.540256	0.467245	-1.90485	3.050293
AZD5363	1.0263745	-0.183	0.5032	-0.16713	-1.77624	0.239079	-0.31063	-0.52554	-0.1808	-0.30459	0.166306	1.611025	-1.32211	-0.227	-1.06555	-0.62805	0.868389	0.400695	0.508708	0.586431	0.036045	0.275548	-0.03605	-0.59223	1.145264	1.794989	-0.51531	0.588994
AZD5438	3.9719335	-1.41884	0.272437	-0.23143	-0.63533	0.651858	1.429527	-2.65206	2.196112	0.231431	1.022548	0.94887	-1.3852	-1.42378	1.682221	-0.95216	0.876965	-2.65206	-2.65206	-0.29414	2.788683	-0.79123	-0.99659	-1.07437	1.549534	2.285348	-2.65206	0.53309
AZD5582	2.63874	-3.44876	-0.72304	-1.79167	-1.31982	-0.05275	0.052752	-3.21231	1.42772	1.66263	1.339556	2.24273	-0.95085	-1.61986	0.85941	1.070019	0.148903	-3.21231	-3.21231	2.258652	-0.40451	1.973707	-0.24225	-0.88607	2.567827	3.806708	-3.21231	3.360964
AZD5991	2.612138	-1.57151	0.575648	0.414199	-0.57856	0.874229	0.235809	-6.09527	-1.25296	-1.90711	-0.17657	0.305512	-0.49469	-0.52959	0.804992	-0.21287	1.366925	-6.09527	-6.09527	0.133443	1.246169	0.232906	-0.13344	-1.08643	0.557994	0.808673	-6.09527	0.453851
AZD6738	1.972581	1.726973	-0.20754	-0.46644	-1.36843	-0.30449	-0.30418	-0.19138	-1.21043	-0.14031	1.14224	2.489904	-0.38314	0.543731	0.418013	1.140779	-0.83834	-0.83821	1.00859	1.451976	1.338582	1.631206	-1.81834	-0.1295	0.948987	-0.01752	0.017519	1.223032
AZD7762	1.9799	-1.34739	0.132101	-0.96128	-1.45716	-0.14264	0.198609	-1.22856	-1.05531	0.417314	0.575234	0.13961	-0.10653	-0.34032	1.019516	0.047829	-0.58597	-1.70918	1.321155	1.125486	0.484111	0.727617	-0.63871	-0.4593	1.836783	0.754996	-0.37558	-0.04783
AZD8186	2.4906565	-0.44898	0.246583	-1.31787	-0.90951	-0.30784	0.112869	-0.86461	-0.6489	0.038832	0.240758	0.692738	-0.03883	0.914392	-0.28358	-0.61311	-0.86152	-0.47084	0.352296	-0.26106	1.76777	0.78747	-1.2491	0.395718	-0.49281	1.667386	1.796535	0.689428

BDP-00009066	2.1023925	-1.08917	-0.82404	0.0005	-1.43078	1.392356	0.863516	-0.01598	-0.67764	-0.82781	0.025912	-0.6027	-0.49866	-0.53933	0.007745	-0.88732	-0.50558	2.054018	1.088922	0.078059	0.039413	-0.0005	-0.72255	-1.56669	0.984966	1.246151	1.737932	0.067557
BIBR-1532	0.4593685	-0.35564	0.342774	-0.47602	-1.37692	0.195221	0.486297	0.730301	-0.62648	-1.37641	-1.13747	-0.93647	-0.52282	-0.93743	0.676963	-0.15298	0.379621	0.304254	0.687352	0.083533	0.541426	0.017415	-1.29696	-0.83225	0.207079	-0.13218	-0.01741	0.630126
BMS-345541	2.3661745	-1.98172	0.594572	-1.25093	-1.50275	0.427645	1.708936	-4.15925	0.398937	0.758462	-0.5402	1.176506	-0.52247	-1.06903	1.061881	-0.09369	-1.32803	-4.15925	-4.15925	0.127723	0.261262	0.3423	-0.81133	-1.81329	2.691007	0.093687	-4.15925	0.413522
BMS-536924	1.72925	-0.31212	0.428108	-0.02216	-0.72797	-0.97897	0.218841	0.25561	-1.18142	0.403101	-0.39443	-0.88772	1.313521	-0.10457	0.022155	-0.23331	-0.29069	0.683198	1.480416	-0.09041	0.322283	1.113419	-1.15122	-0.33926	1.022288	1.779388	2.967201	-0.49588
Bortezomib	5.6762595	0.008422	-0.00842	-0.84917	-1.08218	-0.54662	-0.15298	0.69347	-0.74579	-0.1173	-0.84264	0.030729	0.167913	0.640891	-0.06121	-1.07993	-0.20801	1.627926	2.743126	-0.31595	0.175927	0.418771	0.088191	-0.43596	1.442376	0.998535	-0.94407	0.289051
BPD-00008900	1.136655	-0.25193	-1.22371	0.177187	-0.60496	1.927404	0.658405	0.623405	-0.45888	-1.00848	-0.17719	-0.44046	-1.31704	-1.04479	1.218347	-0.77948	-0.30959	0.664536	0.609218	0.1871	-1.68221	-0.95561	0.476825	-2.46604	1.530709	0.932788	0.34542	0.235358
Buparlisib	2.4306275	-0.37313	-0.30164	-0.29665	-1.27148	0.417741	-0.03959	0.382034	0.37339	-0.14964	-0.12745	0.039589	-0.77668	0.126989	-0.53926	-0.69402	0.243158	-0.23314	1.553244	0.329806	0.41716	-0.2482	-1.16307	-0.11152	1.180585	0.772035	1.179027	0.536394
Camptothecin	4.165385	0.255842	-1.95191	-1.77747	-0.29554	2.62756	1.412163	3.252934	0.74843	-1.67019	-0.37388	2.347848	-1.58037	-0.99146	0.795245	0.571801	-0.65146	-0.03376	1.619833	3.938833	0.033764	-1.30118	-0.89627	-1.42393	4.144402	-0.54332	0.897325	-0.04747
Carmustine	2.330619	-0.24297	0.057412	-0.42925	-0.48926	0.421867	0.392172	-6.81295	0.623327	-1.11914	-0.22383	-0.03883	-0.31831	-0.4882	1.128621	0.038827	0.376577	-6.81295	-6.81295	0.716487	-0.15927	0.091669	-0.98224	0.100496	0.352691	1.172597	-6.81295	0.299302
CDK9_5038	3.7619885	-1.71053	-0.755	-0.6392	-1.52521	-0.0391	-0.67872	0.665019	1.039139	0.105828	-0.16886	0.471367	-2.31097	-2.68634	0.985384	-2.32946	0.039098	0.665019	0.665019	0.080788	-0.24655	-1.5778	-1.16494	-2.36172	1.849403	2.041815	0.665019	1.71361

CDK9_5576	3.50903	-1.10963	-0.05699	-0.13295	-0.29699	0.450527	0.804374	0	1.66042	-0.39022	0.662532	0.803058	-0.69625	-0.91139	1.82429	-0.44207	0.691626	0	0	1.551721	1.414229	-0.14321	-1.11691	-0.97082	2.110303	2.096759	0	1.917352
Cediranib	1.908451	-1.03971	-0.52691	-0.98359	-0.89285	0.545346	0.421883	-0.53721	-0.0005	0.276219	0.076243	0.221318	-0.33006	0.145889	-0.51493	-0.58917	-1.19609	0.339756	-0.63058	0.727468	0.000501	-0.69295	-0.88796	0.554186	0.482824	0.956894	1.384179	-0.96613
Cisplatin	2.815412	0.10438	-1.4248	-0.8931	-0.88654	3.471348	1.858656	3.570654	2.460134	-2.36995	-0.30293	1.697284	-0.39762	-1.02761	2.060713	1.575439	1.25097	-0.32055	4.22724	2.446624	-1.17028	-1.66467	-0.1111	-3.03942	3.782731	-0.10438	-0.38763	2.048688
Crizotinib	2.332746	-1.04991	0.781381	0.40499	-1.66159	-0.11577	-0.24439	-3.90585	-1.65402	-0.24527	0.783482	-0.00452	-2.86191	0.004521	0.066809	0.728065	0.496944	-3.90585	-3.90585	1.105269	-0.29195	0.929724	-1.15263	0.318126	2.563288	1.629139	-3.90585	1.324072
Cyclophosphamide	0.583633	-0.21549	-0.07978	-0.01414	-0.90025	0.738971	0.51647	0.30216	-0.13763	-0.24058	0.014137	0.035719	-0.57138	-1.03746	0.332093	-0.58877	0.498226	0.044805	2.564917	-0.20464	0.614759	0.059001	-0.16004	-1.08925	-0.57858	0.522742	-0.73011	0.595085
Cytarabine	1.405571	0.308041	-1.43542	-1.88012	-1.87712	3.304477	1.142473	3.414585	0.35665	-1.05056	-0.70074	1.322725	-0.85394	0.052188	0.939716	-1.49196	-0.13745	-0.05219	3.872158	2.307007	0.284259	-2.06539	-1.27363	-2.47161	3.728686	-0.15583	1.187186	-0.32527
CZC24832	1.7111485	-1.03106	0.31018	-0.62034	-1.38257	0.402594	0.23753	-5.78302	0.371404	0.004849	-0.09807	-0.32005	-0.46062	-1.25278	0.749929	0.002426	-0.00243	-5.78302	-5.78302	0.894827	0.487023	-0.23676	0.066666	-1.06349	0.988352	1.858648	-5.78302	0.597979
Dabrafenib	0.356482	-1.51943	-0.78817	-0.73725	-1.32697	0.05956	-0.18461	0.394662	0.710803	0.195951	-0.67722	-0.36214	0.08502	-1.62116	0.65048	-0.10116	0.085646	0.771225	1.894848	1.065044	0.819591	-0.48915	-0.28747	-3.37327	1.599517	0.411103	-0.05956	0.452266
Dactinomycin	2.547679	0.598975	0.399344	-1.10927	-0.83319	2.157011	0.947387	-0.03575	-0.32781	0.600369	0.330751	0.983967	-2.08224	-1.3977	-0.03427	-1.08141	-1.17074	-0.76654	1.196445	0.841878	-0.20856	0.034273	-1.0152	0.15907	0.064289	1.819787	-1.79293	-0.24949
Dactolisib	0.0023565	-0.4266	1.971479	-0.91329	-3.5977	0.936031	-0.87568	-1.85754	1.107534	1.355577	-0.41091	0.688492	-0.8558	-1.02003	-1.05641	-0.98621	1.080547	0.385598	0.115434	2.265431	-0.12722	-1.56129	-2.29683	0.693825	2.602259	0.172864	0.845818	-0.00236

Daporinad	0	-3.13144	-4.28349	0	0	0	-0.24411	0.719938	0	0	0	0	-4.2603	-3.56785	0	0	-4.55986	-4.23725	-2.30544	0	-3.78327	-3.8954	0	-4.68219	0	0	-0.96702	-5.17196
Dasatinib	3.949968	-1.56834	0.619297	0.56408	-1.83016	3.583073	0.504656	0.077692	-2.552	0.225657	-0.2874	-0.02224	1.522631	2.233265	0.800527	-1.04607	-1.98129	-1.4513	-0.23696	2.339013	-1.84181	-0.99012	-1.45812	-1.47028	0.022241	3.930226	1.943505	-1.30635
Dihydrorotenone	1.473176	0.57585	-1.81871	0.603194	1.439438	-2.10144	-0.00104	-0.74149	1.898727	-2.57604	1.344559	-0.50292	-1.02181	0.362115	1.881618	-1.81256	0.857437	-0.74149	-0.74149	1.884858	1.536978	-0.62838	0.877309	0.00104	-1.41924	-0.47692	-0.74149	0.610516
Dinaciclib	3.482621	-2.1959	-0.43304	-1.09646	-2.18331	0.115065	-0.11507	1.528995	0.860176	-0.54934	0.600056	-0.63576	-1.68464	-1.54295	0.322078	-1.78387	-0.49734	1.528995	1.528995	0.444296	0.162285	-0.50934	-1.35462	-0.62602	1.846838	1.477358	1.528995	2.027655
Docetaxel	2.57462	-0.37376	-1.05648	-0.3552	-1.07794	0.378786	0.555927	3.042623	-0.52172	-0.13301	-0.81115	0.407399	-1.93079	-0.33582	0.550019	-0.93999	-0.28344	-0.14347	1.896641	2.339838	0.089742	0.424892	-0.50071	-0.08974	2.074322	3.28373	2.230269	1.177937
Eg5_9814	2.34355875	-0.63608	-0.02663	-1.74565	-0.61214	0.204412	0.258493	1.41555	0.310065	-1.72335	-1.28626	-0.1847	-2.87572	-2.04839	0.716597	-1.95316	-0.24795	1.41555	1.41555	-0.49565	0.026627	-0.60724	0.122572	-0.48967	1.555472	2.329004	1.41555	3.034521
Elephantin	2.494781	0.249088	-0.36188	0.559303	-0.00634	2.484905	1.440536	-4.13352	-0.23234	1.714516	-0.35943	0.45498	-0.76929	-1.85341	0.182675	0.698171	-1.21178	-4.13352	-4.13352	1.225643	-0.58388	0.006337	-0.81758	-1.31504	1.705919	1.753036	-4.13352	2.549368
Entinostat	1.0997305	-0.42378	2.836271	-0.31606	-1.20659	-0.02221	2.394892	-3.01274	-0.27886	1.755827	1.792716	1.666032	2.421003	-1.00041	0.022206	2.683133	-0.90074	-3.01274	-3.01274	-1.07044	2.3382	0.688833	-1.02643	-0.66406	3.753692	2.095545	-3.01274	1.871329
Entospletinib	1.19066	-1.02021	1.330537	-0.08926	-1.19115	1.108594	0.657504	-4.37155	-0.91919	1.04942	-0.34728	1.487836	0.197495	0.359245	0.710367	0.08926	-0.77534	-4.37155	-4.37155	-1.51193	-0.25987	-0.11526	-0.6117	0.417275	0.659449	1.733967	-4.37155	1.587648
Epirubicin	2.0177485	-0.28793	-0.55424	-1.63991	-1.53039	1.148096	1.640486	1.417789	-0.63522	0.365095	-0.99085	0.779761	-1.89469	-1.54125	0.019898	-1.25764	0.257078	-0.0199	2.964984	3.513316	1.537257	-0.75463	-1.71008	-2.39461	1.357499	-0.57917	0.033171	0.374034

EPZ004777	0.943671	-0.9082	-0.35146	0.024037	-1.18311	-0.01344	0.1603	-0.06078	-0.02116	-1.04428	0.144321	-0.42404	-1.02884	-1.09952	0.416252	0.065311	0.139142	0.008938	1.501631	0.317563	-0.00894	0.261981	-1.9154	-1.20828	0.358928	0.0833	-0.59154	0.675506
EPZ5676	1.733975	-0.72207	0.008548	0.045506	-1.63963	1.244034	0.41367	1.024245	-0.43604	-0.76103	-0.16278	-0.70276	-0.53167	-1.4452	0.330725	-0.14581	0.361138	-0.00855	2.49638	0.350394	0.621104	-0.08434	-0.58484	-0.19653	1.835805	0.705365	0.077292	-0.34512
ERK_2440	0.698192	0.663535	-0.62577	-0.0236	0.753458	-0.90007	-0.48951	-2.62418	0.227209	0.273645	1.64616	1.051722	-0.22209	-0.60012	0.798599	0.023596	-1.82844	-2.62418	-2.62418	-1.02882	0.136095	-1.69374	0.20319	-3.23721	0.565445	3.02057	-2.62418	3.885539
ERK_6604	0.8202725	-0.04923	0.299488	0.84106	0.049233	-0.63141	-1.56937	-2.92353	0.909361	1.146955	1.232814	1.097819	-0.68083	-0.09226	0.748252	-0.40292	-1.2492	-2.92353	-2.92353	-0.94202	0.167856	-1.46271	0.204677	-3.27099	0.468726	1.586609	-2.92353	3.685622
Erlotinib	1.720862	-0.98733	-1.39604	-1.40436	-0.88356	0.061467	-0.41224	1.83372	0.40056	-1.92627	0.005674	0.554572	0.494809	-0.16139	0.725655	-0.69703	-0.87749	-0.62346	-0.22647	-0.93012	0.982579	0.828889	0.435965	-0.00567	-0.08983	1.03587	0.192278	0.467732
Fludarabine	2.7800485	0.701184	1.028996	-1.86221	-1.49957	2.373334	1.051035	-5.69794	-0.043	0.233931	0.539376	0.679273	-0.41787	0.11902	0.46274	-0.13632	-1.04836	-5.69794	-5.69794	-0.53379	-0.57644	-0.86273	0.042998	-1.24511	0.809805	0.625723	-5.69794	0.975698
Foretinib	1.4328065	-0.0403	0.040302	-0.42629	-1.07216	-0.83724	-0.23888	-0.0726	0.397908	0.287856	0.231534	-0.53782	-2.17854	-0.53706	0.184937	0.328378	-1.35303	0.972528	2.366934	0.409588	0.096982	-0.06947	-0.25181	-0.07319	2.16852	2.032897	2.97751	-0.13501
Fulvestrant	1.34222	-1.24801	-0.03667	-0.58142	-1.62553	1.071023	0.300039	0.48473	0.407184	-0.7046	0.074823	0.11039	-0.33951	-1.81964	0.160472	-0.78931	-0.07771	0.036666	1.475659	-0.23569	0.429875	-0.64661	-1.08595	-0.7049	0.585835	0.668336	-0.48976	0.661824
Gallibiscoquinazole	1.9547345	0.311087	-0.03183	-0.7222	-0.76352	2.100627	1.224867	-3.00798	0.050145	-0.65602	-1.14152	0.154307	-0.08402	-0.27884	0.344272	-1.02082	-0.05631	-3.00798	-3.00798	0.870833	0.440969	0.031833	0.418874	0.51178	0.390137	1.525564	-3.00798	-0.54971
GDC0810	1.503287	-0.77275	0.554777	-0.49798	-1.93843	1.621233	0.711795	0.415895	-0.0128	0.04404	0.249253	-0.10575	-0.90759	-1.27954	-1.0569	-0.72405	-0.91697	0.560388	2.063626	0.494632	1.074761	-1.10441	-1.04122	-1.45238	0.987367	0.0128	-1.02227	0.246117

Gefitinib	-0.258115	-0.65234	-0.6022	-1.54494	-1.49381	1.860397	0.702017	0.543226	0.46999	-1.46284	-0.49574	0.731849	-0.01997	-0.31045	0.523048	-0.58984	-0.45333	-1.67511	0.740259	0.288859	-0.16418	0.060996	-1.57696	0.019973	1.562461	1.358983	0.341763	0.609251
Gemcitabine	2.045894	-0.89999	-2.748	-4.29019	-2.08161	4.066158	1.383497	5.248462	0.910527	-3.98804	0.77643	3.814238	-3.17966	-0.94038	2.447494	0.711677	0.059003	-2.95508	1.447237	4.825151	-0.059	-3.61522	-0.59347	-4.32044	5.954268	-0.88775	2.393271	-1.4801
GNE-317	0.952981	-0.41762	1.598707	-0.45024	-2.74471	0.265925	-0.95725	-1.44022	0.146562	-0.26062	2.328066	0.583906	-0.23928	0.145684	-1.26366	-1.08665	-0.47241	0.945341	-0.07352	0.558087	0.262743	-0.84589	-1.54745	0.148496	1.697862	-0.00369	0.987239	0.003688
GSK1904529A	2.9690475	-1.1391	1.224869	-0.88785	-1.64561	0.36909	1.24588	-0.11608	1.003	-0.22773	-1.3339	1.549574	-0.08342	-0.86288	0.703619	-0.53127	-0.21515	0.444543	0.966039	0.875895	-0.94253	0.052138	-0.05214	-0.41072	1.844204	0.113751	-0.57997	1.137081
GSK2578215A	0.9759795	-0.5817	-0.2644	0.267352	-0.55434	2.04198	0.262478	-1.54111	0.332315	-0.39213	-0.88351	0.421669	-0.66864	-2.577	0.392039	-0.39886	-0.67169	0.006879	2.406135	0.961961	1.246863	-1.22844	-0.00688	-1.18231	0.953758	1.295247	-0.67669	0.773379
GSK2606414	3.281032	-1.70439	0.029482	-1.52581	-1.58898	-0.70534	0.343928	-4.29893	0.893197	0.436516	0.560668	0.66092	0.280962	-0.18066	0.553967	0.841676	-1.12959	-4.29893	-4.29893	1.010836	2.025988	-0.21472	-0.02948	-0.21167	1.027988	0.081219	-4.29893	-0.24488
GSK343	1.9045405	-0.0761	0.614598	-0.85504	-0.54976	1.554477	0.576096	-3.30909	0.378786	0.135671	0.253991	-0.52241	-0.39846	-0.26471	0.672894	-0.50632	-0.45259	-3.30909	-3.30909	1.105903	-0.51493	0.0761	-1.06172	0.174453	1.99521	0.893017	-3.30909	0.281317
GSK591	0.6940625	-1.191	0.019062	-0.19841	-1.68056	1.807259	0.653689	-0.86504	0.46565	-0.59481	0.322575	0.388924	-0.6615	-0.32358	1.166081	-0.49312	-1.14116	-0.01906	2.024272	-1.07429	1.375388	-1.50374	0.407507	-1.84619	0.556939	1.794071	-1.82999	0.549412
I-BET-762	2.3241315	-1.12848	0.751163	-0.01595	-0.79565	0.158041	1.919415	-4.31108	0.076916	-0.03221	0.598808	1.9069	0.015954	-1.92652	1.356628	-2.16859	-3.29636	-4.31108	-4.31108	-2.18085	-0.4162	-2.51178	0.473611	0.909747	3.360486	0.118681	-4.31108	0.508599
I-BRD9	0.8868465	-0.85257	-0.61491	0.679546	-0.45192	0.881122	0.370216	0.197147	-1.30298	0.071993	0.498038	0.608579	-1.14014	-2.46377	-0.37152	-0.29925	-1.37353	0.417053	2.430327	0.680524	1.086728	-1.08948	-0.56416	-1.58106	1.81884	1.360739	-0.76361	-0.07199

IAP_5620	1.240385	-1.66938	-0.17163	0.326313	-0.1951	1.192305	0.454501	-5.97191	0.779721	-0.37923	-0.10701	0.649842	-1.51941	-1.73389	0.723565	-0.26927	0.924993	-5.97191	-5.97191	0.107007	0.875562	0.225243	-0.49429	-0.21016	0.595957	0.7659	-5.97191	0.348402
Ibrutinib	1.641597	-1.24057	-0.9327	-0.64613	-0.45688	0.11373	0.92928	-5.62941	0.848145	0.110621	0.314273	-0.37099	-0.37613	-0.42246	0.801766	0.090928	-0.09093	-5.62941	-5.62941	0.675704	0.671887	0.573931	0.226604	-1.31796	0.541011	2.193433	-5.62941	-0.20306
IGF1R_3801	2.924248	-0.20517	2.164034	1.166112	0.038349	-1.33966	1.416605	-1.82328	-0.13061	-0.00394	0.574198	0.988147	-2.63741	-1.70783	0.679266	1.667901	-0.60718	-1.82328	-1.82328	-1.11932	-0.25924	1.096123	-0.26649	0.003935	3.232937	3.402399	-1.82328	1.450834
Ipatasertib	0.534734	-0.87045	0.071542	-0.14781	-2.12728	1.349301	-0.60776	-0.89732	-0.64472	-0.00943	0.115707	1.114105	0.152625	-0.03684	-0.59954	-1.15117	-0.52825	0.693807	-0.15727	0.362361	0.140518	0.30924	-1.46593	-0.78258	0.685786	1.269386	0.009432	0.263603
IRAK4_4710	2.09176	0.323656	0.565655	-0.0544	-0.42686	-0.45624	-0.4462	-5.24353	0.976456	-0.41117	0.411354	0.622841	0.054396	-0.86567	0.90502	-0.291	1.273421	-5.24353	-5.24353	-0.60072	1.232122	-1.59399	0.870303	0.319815	-0.38468	1.013221	-5.24353	0.367263
Irinotecan	3.2111255	0.56867	-2.53095	-2.09295	-0.49634	1.251146	1.29138	2.928308	0.703026	-1.8007	0.194331	1.133823	-2.07428	-1.18729	0.384858	0.66754	-0.97351	-0.19433	1.886509	3.072588	-0.40243	-1.26472	-1.47262	-1.63401	3.30858	-0.71395	0.914532	-0.7547
IWP-2	0.523167	-1.45413	0.04651	-0.40426	-0.88515	-2.04145	-0.63152	-3.31067	0.627833	-0.44543	0.259135	-0.04651	-0.12573	-1.32253	0.632894	0.361497	1.188514	-3.31067	-3.31067	0.564267	0.112204	0.359919	-0.8338	0.205927	0.831767	0.320208	-3.31067	0.301508
JAK_8517	4.502072	-0.28663	0.587289	1.497178	-0.39532	-1.05944	0.742125	-3.28946	0.520598	-0.92081	-0.09694	0.096935	-1.65086	-0.29673	0.418125	0.671453	0.672454	-3.28946	-3.28946	-0.86182	3.519531	1.440535	-0.1747	-0.57716	1.708988	3.605932	-3.28946	1.536987
JAK1_8709	2.1567905	-1.56585	1.325936	-0.09248	-1.14466	0.573693	-0.76248	-5.04359	-0.57774	0.412666	0.190091	0.025548	0.288324	-1.13284	0.442995	-0.02555	0.461174	-5.04359	-5.04359	-1.43495	0.814461	-1.52433	0.544961	-1.22951	1.44191	0.827992	-5.04359	0.845115
KRAS (G12C)	2.4666025	0.134078	0.2522	0.52112	-0.33141	0.000272	-0.84822	-5.28624	1.618224	-0.1366	0.727929	0.940241	-2.15647	-1.04245	0.827866	-1.42414	-2.20396	-5.28624	-5.28624	1.807553	1.882524	0.01457	-0.00027	-1.81283	0.923459	1.382181	-5.28624	1.876773

Inhibitor-12																												
Lapatinib	0.833056	-1.68746	-0.56819	-0.68571	-1.92052	2.427609	-0.00486	-1.68974	-0.25567	0.004856	-1.58963	2.037793	0.378273	-1.43312	1.742499	1.312888	-0.43811	-0.78792	0.964946	1.421464	-1.83571	1.698711	0.504693	-0.13668	2.827064	1.401818	0.210319	-0.57126
LCL161	2.5244375	-2.09642	0.562798	0.357636	-0.51531	-0.42198	-0.19477	-5.57651	-0.62147	-0.91696	0.273357	0.069476	-0.0407	-0.31785	-0.28451	0.342923	0.040696	-5.57651	-5.57651	1.063807	0.91728	0.398867	-0.73524	0.244933	1.352514	1.021581	-5.57651	0.050033
Leflunomide	1.568213	-1.33595	0.032784	-0.18995	-0.20823	0.020735	1.124642	-5.32881	0.577022	-0.37971	0.221509	-0.83938	-0.46313	-0.1677	0.377237	-0.46187	0.476534	-5.32881	-5.32881	0.697614	-0.02074	0.324351	0.104731	0.553821	1.022041	-0.51034	-5.32881	0.548672
LGK974	1.100011	-0.95406	-0.73719	-0.18231	-0.65366	-2.58266	-2.58354	2.130797	0.859132	-1.30846	0.767089	-0.40902	0.044544	-0.11338	0.495854	-0.42174	0.844482	-0.33628	3.076295	1.115029	0.756019	-0.18369	0.508041	-0.04454	0.616535	0.575525	-0.6252	0.217994
Linsitinib	0.0530685	-0.73589	1.060338	0.331884	-0.54241	-0.31762	1.728192	2.191797	-1.0816	0.838387	-0.26416	0.236907	-0.21342	-0.38499	-0.90295	0.400845	-0.60668	1.314653	0.231441	-0.27283	-0.58469	0.973188	-0.05307	-0.7963	3.029338	1.92501	1.122073	-0.08827
LJI308	1.644173	-1.01073	0.047303	0.121995	-2.14887	1.039268	0.31371	0.971602	-0.0473	-0.83475	-0.3565	-1.05954	-0.73763	-1.26214	0.586337	-0.2115	-0.2404	-0.36531	1.863505	0.365896	0.629636	-0.31711	-0.9865	-1.11514	1.157448	1.075537	0.365694	0.202356
Luminespib	3.6523065	2.550182	2.932493	-2.01305	-2.07759	4.303324	0.552926	2.227509	-0.93369	2.753366	-1.46412	2.509525	-2.73282	-1.69477	-0.25477	-1.86192	-1.60738	-0.13905	1.634861	2.253511	0.283771	-1.8982	-0.9498	-2.27301	2.421976	4.111295	-1.9396	0.139047
LY2109761	1.329259	-0.4069	0.640116	0.883008	-0.62948	0.306899	-0.40707	-5.51132	0.912145	-0.05335	0.313184	0.613241	-0.61373	-0.58173	0.592933	0.146228	-0.47724	-5.51132	-5.51132	1.011655	1.045186	0.084294	-0.01643	-0.27377	0.016427	1.985584	-5.51132	-0.99085
MG-132	4.0448825	-0.08142	-0.23533	-0.5061	-0.56472	1.04207	0.303083	1.263771	-0.35996	-0.60923	-0.39297	-0.26196	0.213197	0.654186	-0.09064	-0.79791	0.573701	0.358926	3.082703	0.227797	-0.39035	0.223543	0.148062	-0.6436	-0.25396	0.905538	-0.18625	0.081415

MIM1	2.200918	-0.77402	0.700112	-0.62495	-1.75921	1.092897	0.045966	1.765446	-0.2093	-0.04597	-2.17288	1.089717	-1.56532	-0.49051	0.287705	-0.59084	0.864158	-0.77109	1.104604	1.378837	1.110072	-0.40944	-1.10188	-1.38446	0.318888	0.925216	-0.16722	1.70554
MIRA-1	1.6884955	-0.59142	-0.10293	0.053993	-1.11677	1.693095	0.315966	1.005287	0.131669	-0.52193	-0.30608	-0.0677	-1.0037	-1.35464	0.528324	-0.2639	0.193052	-0.05399	2.171691	0.442162	0.371694	-0.25522	-0.23153	-0.63709	1.338963	0.989578	-0.2728	0.146936
Mirin	3.912373	-0.39183	-0.02235	0.171533	0.022351	0.233228	0.412078	-5.47587	-0.31962	0.260021	2.952822	1.397175	-1.01929	-1.11834	0.667045	0.635331	-1.15675	-5.47587	-5.47587	-0.74979	0.266602	-0.49259	-0.71771	-0.63407	2.590417	4.015385	-5.47587	0.724786
Mitoxantrone	2.880656	0.908765	-0.47299	-2.38509	-0.51489	1.817292	1.322106	-1.3349	0.387365	-0.32368	0.606945	2.736165	-1.72303	-2.31597	0.539624	-1.50974	0.323678	-1.3349	-1.3349	2.892387	0.954248	1.448628	-1.51303	-0.99945	4.505364	1.321099	-1.3349	-0.63393
MK-1775	2.2418265	-0.56382	-0.78711	-0.91822	-1.41163	-0.08343	0.174143	0.856746	-0.37073	0.849951	-0.15504	0.363533	-0.76503	0.083433	0.387786	-1.61207	-0.77958	-1.74366	1.885447	1.109421	0.383778	0.764918	-0.17214	-0.32404	2.365776	-0.61089	0.3415	0.188168
MK-2206	-1.223164	-0.81929	0.581083	-0.7279	-3.19751	1.186767	1.252708	-1.64466	0.232053	0.882383	-1.68014	0.254221	-1.65558	-0.26816	-0.23205	0.593256	-1.59109	-1.13103	-0.63286	1.29607	-0.97838	0.58774	0.463965	0.502323	3.352898	1.987838	1.255469	-0.99646
MK-8776	-1.0378285	-0.3913	0.45383	-1.70771	-1.90696	2.251515	1.138391	-1.21255	-1.50693	0.158255	0.758247	0.789984	0.482085	-1.42759	0.787467	0.201613	-0.15825	-2.15465	2.52751	1.729627	0.393576	-0.19437	-1.08075	-0.24038	1.302254	-0.82855	-2.3929	0.879985
ML323	0.956181	-1.40652	-0.26872	0.094043	-0.61133	-0.68464	0.135365	-4.96035	1.342014	0.365196	1.009963	0.329511	-0.78422	-0.11663	0.856121	-0.81657	0.909695	-4.96035	-4.96035	1.489082	0.585883	-0.33129	-0.09404	0.503598	1.292834	-0.16208	-4.96035	0.386828
MN-64	1.430197	-1.59401	0.020516	0.153508	-1.61279	0.079655	0.784784	-5.56712	-0.12811	-0.16316	0.298754	-0.01852	-0.2026	-0.49678	0.018517	-0.79492	-0.24238	-5.56712	-5.56712	0.562205	0.169619	0.444637	0.52987	-1.30083	1.928554	1.31258	-5.56712	0.579122
Navitoclax	0.52338	0.673218	-0.7614	-0.78278	-1.49937	-0.16218	-3.29339	-0.7125	1.087081	-1.13543	0.609108	0.162179	0.640865	-0.69602	-0.6169	0.17957	0.726638	2.163184	5.273672	0.626513	-0.63052	-1.53085	-1.56842	-0.32194	-1.10722	1.547052	2.492273	3.076541

Nelarabine	2.138748	-0.04976	0.525512	-1.12338	-0.57192	1.659777	0.573453	-6.48253	0.292542	0.033765	0.365863	-0.29879	-0.39002	-0.64385	1.050573	0.215239	0.450516	-6.48253	-6.48253	0.673839	-0.09407	0.415382	-0.03376	-0.06547	0.966356	0.842367	-6.48253	-0.50656
Nilotinib	-0.300092	-0.55407	0.890783	-0.38551	-0.64898	0.557372	0.167104	-0.11152	0.249733	-1.10131	-2.13884	0.580864	0.019188	-1.2877	1.42752	-0.01919	-0.49213	0.730596	2.097147	1.193025	-0.85552	0.347805	-0.85962	-0.11924	1.506553	1.90973	1.034653	-0.59031
Niraparib	2.398258	-0.78221	0.022171	-1.22065	-0.45098	0.424522	1.01812	-5.05333	-0.22887	-0.06652	0.426759	1.113232	0.166637	-0.79383	-0.02217	0.211878	-0.32876	-5.05333	-5.05333	0.140667	0.828647	0.702072	-0.12566	0.091711	2.596895	-0.80985	-5.05333	0.042145
Nutlin-3a (-)	1.373963	-0.04215	-0.40632	0.200668	-0.98823	1.744857	0.04478	1.596393	-0.00023	-0.5695	-0.53616	0.144155	-1.08182	-0.55415	1.142206	-0.36896	0.000229	-0.55817	-2.61376	0.014705	-0.09909	-2.28681	-0.00175	0.153043	1.637082	0.860036	-0.56839	0.101171
NVP-ADW742	2.175224	-0.04082	0.034199	-0.48214	-1.58446	-0.27572	0.49307	2.301354	-0.67433	0.624124	-1.03492	-0.08148	0.435598	-0.27671	-0.49137	0.440351	-1.08923	1.275822	0.387476	-0.43678	-0.05428	2.275757	0.018783	-0.01878	3.36218	1.247758	2.870845	-1.4838
Obatoclax Mesylate	1.9996045	-1.24416	1.509366	-0.04878	-0.73422	0.070677	0.511303	-2.14929	0.972607	0.58307	0.062049	0.317767	-0.38765	-0.62987	-0.17771	0.048785	-0.72555	-2.14929	-2.14929	0.52192	0.328376	-0.49159	0.146809	-0.87353	3.872158	-0.89627	-2.14929	1.174011
OF-1	1.393354	-0.9166	-0.47288	2.367941	-0.04968	-0.82425	-0.02458	-3.84984	1.46247	-0.87298	1.192472	0.055353	-0.65196	-0.03494	1.272019	-1.48967	2.091271	-3.84984	-3.84984	0.42305	2.67456	0.166842	2.183313	-0.10768	1.889028	1.267863	-3.84984	0.024577
Olaparib	2.121165	-0.42418	-1.77413	0.002158	-1.45593	0.777567	0.958545	0.684762	-0.68273	-0.35878	0.145563	-0.05156	-0.89487	-0.80475	-1.04232	-0.23849	0.44564	0.898333	2.095515	-0.1581	1.605671	0.065476	-0.93181	-0.98834	0.106806	-0.00216	0.088093	0.291524
OSI-027	6.546398	0	0	6.081594	4.546593	0	0	0	0	0	5.668605	0	0	0	6.327685	0	6.049108	0	0	0	0	0	5.824758	0	7.617244	7.090581	0	0
Osimertinib	1.2706575	-1.18363	-0.47251	-1.11219	-0.94601	1.576667	0.264174	0.121595	-0.40372	-0.84622	0.945182	0.724826	0.0566	-1.02889	-0.32476	-1.37281	-0.89228	-0.88154	0.470743	0.570346	0.542492	1.123014	-0.05563	-0.97435	0.055627	0.742832	-0.35044	0.922246

OTX015	3.3272515	-0.94999	1.912674	0.027207	1.090523	0.300824	1.908043	-3.06375	-0.37192	0.955208	0.507945	2.853163	-0.2274	-2.90081	0.937951	-2.31985	-2.50244	-3.06375	-3.06375	-2.6367	-0.55198	-2.75708	-0.02721	1.852423	4.618475	0.251069	-3.06375	1.737951
Oxaliplatin	2.2888635	0.377883	-0.28225	0.292173	-1.03045	0.300798	-0.14821	1.102011	-1.30962	0.065884	-0.8303	1.41365	-1.56304	-1.17235	-0.23073	0.113909	-0.12408	0.3134	2.036106	2.431446	0.732859	-0.15814	-1.59256	-1.51691	2.297972	-0.56638	0.635984	-0.06588
P22077	2.1441935	-0.05834	0.392564	-0.12628	-1.68871	-0.88738	0.81267	1.071207	-2.12795	-1.62739	-1.96356	-0.00359	-1.38974	-0.56549	-0.59234	-1.39536	0.216188	0.309729	3.096651	0.297929	-0.6451	0.438264	-3.8437	0.003586	1.566675	0.547748	0.660649	0.632536
Paclitaxel	2.7438655	0.099246	-1.03376	-0.03015	-2.98545	0.851777	-0.30537	-0.53251	1.241882	0.391366	-1.78607	-0.43571	-3.59224	-1.67927	1.973718	-2.63272	0.030151	-0.09011	1.963187	1.983699	-1.14789	-1.33935	0.800443	0.116114	2.881758	2.503285	1.065634	-0.22008
PAK_5339	1.973388	0.163483	0.542458	-0.93782	-0.99643	0.610127	0.322648	-2.90432	-0.39704	0.738172	-0.82114	1.00757	0.308991	-0.67084	0.863965	0.078309	-0.82478	-2.90432	-2.90432	1.078429	-0.94462	0.385465	-0.39622	-0.74613	0.450313	-0.07831	-2.90432	0.799399
Palbociclib	0.0322284	-1.07974	0.042608	-0.25296	-0.79996	2.109428	1.45995	-0.24087	-0.41906	-0.02457	-0.3267	0.42486	-2.49263	-1.15604	-0.71356	0.08592	-0.6324	-0.35555	2.855455	1.674748	-1.11471	0.884772	0.832467	-1.39306	3.380492	0.921602	1.09795	0.024572
PCI-34051	1.75552	0.217187	0.787897	-0.3875	-0.78751	0.821196	0.951662	-5.35347	-0.34283	-0.38796	-0.59579	0.280712	0.149739	-0.19826	1.185587	-0.33361	-1.03751	-5.35347	-5.35347	1.294383	-0.05216	0.55866	0.052164	-0.13546	1.583339	1.394984	-5.35347	1.054678
PD0325901	-0.479444	-0.10291	-0.00907	0.913054	-0.75102	1.288458	-0.66872	-1.02466	-0.71474	1.133286	2.284964	-0.88949	0.00907	-1.16021	0.326688	-0.39571	-1.75214	0.036411	0.360211	1.347827	0.790645	-1.46199	-0.40089	-1.74706	0.488171	1.220666	0.336819	2.642878
PD173074	0.8676075	-1.06739	-0.01047	-0.5079	-1.15443	1.621614	-0.00579	0.132675	-0.6518	-0.4678	-0.35758	0.032929	-0.81719	-2.60449	0.425872	-0.42195	0.098858	0.005787	1.861084	0.426201	0.739915	-0.05311	-0.42282	-0.57953	1.21771	0.808637	0.227741	0.10835
Pevonedistat	6.251977	-0.32376	0.775311	-0.6005	0.139758	0.199926	1.709332	3.08564	-1.23509	-0.70495	0.478587	0.333149	-2.37369	-1.54245	0.293554	-1.58213	-0.54479	0.029539	3.612514	3.112504	-0.86791	-1.45385	-1.05484	-0.47189	1.228272	-0.02954	0.400099	-1.20866

PFI3	1.9877785	-0.4682	0.166354	0.508054	-0.67554	0.124899	0.110088	-5.8323	-0.5517	-0.0411	0.331357	0.443444	-0.42539	-0.72317	0.668356	-0.48923	0.053384	-5.8323	-5.8323	0.429733	0.741244	0.041097	-0.53199	-0.1458	1.606761	1.499583	-5.8323	-0.7941
Picolinici-acid	0.4134575	-1.65348	0.510085	0.43205	-1.19647	0.043322	-1.23443	-5.7924	0.193371	-0.07433	0.246003	0.09792	-0.83532	-0.45738	0.369502	-0.62312	0.816531	-5.7924	-5.7924	0.165169	0.218074	-0.04332	-0.49618	-0.08653	0.312407	0.524433	-5.7924	0.48032
Pictilisib	0.3029555	-0.75934	0.641402	-0.96266	-1.9473	0.647274	-0.55411	0.055877	0.120894	0.275981	-0.18104	0.60579	-0.12421	-0.17665	-0.8308	-0.57712	-1.31138	-0.26617	0.941715	0.04089	-1.36855	0.370599	-0.56844	0.433318	2.445403	1.613519	1.051627	-0.04089
PLX-4720	2.5054755	-2.03371	-0.14396	-1.47406	-1.87575	-0.74982	0.498353	-2.1657	0.216402	0.324003	0.35112	-0.53189	-0.81464	0.104261	-0.39492	0.694129	-0.59048	0.52508	1.018676	1.58623	1.754377	-0.10426	-0.22238	-0.95691	0.371635	1.045682	0.164938	-1.01011
Podophyllotoxin bromide	4.003004	-1.63228	-0.60073	0.3061	-0.38647	2.258177	1.64293	-0.73819	0.280794	0.605063	-0.01172	2.213343	-2.54738	-2.54455	0.011716	-1.42797	-0.99614	-0.73819	-0.73819	3.190498	0.708609	0.192734	-0.80824	-0.92555	2.913965	1.825614	-0.73819	1.738166
PRIMA-1MET	3.847475	0.081807	-1.56278	0.066214	0.712609	2.46375	2.145794	-5.23459	-1.844	-0.06621	-2.05336	1.425861	0.174623	-2.7886	0.247354	0.961118	-0.57334	-5.23459	-5.23459	-0.91084	-1.91004	0.164275	-0.33863	-1.8372	3.482722	2.320175	-5.23459	1.694372
PRT062607	1.5406595	-0.00282	1.009437	0.058558	-0.52491	-0.40904	0.002816	-3.62092	0.3363	1.631547	1.338997	0.897294	1.31495	-0.72589	0.069541	-0.33788	-0.88568	-3.62092	-3.62092	-0.15301	-0.02551	1.459147	-0.37271	-0.31042	3.355242	1.883583	-3.62092	1.494417
Pyridostatin	1.264933	-0.88068	0.494406	-0.19508	-1.42362	1.797703	0.411979	-1.83269	0.659643	-0.56382	-0.27143	-1.21973	-1.18274	-1.95992	0.843355	-1.29007	-0.3428	0.195081	2.215213	1.804667	0.627516	-0.27501	-0.33112	0.458972	2.172608	1.80326	0.542713	-0.89581
Rapamycin	1.3311085	-2.19227	0.449466	-1.06666	-2.96573	0.207549	0.321789	-1.00899	-0.20324	0.203243	-0.65624	0.216369	-0.2134	-0.48521	-0.53305	-1.06199	-0.23409	0.772592	1.811489	1.132869	-0.49936	0.541898	-0.4734	-0.6815	2.052758	1.616646	1.315127	0.707056
Ruxolitinib	1.040987	-0.5	0.230	-0.5	-1.0	-0.3	0.844	-5.5	0.539	-1.1	0.22486	0.006	-0.2	-1.355	0.514	0.215	0.84762	-5.5	-5.5	0.101	0.413	0.120	-1.0	-0.0	1.793	0.949	-5.5	-0.4

		2467	161	8564	5161	8725	689	8565	821	4166		749	5982		356	218		8565	8565	431	559	201	5193	0675	832	608	8565	0403
RVX-208	1.58481	0.162426	1.030654	0.781069	-0.76043	1.039413	1.044804	-5.3042	-0.51424	-0.19495	0.371322	0.394132	0.108269	-0.41043	0.565952	-0.17656	1.19044	-5.3042	-5.3042	-1.15471	-1.05916	-0.55341	-0.10827	0.411396	2.195006	0.642605	-5.3042	0.805972
Sabutoclax	1.359201	-1.16111	1.707519	0.832668	0.155186	0.48784	1.137573	-0.50211	-0.29788	1.480858	-0.02952	1.332261	-1.16104	-1.75926	0.393797	-0.48395	-0.53901	-0.50211	-0.50211	1.875634	0.029517	-0.24378	-0.8777	-0.52824	1.115612	1.321365	-0.50211	0.645186
Sapitinib	0.1345245	-1.76358	-0.82473	-1.49298	-1.17636	0.180332	6.55E-05	0.703466	-0.99428	-6.55E-05	-0.00985	0.416743	0.948258	-0.79512	1.271062	0.282849	-1.75165	-1.00189	-0.32185	0.741536	0.323072	0.446162	-0.55506	0.974574	-0.41234	2.421349	0.519148	-0.38834
Savolitinib	2.6016595	-0.22035	0.024161	-1.10173	-0.48448	0.73696	0.829944	1.582104	-0.39414	-0.8426	-0.33996	-0.3386	-2.8121	-0.16455	0.255983	-0.82922	1.157602	-0.25604	2.885974	-0.90014	0.308516	0.213648	-1.05105	0.12984	0.794681	-0.02416	0.934503	0.859631
SCH772984	1.1564515	-0.42272	-0.79693	1.400505	-0.34991	-0.71448	-0.90971	-0.07907	-0.18629	1.844392	3.592614	0.323454	0.966525	-0.41419	1.115371	0.060859	-1.97493	-0.06086	-0.10039	2.518069	0.385907	-1.23439	-0.82733	-2.57557	1.334616	1.072276	1.284122	2.011609
Selumetinib	0.793748	-0.0066	0.250572	0.979344	1.128228	-0.6646	-0.79664	-2.69931	0.006596	2.467288	1.878761	-0.03103	0.566182	-0.25682	1.95757	0.297929	-1.92722	-2.69931	-2.69931	0.699527	1.374143	-0.14826	0.456144	-1.75898	-0.42512	-0.06343	-2.69931	3.885104
Sepantronium bromide	1.075068	-0.03798	0.431724	-0.00506	0.200778	-0.65711	-0.0977	1.121853	-0.63225	0.005061	-2.08719	0.92662	-2.50756	0.110791	-0.01237	-1.54016	1.023291	-2.35136	1.110796	1.351099	0.449996	-0.61512	-2.07252	-1.42408	-1.37281	0.021821	0.503809	1.307853
Sinularin	1.235816	-0.94159	0.213907	0.112248	-1.09854	2.063653	0.253523	-4.14261	-0.01145	-1.41439	0.201037	-0.23727	-0.07742	-2.40977	1.096267	0.375435	0.011446	-4.14261	-4.14261	-0.0681	-0.58065	-1.15409	0.110217	0.475767	0.749115	0.088996	-4.14261	0.705536
Sorafenib	0.744579	0.548191	0.169209	-0.09732	-1.06146	-0.2171	0.561513	-0.07076	-0.17455	-0.43649	-0.98259	-0.30446	-0.97118	-0.72102	0.37145	-1.22712	0.305026	2.083773	2.959467	2.523742	0.185246	0.070764	-0.61887	-0.19654	1.566774	0.426515	0.296347	-0.3279

Staurosporine	1.1503465	-1.49368	-0.00411	0.004108	-2.42934	-0.45395	1.140678	0.407952	-0.62645	-1.93086	0.02295	-1.69162	-0.73824	0.656436	-0.18456	-2.17289	1.723988	-1.72508	2.02991	0.125939	1.098732	-1.69395	-1.13312	-1.05674	0.885283	1.091335	0.08421	0.144889
TAF1_5496	2.1505295	-0.01113	-2.09129	1.837996	0.844374	-2.27924	-0.80201	-4.02284	1.258708	-2.72955	1.078764	0.165462	-1.40235	0.121832	1.409909	-0.97552	2.502389	-4.02284	-4.02284	1.386994	2.896073	0.011133	1.418134	-0.65919	-0.54944	-0.23461	-4.02284	0.617766
Talazoparib	1.384895	0.517325	-2.9696	-2.51566	-0.86754	0.841221	-0.60915	1.663786	-0.62733	-1.46213	0.052652	0.146191	-0.93508	-0.00068	-0.50646	0.031606	0.000685	0.391447	1.800489	0.55147	0.417141	-0.77506	-1.90114	-2.55242	1.89465	-1.83708	0.419044	-0.17468
Tamoxifen	2.70728	0.024402	-0.56928	-0.92163	-1.26585	-0.43125	0.376095	-1.09863	0.03531	0.354031	-0.09565	1.279641	-0.91781	-0.54096	-0.16672	-0.37606	-0.83286	0.950566	1.2139	0.63152	0.354043	-0.0244	-0.49359	-0.7135	0.848146	0.666289	0.287392	1.46629
Taselisib	1.336799	-1.13376	0.060802	-0.20769	-2.57004	-0.43385	0.20783	1.026782	0.195582	1.135988	0.07915	-0.35928	0.272781	-0.23937	-1.794	-0.81033	-1.87432	-0.0608	0.759506	-0.75888	-1.53053	0.427968	-1.18906	0.328995	2.243208	4.573158	0.676757	-0.38266
Telomerase Inhibitor IX	3.8260305	0.747938	0.517046	-0.41252	-0.09851	2.23949	0.322393	0.739876	-1.4286	-1.3968	0.527198	1.368699	-2.01135	-1.78371	-0.34582	-1.22665	-0.7545	-0.83462	1.538909	2.100338	0.082405	-1.10445	-1.51725	-1.96427	1.039428	3.392565	-0.0824	-0.57613
Temozolomide	1.703485	-1.45081	0.345147	-1.02878	-1.60252	1.069592	-0.2972	0.679868	-0.39506	-0.06359	-0.20486	-0.52235	-0.88004	-1.09336	0.972426	-0.51497	0.802197	0.279306	1.330191	0.806336	0.063594	-1.0624	0.296014	-0.93791	1.214194	0.526939	-0.84308	0.758477
Teniposide	5.229161	1.639384	-0.49225	-0.47125	0.276791	-0.35276	1.103742	-1.05759	0.144	-0.56757	1.095522	1.213687	-2.29605	-2.01998	0.603975	-0.92107	-0.36992	-1.05759	-1.05759	2.592868	0.477506	0.915957	-0.144	-0.92182	3.82324	0.58858	-1.05759	1.106682
Topotecan	4.7511465	0.332412	-1.04607	-1.5563	-0.08501	2.703563	1.051045	-0.49632	0.952616	-1.45355	1.239091	3.189602	-0.70745	-1.38773	1.095634	0.844449	-0.80319	-0.49632	-0.49632	3.572775	0.423778	0.085009	-0.92611	-0.47613	2.124503	0.523451	-0.49632	-0.3018
Trametinib	-0.5833445	0.465224	-0.81382	0.62826	-1.47014	-0.99405	-2.3053	-0.29362	-0.18852	0.397447	2.325552	0.037125	0.929456	0.035241	0.512502	0.868063	-2.61869	0.31105	-0.86247	4.576146	0.117275	-1.4626	-0.40439	-2.66061	-0.32823	-0.03524	1.488476	2.904506

Ulixertinib	1.3642035	0.060318	-0.73196	-0.24305	-0.04731	-0.33326	-1.09335	-0.16415	-0.25444	-0.29634	1.291716	0.420183	-0.27998	0.047308	0.285835	-0.87693	-1.16855	1.111797	0.537755	2.961412	0.525697	-0.44867	-1.20093	-2.71888	2.173	0.365505	0.820862	1.881632
ULK1_4989	6.6728135	-1.44534	-0.32656	0.876603	0.630771	0.262418	0.769447	-3.12083	-0.43332	-0.35773	0.943241	0.41885	-0.89109	-1.44073	0.645812	0.430132	0.207197	-3.12083	-3.12083	-1.30913	0.043518	-0.62507	-0.04352	-0.84073	0.337575	4.575618	-3.12083	0.621613
UMI-77	4.9573565	1.60713	1.600424	1.060428	-0.9619	2.428221	-1.15628	0.889731	1.287131	1.185376	-1.0733	1.667645	-1.07273	0.940806	-0.72222	-1.18136	-1.10648	-0.43721	-0.30511	1.201942	-1.0372	-0.99009	0.136187	-1.12904	2.939704	-0.13619	0.716457	-0.52456
Uprosertib	0.2495277	-2.88856	0.105273	-0.83476	-2.11458	0.115573	-0.83533	1.500742	-0.58283	0.223704	-0.63771	0.409325	-1.36514	-1.47911	-1.07718	-1.09281	-0.34263	0.22714	0.958719	-1.41081	-1.59403	-0.10527	0.48548	0.434887	2.651425	1.658163	1.452245	0.130333
VE-822	0.466704	-1.27513	0.527199	-2.04449	-2.69078	-1.96892	0.879328	-5.11815	0.628114	-0.03186	0.115505	0.031859	-0.5936	-1.78178	-0.59866	0.206892	0.853746	-5.11815	-5.11815	0.508543	0.80448	0.34072	-3.73988	-1.65358	0.721274	1.078565	-5.11815	0.759731
VE821	-0.0591335	-2.24146	-0.03817	0.393769	-2.38741	1.193022	0.781699	0.132457	0.342247	0.017008	-0.58346	-1.98121	-0.3935	-1.49993	0.74638	0.216779	-1.1502	-1.86211	0.36279	0.927142	1.084066	0.162724	-2.55521	-0.92403	0.8966	0.986026	-0.50417	-0.01701
Venetoclax	3.079027	0.238259	-0.34924	-0.1982	-0.99718	-0.68921	-0.48017	1.884846	0.976793	-0.21227	0.444019	0.964256	-0.32523	0.376512	-0.11311	-0.9209	0.721708	0.343443	1.539567	-0.57275	1.276567	-0.12121	-0.68561	-0.30522	0.531078	0.11311	-0.16178	1.42364
Vinblastine	3.802661	-1.99339	-2.8036	0.408296	-0.77696	3.486929	0.564508	1.480846	-0.66522	0.175237	-2.64918	-0.17524	-3.71669	-3.18269	-0.18664	-3.31964	0.274162	-1.08072	1.269991	3.242259	1.246879	-1.65725	-1.45384	-2.90607	1.566169	1.354025	0.398808	0.918216
Vincristine	6.0930385	-1.96617	0.144422	-0.76123	0.972947	3.411822	2.129683	-0.14442	0.788337	-0.96292	1.107274	1.835303	-4.02623	-3.38971	-0.17625	-1.95696	-0.41387	-0.14442	-0.14442	2.78295	0.87968	1.248338	-0.5527	-1.75736	3.829237	3.269603	-0.14442	1.885081
Vinorelbine	3.3652265	-3.05114	-2.16911	0.140863	-1.59949	1.25021	0.515182	2.333381	-0.69006	-1.68852	-0.07591	-1.29122	-2.55685	-0.85071	0.075912	-2.25208	0.376096	-0.34145	2.159789	2.660124	-0.54275	0.119722	-1.22772	-2.58188	2.996245	2.830072	2.356609	2.733449

Vorinostat	0.9739765	-0.379	0.426735	-0.42051	-1.74569	0.320714	0.547791	-0.67158	-1.03919	0.267008	-0.1699	-0.30198	-0.26676	-1.6151	-1.34321	1.785298	-1.00831	1.192904	1.469885	0.256086	0.503543	-0.54524	-0.74014	-1.12625	3.297315	1.392104	2.020221	0.169903
VSP34_8731	3.124468	-0.84829	-0.33789	-0.93475	-0.1251	0.584571	1.261042	-2.50736	0.103499	0.08057	0.886272	1.669274	-0.32579	-0.69645	1.689117	-0.02899	0.028988	-2.50736	-2.50736	-0.99687	1.061918	-0.12878	-0.99227	0.139235	0.743839	1.346244	-2.50736	0.573084
VX-11e	0.3719795	0.231474	-1.53125	0.149398	-1.7853	-0.07937	-1.91345	0.576655	-0.67177	0.052828	1.543972	0.69563	0.493238	-0.51123	1.368233	-0.74206	-0.76234	-0.05411	-0.30706	3.450986	-0.05283	-0.09997	-0.31668	-2.78784	1.779749	1.706927	0.925372	2.451842
Wee1 Inhibitor	2.8404495	0.206294	0.574956	0.268557	-1.53003	-0.83457	0.606971	-0.83079	-1.27737	0.084496	-0.01481	-0.22066	-1.21173	-1.68124	0.014806	-1.25146	-0.37589	-1.02074	1.378075	3.032705	1.23425	-0.04516	-1.27409	-0.32765	1.21766	1.389209	0.119871	0.510324
WEHI-539	3.230417	-0.30334	-0.01077	-0.1068	0.617752	1.562416	-1.14909	-0.23973	-0.58062	-0.59051	1.665733	-0.96585	0.431829	1.476562	-0.1209	0.010768	-0.24664	-0.63572	2.705185	1.265175	0.099496	-0.45113	-1.90521	-0.82576	0.624965	0.671034	0.148627	2.043782
WIKI4	3.3794075	-0.22601	-0.09302	-0.93213	-0.38378	-0.01064	-0.53703	1.490799	-0.10847	-0.88228	0.010638	0.31448	0.168146	1.186868	0.544687	-0.1327	0.124208	0.398754	0.268353	-0.39627	-0.32441	0.395401	-1.63577	-0.35645	0.311793	-0.04538	1.604922	1.993161
Wnt-C59	0.950057	-1.6151	-1.27459	0.751892	-1.34674	-2.57425	-1.57799	-4.94668	0.156389	0.127009	1.014307	-0.4595	-0.29632	0.044789	0.277648	-1.1227	0.542246	-4.94668	-4.94668	0.471274	1.500685	-0.63037	-0.04479	0.093218	1.560569	0.073307	-4.94668	1.124882
WZ4003	2.3495705	-0.86906	0.594884	-0.70681	-1.1008	-0.15992	0.937909	-4.62637	1.102331	1.122502	0.766478	1.614107	-0.00332	-1.84836	0.225438	0.535339	-0.70261	-4.62637	-4.62637	1.040259	-0.54419	0.159089	-0.26232	-1.20888	2.833468	0.749368	-4.62637	0.003322
XAV939	2.9689025	-1.30729	0.244139	-0.43612	-0.45345	0.899962	0.462803	-4.6055	1.068429	-0.3783	0.71828	-0.05663	-0.22633	-0.3143	1.887449	0.906323	-0.40189	-4.6055	-4.6055	1.847749	-0.94359	-0.74429	0.056628	1.184825	0.887464	0.737548	-4.6055	0.481209
YK-4-279	3.329641	-1.68972	-0.94349	-0.52493	-0.63671	-0.359	1.343982	0.079425	0.03675	-0.30069	-0.99498	0.102133	-2.56353	-0.40591	0.0915	-1.21386	-0.05221	-0.03675	2.284607	0.190448	0.71361	0.653869	-1.50067	-1.81955	0.464502	0.982114	1.759947	1.624603

Zoledronate	1.951523	-0.28129	0.282251	0.240976	-1.33331	0.552912	0.554137	-4.4104	0.273858	-0.19669	-0.26721	-0.38233	-0.48949	-1.04826	0.283518	0.048442	-0.03625	-4.4104	-4.4104	0.767397	0.744907	0.036251	-0.04863	-1.69435	1.334959	0.581098	-4.4104	0.377351

Supplementary Table 12: Enrichment of 22 immune cell types by CIBERSORT algorithm in TCGA_PAAD cohort.

TCGA_ID	B cells naive	B cells memory	Plasma cells	T cells CD8	T cells CD4 naive	T cells CD4 memory resting	T cells CD4 memory activated	T cells follicular helper	T cells regulatory (Tregs)	T cells gamma delta	NK cells resting	NK cells activated	Monocytes	Macrophages M0	Macrophages M1	Macrophages M2	Dendritic cells resting	Dendritic cells activated	Mast cells resting	Mast cells activated	Eosinophils	Neutrophils
TCGA.3A.A9IO.01A	0.0785599	0	0.013731	0.088163	0	0.13455903	0	0.00980587	0.01355512	0.07030867	0	0.05496351	0	0.12388409	0	0.19723347	0.02342519	0.07588658	0.11592468	0	0	0
TCGA.US.A774.01A	0.04999415	0.00270292	0.138028	0.140097	0	0.04703908	0.01624723	0	0.04775111	0	0	0.01887591	0	0.13985247	0.08517352	0.13159004	0.06079052	0.00488845	0.10048536	0	0	0.01653654
TCGA.HZ.A49H.01A	0.036696	0	0.148864	0.100094	0	0.25049485	0.02163669	0	0.0086902	0.0297798	0	0	0.01705986	0	0.05599823	0.13127837	0.06830743	0.00854151	0.08818393	0.01116826	0	0.02320657
TCGA.FB.A4P5.01A	0.1930499	0	0.177875	0.205384	0	0.12645512	0.01851929	0.00765178	0.01826146	0.00645123	0	0	0	0.05938443	0.03675341	0.09826915	0.02149634	0	0.02938062	0	0	0.00106808
TCGA.FB.AAPS.01A	0.1252376	0	0.040749	0.111778	0	0.14571113	0.01926971	0	0.04664564	0	0	0.07614524	0.00075201	0.2056925	0.02445305	0.1160464	0	0.00461742	0.08061307	0	0	0.00228941
TCGA.3A.A9IN.01A	0.0553333	0	0.083035	0.064134	0	0.22522046	0	0	0	0	0	0.09044108	0.01036858	0.0426847	0.06720303	0.22969176	0.02239506	0	0.02745254	0.08204113	0	0
TCGA.HV.A5A5.01A	0	0.02381399	0.053841	0.035549	0	0.28233507	0.01161412	0.02998769	0.04903772	0	0.00720531	0	0.00576498	0.15209589	0.04707256	0.18053221	0.03798683	0.0143923	0.05068522	0	0	0.0180862
TCGA.H6.8124.01A	0.0293792	0	0.060215	0.036793	0	0.15059139	0	0	0.03808522	0	0	0.02059031	0	0.39894624	0.01346571	0.0499456	0.12176198	0	0.0802259	0	0	0
TCGA.US.A77G.01A	0	0.05811722	0.003291	0.053311	0	0.28828875	0	0	0.02261967	0.00313508	0	0.02237685	0.02047994	0.08926879	0.046954	0.19487757	0.0700422	0.03596832	0.0912696	0	0	0
TCGA.3A.A9IJ.01A	0	0.00034697	0.005765	0.054022	0	0.15153831	0	0.02105526	0	0	0	0.12252626	0.14948195	0	0.01872187	0.33257982	0	0	0	0.14396227	0	0

TCGA.HZ.A8P0.01A	0.0231261	0.00561412	0.088937	0.054478	0	0.16534484	0.00595066	0	0.08334971	0	0	0	0	0.39207629	0.0234481	0.08256718	0.00082797	0	0.00974776	0.02313683	0	0.04139555
TCGA.IB.7644.01A	0.0141122	0	0.008804	0.094243	0	0.28839469	0	0	0.03842226	0	0	0.05310134	0.01389634	0.05007316	0.02510777	0.11979817	0.15725301	0.02095532	0.09913501	0	0	0.01670395
TCGA.IB.A7LX.01A	0.0516969	0	0.030714	0.145265	0	0.18212372	0	0.03186547	0.01863792	0	0.00410519	0.00515666	0	0.26012907	0.07279494	0.11822834	0	0	0.0784982	0	0	0.00078502
TCGA.2J.AABK.01A	0.1050806	0	0.123682	0.122621	0	0.19361604	0.01030935	0	0.01246128	0.04232636	0	0	0	0.11344688	0.05689852	0.20040338	0.01076762	0.00576931	0	0.00261773	0	0
TCGA.S4.A8RP.01A	0.0011733	0.03206663	0.039558	0.102374	0	0.16865724	0.01088967	0.00627198	0.02108786	0	0	0.00791358	0.03010899	0.11897368	0.10306903	0.18240706	0.07521956	0.01450453	0.07259552	0	0	0.01312906
TCGA.2L.AAQM.01A	0.0530978	0	0.01427	0.173204	0	0.16295372	0	0.01081388	0	0	0.20842746	0.02656653	0.07729695	0	0.03984966	0.1560112	0	0.01528453	0.0524816	0	0.00795453	0.00178795
TCGA.XD.AAUG.01A	0.0920464	0	0.122017	0.103017	0	0.25075307	0.00399785	0	0.00419938	0	0	0.01250349	0.08305996	0.02028154	0.00124608	0.1432455	0.02619019	0.03379311	0.09867878	0	0	0.00497146
TCGA.M8.A5N4.01A	0	0.00903183	0.017419	0.047217	0	0.10228719	0.02415463	0	0.02730228	0	0	0.04852127	0.01399697	0.22673863	0.09481642	0.25242341	0	0.03884227	0.03592247	0	0.00674371	0.05458284
TCGA.2L.AAQI.01A	0	0.05636595	0.040732	0.064276	0	0.18551778	0	0	0.02765801	0	0	0.0369622	0	0.34358932	0.03265068	0.14449522	0	0	0.05119723	0	0	0.01655591
TCGA.HZ.7919.01A	0.0081925	0	0.048376	0.102544	0	0.19872236	0.00843077	0	0.01487206	0	0	0.03467319	0.02223393	0.05345535	0.08700811	0.18485823	0.07397388	0	0.16265999	0	0	0
TCGA.HZ.8005.01A	0.010466	0	0.031794	0.053524	0	0.11908872	0.01635756	0.00405972	0.01701714	0	0.00569557	0.03429059	0.0070624	0.12389304	0.18020541	0.2906283	0.0066196	0	0.09103999	0	0	0.00825794
TCGA.IB.AAUM.01A	0.0166298	0.0492765	0.044958	0.163702	0	0.1644049	0.00871102	0	0.03353817	0	0	0.00905238	0.02549655	0.23685125	0.02576092	0.17157848	0	0	0.05004069	0	0	0
TCGA.IB.7888.01A	0.1654276	0	0.156548	0.084982	0	0.17751335	0.02454581	0	0.03393889	0	0	0	0.0675897	0.00046049	0.01870349	0.13463505	0.06131657	0	0.03206062	0	0	0.04227822

TCGA.2J.AAB9.01A	0.0440771	0	0.069974	0.130803	0	0.13994923	0.03210777	0	0.05560115	0	0	0.01620254	0.0423144	0.10248208	0.03277287	0.17564339	0.09154852	0.00280459	0.06065225	0	0	0.00306791
TCGA.2L.AAQJ.01A	0.1751658	0	0.094891	0.080965	0	0.12430441	0.00290643	0.02551559	0.02385904	0	0	0.00685	0	0.30409612	0.05823588	0.07758143	0.01620352	0	0.0094266	0	0	0
TCGA.IB.AAUU.01A	0	0.01453309	0.014938	0.028617	0	0.19506612	0	0	0.03713463	0	0	0.02363223	0.15034353	0.12232809	0.01494386	0.1463746	0.14763681	0.04078951	0.0636629	0	0	0
TCGA.Q3.A5QY.01A	0.1896801	0.0692918	0.127555	0.170115	0.025727	0.03497832	0.05736182	0.01382684	0.10793817	0	0	0	0.01192547	0.0845231	0.00391949	0.06140953	0	0.03119903	0.01054913	0	0	0
TCGA.FB.AAQ1.01A	0	0.03154767	0	0.061264	0	0.13363647	0	0.00232466	0.02922578	0	0	0.01606078	0.03709263	0.37121552	0.01532628	0.16954721	0.06471867	0.0056486	0.06076871	0	0	0.00162313
TCGA.HV.A5A3.01A	0	0.03661047	0.007574	0.029878	0	0.19527904	0	0	0.06183465	0	0.01725516	0.0135224	0	0.28360068	0.02982496	0.24466522	0.04436083	0.00260443	0.0329896	0	0	0
TCGA.IB.AAUO.01A	0	0.02619736	0.013366	0.100307	0	0.19211773	0	0.01283545	0.01934305	0	0	0.10846182	0.03128513	0.06618296	0.0493426	0.08664321	0.02216254	0.1466907	0.11737294	0	0	0.00769155
TCGA.IB.8127.01A	0.0179438	0	0.064692	0.02242	0	0.21836988	0.01668647	0	0.02639924	0.03211725	0	0.0011566	0.03753828	0.26397859	0.01550135	0.16891009	0.03128525	0.00266778	0.07988372	0	0	0.00044992
TCGA.FB.A545.01A	0.062382	0	0.10988	0	0	0.10436516	0	0.00642428	0.04806734	0.00977703	0	0.00310726	0	0.54540431	0.01937159	0.03478978	0	0	0.05643116	0	0	0
TCGA.YH.A8SY.01A	0.0044213	0.01567255	0.033812	0.032736	0	0.10233712	0	0.02482466	0.03989726	0	0	0.02032464	0	0.45029399	0	0.1714405	0.01244946	0.05592572	0.03099251	0	0	0.00487141
TCGA.IB.A5SQ.01A	0.0176349	0.04923177	0.034124	0.039609	0	0.10257988	0.00897899	0	0.03169006	0.01959928	0	0.01376477	0.00245731	0.3586518	0.00837223	0.16433279	0.05394016	0.02528411	0.06288231	0	0	0.00686577
TCGA.HV.A7OP.01A	0.2389009	0	0.328456	0.089955	0	0.07681433	0.00296252	0	0.00312071	0	0.01863844	0	0.0041176	0.05443102	0.00365441	0.11241386	0.03185512	0	0.03467969	0	0	0
TCGA.FB.AAQ6.01A	0.0439954	0.02133312	0.148283	0.025305	0	0.25187267	0	0	0.0496279	0	0	0.0504589	0	0.22340258	0.06722369	0.058302	0	0	0.05971287	0	0	0.000483

TCGA.FB.AAQ2.01A	0.1695056	0	0.078835	0.081156	0	0.09593685	0.00684387	0.02606912	0.04796974	0	0	0.01377908	0	0.28571745	0.01294059	0.11100332	0.01039956	0.01876116	0.04108277	0	0	0
TCGA.US.A77J.01A	0.3817259	0	0.209016	0.114709	0	0.03285055	0.02261904	0.00678216	0.04465465	0.01727819	0	0	0	0.06125479	0.00886751	0.06991513	0.01421107	0.00929922	0	0	0.00236066	0.00445553
TCGA.F2.7273.01A	0.0537923	0	0.048002	0.067726	0	0.28087507	0.04279747	0	0	0	0	0.00783181	0.05263447	0.01870816	0.01854848	0.21791665	0.04917932	0.01831198	0.09321832	0	0.00677368	0.02368505
TCGA.3A.A9I9.01A	0.0403208	0	0.02699	0.129912	0	0.07258207	0	0.00173444	0.0311421	0	0	0.04315335	0.02580935	0.20413452	0.04399211	0.21276068	0.08433959	0	0.05651472	0	0	0.02661477
TCGA.IB.7654.01A	0.0934616	0	0.013721	0.055	0	0.14448141	0	0	0.03487968	0	0	0.0121127	0	0.18476504	0.09895675	0.22422578	0.04132526	0	0.09640867	0	0	0.00066238
TCGA.IB.7893.01A	0.0312862	0	0.018639	0.022849	0	0.1712478	0.01050982	0	0	0	0.00737803	0	0	0.45040242	0.01359273	0.20034575	0	0.01348549	0.06026301	0	0	0
TCGA.S4.A8RM.01A	0.0182598	0	0.042136	0.098489	0	0.37662483	0.02793595	0	0	0.04404508	0	0	0	0.05591308	0.04348948	0.14582075	0.1086554	0.00476444	0.03386614	0	0	0
TCGA.IB.AAUW.01A	0.2579227	0	0.119827	0.067658	0	0.15108289	0.00566574	0.00524269	0.00590422	0.00632122	0	0.02344001	0.0078028	0	0.06293751	0.12446239	0.04887406	0	0.10184042	0	0	0.0110187
TCGA.FB.AAPZ.01A	0.0376592	0	0.040243	0.128017	0	0.09050765	0.04195281	0.02223756	0.04972085	0	0	0	0.00594105	0.32150114	0.04397618	0.12649968	0.04499556	0.00268348	0.03587064	0	0	0.00819386
TCGA.IB.AAUS.01A	0.075969	0	0.047638	0.055991	0	0.08561327	0.03493457	0	0.03082669	0	0.01469395	0	0.00859076	0.21613143	0.01164862	0.22169102	0.05348687	0.01020543	0.10832888	0	0	0.02425032
TCGA.F2.6879.01A	0	0	0.00553	0.057234	0	0.29234665	0.00895433	0.01200305	0.00048018	0	0.00390287	0	0.00560508	0.17557324	0.0911669	0.17641095	0.07168886	0.01382961	0.03197151	0.03894676	0	0.01435541
TCGA.3A.A9IS.01A	0.0557621	0	0.005858	0.081625	0	0.1007854	0.01422716	0	0	0	0	0.11030171	0.06098506	0	0	0.45921359	0	0.03115103	0	0.07751046	0.00258069	0
TCGA.L1.A7W4.01A	0.0670193	0	0.056322	0.020963	0	0.13271225	0	0.04196014	0.0141123	0	0	0.02782507	0	0.34731261	0.04007239	0.15801677	0	0.05564443	0.03199349	0	0	0.00604648

TCGA.FB.A7DR.01A	0.0762875	0	0.040503	0.088292	0	0.12942472	0.00863408	0.00498099	0.03034026	0	0	0.048386	0.00300375	0.1109219 8	0.0776844 8	0.1623075 8	0	0.06891946	0.12815862	0	0	0.02215523
TCGA.2J.AABO.01A	0.0715308	0	0.01505	0.118759	0	0.22169452	0.00474225	0	0.05396677	0	0	0.00254714	0	0.25363706	0.04663002	0.13798053	0.0052028	0.00107817	0.06718057	0	0	0
TCGA.3A.A9IH.01A	0.0151049	0.02950714	0.0725	0.070983	0	0.12756518	0	0	0.01054613	0	0	0	0.0066258	0.19778798	0.0792645	0.18348231	0.11975174	0	0.08688049	0	0	0
TCGA.PZ.A5RE.01A	0	0.04364295	0.026609	0.04974	0	0.07436992	0.0041204	0	0.047698	0	0.00167052	0.00293328	0	0.2931828	0.00703133	0.26701969	0.10206614	0.00989373	0.05657743	0	0.0031904	0.01025479
TCGA.HZ.8003.01A	0.0558262	0	0.070221	0.110684	0	0.19285369	0.02045748	0	0.01474575	0	0	0.01899794	0.01992533	0.13431049	0.08665858	0.14068626	0.02493996	0	0.09746234	0	0.00487663	0.00735508
TCGA.3A.A9IB.01A	0	0.02758697	0.001179	0.03022	0	0.16034138	0.00274335	0.0081067	0.0231082	0	0	0.01682551	0	0.26498238	0.06581134	0.269216	0.01413758	0	0.09248572	0	0	0.02325608
TCGA.H6.A45N.01A	0.03242	0.03828458	0.037573	0.111846	0	0.13996969	0	0.00940019	0.0335154	0.00212842	0	0.01476564	0	0.26168858	0.04962722	0.12296329	0.10279451	0.00210191	0.04092182	0	0	0
TCGA.HZ.8001.01A	0.1065835	0	0.180548	0.118447	0	0.05996734	0.02430244	0.01391245	0.02975039	0	0	0.02402206	0.02989821	0.08524295	0.01352644	0.1307657	0.06731571	0	0.06622641	0	0.02517206	0.02431924
TCGA.IB.A7M4.01A	0.0459524	0.01460528	0.096222	0.183975	0	0.08654212	0.00464628	0.01353029	0.0525586	0.02193468	0	0.00230175	0	0.2835528	0.04989921	0.12242017	0.00321331	0.01505025	0	0.00359587	0	0
TCGA.2J.AABP.01A	0.0115885	0	0.067288	0.109761	0	0.06731689	0.0341435	0	0.02328651	0	0.01950167	0	0	0.27470435	0.06644049	0.28906928	0.00751713	0	0.01845951	0	0	0.01092307
TCGA.HZ.A77Q.01A	0.1767631	0	0.060595	0.091228	0	0.0830317	0.02872193	0	0.04532975	0	0	0.02032418	0.03042622	0.1692734	0.00483601	0.18249176	0.01501834	0.01945989	0.04025899	0	0.01033775	0.02190424
TCGA.HZ.7926.01A	0.2867184	0	0.150684	0.124089	0	0.09352648	0.04482115	0	0.02708512	0	0.00786858	0	0.00979022	0.03382047	0.00109907	0.05288587	0.07223202	0.04805819	0.02936452	0	0.01364428	0.00431231
TCGA.XN.A8T5.01A	0.3035914	0	0.073564	0.20539	0	0.11111841	0.02069749	0	0.07169193	0	0	0.03326504	0.01824029	0.01964576	0.00098723	0.05624663	0	0.02316074	0.05477245	0	0	0.00762837

TCGA.IB.A5ST.01A	0.275873	0	0.178122	0.07049	0	0.06849153	0.01307636	0.00138707	0.05436445	0	0	0.00457537	0.00295554	0.19642515	0.00359475	0.09970063	0	0.00521768	0.01261839	0	0	0.01576861
TCGA.Z5.AAPL.01A	0.3096013	0.14518007	0.075958	0.170528	0.006677	0	0.07103864	0.05559169	0.04241136	0	0.00399698	0	0.00109082	0.0482457	0.01922375	0.02641135	0	0.02404547	0	0	0	0
TCGA.IB.7652.01A	0	0.02075043	0.028252	0.027822	0	0.10405193	0	0.00208336	0.01530663	0	0	0.02851176	0.20214871	0.15083268	0.01511831	0.23456678	0.09101436	0	0.07954179	0	0	0
TCGA.IB.7886.01A	0.0190536	0	0.038439	0.088339	0	0.20052596	0.06376466	0.00738681	0.01035752	0	0.01105757	0	0.00405932	0.10795062	0.13076465	0.20151441	0.05024525	0	0.05635847	0	0	0.01018334
TCGA.FB.AAQ0.01A	0	0.02749771	0.010592	0.017206	0	0.24484266	0	0	0.07077861	0	0	0.09294378	0	0.35333288	0.00672944	0.08801358	0	0.01215159	0	0.07559465	0	0.00031701
TCGA.IB.7897.01A	0.0642685	0	0.019839	0.147277	0	0.2931462	0.04893315	0	0	0.03601283	0	0	0.03147211	0.0110567	0.03555447	0.15979342	0.07311181	0.0233776	0.03516072	0	0.00477852	0.01621765
TCGA.2J.AABH.01A	0.1421893	0	0.124329	0.063718	0	0.19997053	0.0024634	0	0.00946583	0	0.0155686	0	0.01687687	0.20404328	0.01852938	0.12974021	0.05667548	0	0	0.00294685	0	0.01348281
TCGA.IB.AAUT.01A	0.0510018	0	0.113817	0.162167	0	0.18483661	0.00410521	0.01394267	0.03387643	0	0	0.04282017	0.01150833	0.0998779	0.04518523	0.09073355	0.08862885	0	0.05634641	0	0	0.00114291
TCGA.HV.A5A4.01A	0.0137725	0	0.062629	0.032034	0	0.06593143	0	0	0.05657165	0.06057864	0	0.0701124	0	0.378308	0.0452894	0.15117144	0.00931184	0	0.05428914	0	0	0
TCGA.US.A779.01A	0.0004964	0.07767542	0.057326	0.020889	0	0.1671695	0	0	0	0	0	0.04192647	0	0.3439222	0.01365595	0.17817785	0.01736026	0	0.0814014	0	0	0
TCGA.FB.AAPP.01A	0	0.01317497	0.020501	0.01349	0	0.3715913	0	0	0	0	0.02341095	0.1142951	0.0054001	0.029977	0.02678889	0.19077718	0.0675897	0	0.12300433	0	0	0
TCGA.HV.A5A6.01A	0	0.0024339	0.014322	0.020504	0	0.16751287	0	0	0.01835293	0	0	0.03121533	0	0.6088835	0.03328773	0.09570256	0	0.0055229	0.00226178	0	0	0
TCGA.IB.7887.01A	0.0320931	0	0.056179	0.259771	0	0.07058289	0.01543619	0	0.02100735	0	0.07611283	0	0	0.12319874	0.03025664	0.08638443	0.07559049	0	0.03647865	0.01655286	0	0.1003561

TCGA.FB.A78T.01A	0	0.01210963	0.010804	0.078858	0	0.1240189	0.01227845	0	0.0460012	0	0	0.02721127	0.12687249	0.2165746	0.04482822	0.12843151	0.12791147	0	0.04410044	0	0	0
TCGA.IB.7646.01A	0.0281131	0	0.012957	0.057177	0	0.1644438	0.06786891	0.00206854	0	0	0.01133283	0	0.00089268	0.03516572	0.10971009	0.24587556	0.03140589	0.07290459	0.05839863	0	0.01331554	0.08837066
TCGA.RL.AAAS.01A	0.1721829	0	0.06582	0.156557	0	0.0865185	0.00373999	0.02393698	0.04211766	0	0	0.01464674	0.02043312	0.04932491	0.04168009	0.05329215	0.1925942	0	0.07715599	0	0	0
TCGA.US.A77E.01A	0.0426026	0.07637198	0.031656	0.051177	0	0.14761814	0	0.01271206	0.02887625	0	0	0.03219169	0	0.30619467	0.03525382	0.17588324	0	0	0.04496366	0	0	0.01449955
TCGA.HZ.8636.01A	0.0044204	0.005862	0.018646	0.050822	0	0.19296035	0.00863144	0	0.01579886	0	0	0.00404655	0.00239638	0.17961162	0.02915604	0.26808576	0.11116295	0.02340015	0.0585339	0.01034124	0	0.0161247
TCGA.IB.AAUP.01A	0.2969082	0.01545661	0.118652	0.103214	0	0.18467588	0.01894042	0.01443984	0.03769995	0	0	0.01308193	0	0.08088685	0.0134316	0.0662123	0	0.00996091	0.02643971	0	0	0
TCGA.IB.7885.01A	0.0171133	0.02078354	0.094952	0.084895	0	0.15331167	0.00250413	0.02437336	0.0439201	0	0	0.02334024	0.00586004	0.13976288	0.05478186	0.15064722	0.09183625	0	0.04517318	0	0	0.04674584
TCGA.IB.AAUR.01A	0.3890461	0	0.074395	0.106421	0	0.14224576	0.02227684	0.0071884	0.06157464	0	0	0.01091929	0.00557601	0.07428783	0	0.04248447	0.00165561	0.0213983	0.03722922	0	0	0.00330156
TCGA.IB.7889.01A	0.0449372	0.05264484	0.057515	0.136621	0	0.10934881	0.0183422	0	0.01475797	0	0	0.00678856	0.03745958	0.2510575	0.04224822	0.1148907	0.04888333	0	0.06112004	0	0	0.00338431
TCGA.F2.A8YN.01A	0	0.04195862	0.033922	0.047238	0	0.1286592	0	0	0.03223161	0	0	0.03386173	0.05826135	0.26723487	0.0009514	0.16052228	0.10003737	0.01606084	0.0568659	0	0	0.02219469
TCGA.3E.AAAZ.01A	0.0357271	0	0.123287	0.104602	0	0.21142858	0	0.01386449	0.03182997	0	0	0.009463	0.01521583	0.1344799	0.01603011	0.21687607	0.0274133	0.00912431	0.05065827	0	0	0
TCGA.HZ.8637.01A	0.0544564	0.00743357	0.056991	0.093593	0	0.09649478	0.0450221	0	0.02149995	0	0	0.03544181	0	0.15574603	0.16096086	0.11287019	0.06297824	0	0.07632054	0	0	0.020192
TCGA.HZ.A4BH.01A	0.0478459	0.05857255	0.023669	0.122021	0	0.1363234	0.01683008	0.0049288	0.03173831	0	0	0.03666291	0.01595689	0.19903824	0.09513239	0.13587726	0.0151103	0	0.05982593	0	0	0.00046763

TCGA.3A.A9I5.01A	0.1637721	0	0.159758	0.058275	0	0.09045769	0.00232634	0.03444023	0.04395829	0	0	0.00311733	0	0.25960706	0.00946217	0.12055068	0.00185973	0.0010796	0.0259308	0	0	0.02540492
TCGA.XD.AAUL.01A	0	0.00998882	0.02049	0.034539	0	0.18631584	0.00931085	0	0.04650309	0	0	0.00996707	0.00076347	0.12490755	0.02098617	0.30211707	0.11252165	0.01486886	0.05338736	0	0	0.05333285
TCGA.HZ.7923.01A	0.0305977	0.00208217	0.073103	0.156656	0	0.25945307	0.02110849	0	0.01947325	0	0	0	0.03544279	0	0.00300884	0.18283697	0.07402249	0.05152077	0.06109932	0.00405461	0	0.02554107
TCGA.2J.AABE.01A	0	0.05624391	0.032125	0.031087	0	0.13675463	0	0.0077516	0.02024465	0	0	0.00579867	0.08308568	0.19668249	0.00201836	0.25381615	0.06607908	0.02254669	0.03913267	0	0.01050861	0.03612532
TCGA.FB.AAPU.01A	0.1758133	0	0.122708	0.081562	0	0.22512316	0.00308001	0.01611601	0.07193123	0	0	0	0.00377414	0.00499841	0.00955829	0.07306519	0.15702325	0.04050069	0.01474729	0	0	0
TCGA.3A.A9IC.01A	0.1588253	0	0.095921	0.073433	0	0.15460668	0.02901796	0.01200584	0.01785258	0	0	0.0231053	0.00344029	0.14716337	0.01939791	0.13808943	0.04190776	0	0.05521626	0	0	0.03001739
TCGA.2J.AABI.01A	0.2450158	0	0.104934	0.028835	0	0.07545857	0	0.01679481	0.04584144	0	0	0.02068067	0	0.32399473	0.00352894	0.09019946	0	0.032117	0.0116334	0	0	0.00096665
TCGA.HZ.A77O.01A	0	0.00302586	0.10815	0.075902	0	0.05572763	0.03313792	0	0.01903307	0	0	0.01605624	0.00326454	0.25272082	0.08668696	0.20758855	0.05346004	0	0.06717298	0	0	0.01807351
TCGA.YY.A8LH.01A	0.0316883	0.02756752	0.054831	0.109849	0	0.21372149	0.000806	0	0.0752886	0	0	0.08421579	0.00563362	0.09972161	0.02991623	0.08471245	0.05631137	0.02425772	0.10147904	0	0	0
TCGA.3E.AAAY.01A	0.03012	0.00843865	0.04249	0.104878	0	0.17307017	0.01713104	0	0.02908638	0	0	0.01309211	0.00710535	0.08247159	0.06092916	0.28144157	0.05137364	0	0.05277637	0	0	0.04559564
TCGA.3A.A9IZ.01A	0.0107284	0.03815959	0.016451	0.042709	0	0.25577838	0.01072786	0.04152661	0	0	0.00038725	0.052609	0.00063367	0.22830029	0.08567477	0.0847598	0	0	0.03894479	0	0.01474399	0.07786513
TCGA.IB.A6UG.01A	0.1721547	0	0.164696	0.141762	0	0.11289218	0.02564149	0.02661922	0.04966072	0	0	0.01574178	0.01024763	0.06273693	0.02260175	0.06710764	0.05710024	0.01927977	0.05175787	0	0	0
TCGA.FB.A5VM.01A	0.0169157	0.04656245	0.033384	0.100652	0	0.07983124	0.04089242	0.0029038	0.04969389	0	0.00497734	0	0.00777469	0.31321032	0.03025651	0.13264506	0.06678338	0.01500566	0.05851095	0	0	0

TCGA.RB.AA9M.01A	0.0600995	0	0.087928	0.119696	0	0.20851966	0.00292646	0.02444013	0.0308076	0	0	0	0.05918649	0.11425144	0.04778084	0.1099464	0.10123026	0	0.02721973	0	0	0.0059675
TCGA.HZ.A49G.01A	0.0371777	0	0.034015	0.066021	0	0.11062066	0.01480176	0.00725805	0.05413213	0	0	0.00058935	0.04565858	0.41389862	0.01868203	0.14004639	0.01300898	0	0.04408973	0	0	0
TCGA.HV.AA8X.01A	0.0106768	0.02957474	0.131304	0.025949	0	0.10111737	0	0	0.01111255	0	0	0.04115561	0	0.13003841	0.01147911	0.17340397	0.01165927	0.0861215	0.2353866	0	0	0.00102121
TCGA.LB.A7SX.01A	0	0.03162159	0.349981	0.096544	0	0.14639454	0	0.03470256	0.03514744	0	0	0.05207161	0	0.08812559	0.0104324	0.06232614	0	0.00683827	0.03377577	0	0.03658353	0.01545584
TCGA.HZ.7922.01A	0.027261	0.02355663	0.049349	0.066054	0	0.20617439	0.00652646	0.01503593	0.03072586	0	0	0.03661413	0.05369706	0.03578998	0.08186629	0.11101736	0.1227226	0.00866917	0.08831304	0	0	0.03662749
TCGA.2J.AABV.01A	0.0037535	0	0.114916	0.287211	0	0.06140129	0.03196918	0.00171977	0.04214597	0	0	0.03810067	0.10822148	0.00843875	0.01902668	0.10823245	0.08141595	0.00266154	0.09078553	0	0	0
TCGA.IB.AAUN.01A	0.1673729	0	0.188439	0.077672	0	0.059138	0.06506313	0	0.00457713	0	0	0.03367678	0.00796801	0.05350867	0.09805182	0.0927367	0.05056816	0.01308538	0.08314725	0	0.00499535	0
TCGA.2L.AAQA.01A	0.0114485	0	0.043536	0.038076	0	0.14692797	0	0	0.01327002	0	0	0.01635216	0	0.48900712	0.03109952	0.14105962	0.03069694	0	0	0.03852586	0	0
TCGA.IB.A6UF.01A	0	0.03398864	0.038728	0.047273	0	0.10628993	0.00362436	0	0.0047099	0	0	0.03754463	0.13887998	0.18969763	0.03894231	0.15949326	0.04222745	0	0.12720479	0	0.03001414	0.00138165
TCGA.HV.A7OL.01A	0.2804091	0	0.157862	0.086011	0	0.02631271	0	0.08197533	0.03290435	0	0	0.04056014	0	0.09410383	0.03555421	0.07530725	0.00776266	0.01052259	0.05492428	0	0	0.01579071
TCGA.IB.7651.01A	0.0049115	0.00132702	0.149801	0.025404	0	0.25342716	0.04087313	0.00206208	0.02097549	0	0.00702505	0	0	0.16744148	0.09868634	0.12142383	0.02988665	0	0.07477952	0	0	0.00197578
TCGA.2J.AABU.01A	0.0375721	0.04639049	0.031808	0.12791	0	0.20831757	0.00327836	0	0	0	0	0.0297417	0.014709	0.25487847	0.0150161	0.14750684	0	0.01979248	0.06307846	0	0	0
TCGA.XN.A8T3.01A	0.0429751	0	0.018623	0.214177	0	0.1025821	0.01514323	0	0.00098367	0.01790369	0	0.00185215	0	0.14467553	0.09568325	0.22415338	0.07717496	0	0.04407277	0	0	0

TCGA.HV.AA8V.01A	0.022808	0	0.083361	0.064454	0	0.14585692	0	0	0.04318582	0	0.00418508	0.01398752	0	0.28423706	0.01915127	0.17355534	0.0767047	0.00597671	0.05311952	0	0	0.00941715
TCGA.S4.A8RO.01A	0.0076372	0.02527199	0.015606	0.040191	0	0.07873049	0.00878054	0	0.00827117	0.00025472	0	0.0072081	0	0.40305108	0.02450993	0.27658561	0	0.03812677	0.06577572	0	0	0
TCGA.IB.8126.01A	0.1017015	0	0.013256	0.212533	0	0.22453749	0.02663088	0.01243417	0.00543478	0.06950966	0	0	0.00661464	0.02742035	0.05581821	0.13332924	0.01864541	0.00970475	0.06981902	0	0	0.01261092
TCGA.2J.AABF.01A	0.0499771	0	0.021558	0.09331	0	0.12308094	0.0147728	0	0.03732482	0	0	0.03809473	0.0233541	0.05028303	0.12747203	0.20908097	0.09302338	0	0.07676868	0	0	0.04189885
TCGA.US.A776.01A	0.1036447	0	0.235625	0.050341	0	0.23910589	0	0	0	0	0	0.0909733	0.00263017	0.06187728	0.02643378	0.10940392	0	0	0.07996552	0	0	0
TCGA.2J.AABT.01A	0.0378174	0	0	0.073144	0	0.20078553	0.03232581	0	0.05786429	0	0.00200061	0	0.05405682	0.14701448	0.03404489	0.23880372	0.04466375	0.02816213	0.03934433	0	0.00265059	0.00732192
TCGA.IB.7891.01A	0.1110808	0	0.18222	0.080778	0	0.14912428	0.02368708	0	0.02712021	0	0	0.01232068	0.01282817	0.06704852	0.07471894	0.12081236	0.04019962	0	0.08968823	0	0	0.00837315
TCGA.XD.AAUI.01A	0.2130612	0	0.101732	0.079834	0	0.05575446	0.01848406	0.01207285	0.06509675	0	0	0	0.00224864	0.22512277	0.02276639	0.10524116	0.04771724	0.01087103	0.03919908	0	0	0.0007978
TCGA.RB.A7B8.01A	0	0.02542234	0.050409	0.104381	0	0.245617	0	0.00294314	0.06065333	0	0	0.05214366	0.01334273	0.13995356	0.05074499	0.15369633	0.04418133	0	0.03352552	0	0	0.02298566
TCGA.XD.AAUH.01A	0.1755906	0.05545804	0.100632	0.145466	0	0.1898709	0.01080476	0	0.05766393	0	0	0.01879003	0.00682503	0.02984437	0.01606691	0.12691561	0.02984157	0.0006653	0.03086323	0	0	0.00470103
TCGA.2J.AAB6.01A	0	0.04498469	0.008997	0.024565	0	0.11702499	0.00716497	0.01176849	0.00401579	0	0.01383871	0	0.00200479	0.21905338	0.04839063	0.40851326	0.01305405	0.00483489	0.05638315	0	0.0085597	0.00684615
TCGA.2J.AAB4.01A	0	0.00520238	0.001535	0.125291	0	0.18569168	0.02668529	0	0.01861823	0	0	0.03261129	0.03494845	0.09507921	0.11951839	0.14242612	0.13368925	0.00529108	0.0734125	0	0	0
TCGA.FB.A4P6.01A	0.0352696	0	0.014879	0.176452	0	0.24546546	0	0	0.03823592	0	0	0.01829013	0.08212671	0	0.02086367	0.18110284	0.05343649	0.02118113	0.11269753	0	0	0

TCGA.IB.AAUV.01A	0.0741561	0.02630941	0.03683	0.182561	0	0.20338237	0.01798389	0.00460603	0	0.01249216	0	0.01363368	0.03836703	0.05047809	0.01894658	0.18762925	0.0331887	0	0.08842016	0	0	0.01101546
TCGA.3A.A9IL.01A	0.0344984	0.00082298	0	0.087006	0	0.25197837	0	0	0.03437184	0	0	0.05514348	0.06397264	0	0.01340759	0.22975696	0.08747047	0.02482556	0.11674544	0	0	0
TCGA.IB.AAUQ.01A	0	0.04281602	0.137382	0.005206	0	0.08124407	0	0	0.02869723	0.01795179	0	0.0030689	0	0.44102495	0.04298349	0.13762839	0	0	0.06199661	0	0	0
TCGA.FB.AAPQ.01A	0.0003026	0.03627503	0.037096	0	0	0.02414443	0.00931503	0.01786817	0.03407278	0.03185435	0.08094595	0	0	0.58461656	0.02551128	0.01561829	0	0	0	0.10238002	0	0
TCGA.IB.A5SS.01A	0.0193855	0	0	0.037179	0	0.1160962	0.01816228	0	0.01136941	0	0.00876096	0.00573707	0.02217455	0.20593881	0.01684713	0.43664794	0.00270179	0.02064009	0.05818046	0	0	0.02017872
TCGA.IB.7645.01A	0.0619483	0	0.008682	0.125552	0	0.23054887	0.05349294	0	0.01827016	0	0	0	0.02505647	0.14280774	0.04826002	0.17969116	0.06685927	0	0.02200482	0	0	0.01682662
TCGA.2J.AAB1.01A	0.0359236	0	0.057881	0.100437	0	0.30333743	0.04062074	0	0.00162936	0	0	0	0.02563884	0.03213428	0.12560281	0.111507	0.0564319	0.04269888	0.06615777	0	0	0
TCGA.IB.A5SP.01A	0.002304	0.0222983	0.022661	0.037677	0	0.20996681	0	0	0.03252205	0	0.01603112	0.02303981	0	0.20686296	0.03841349	0.21759543	0.06572228	0.00764363	0.09726202	0	0	0
TCGA.OE.A75W.01A	0.074579	0	0.039811	0.10581	0	0.20479306	0.04479358	0	0.03852798	0	0	0.01907194	0	0.22691427	0.09118703	0.09945654	0.00575903	0	0.04692321	0	0	0.0023729
TCGA.F2.7276.01A	0.0986194	0	0.043323	0.088835	0	0.15300817	0.01640582	0	0.01159958	0.01602064	0.01871931	0	0	0.28421796	0.06170207	0.14939064	0	0	0.03096847	0.00441553	0	0.02277405
TCGA.3A.A9IX.01A	0.1202424	0	0.063756	0.142699	0	0.14479972	0.00551474	0.00818747	0.06023497	0	0	0.00505421	0.0684484	0.0246019	0.01362506	0.09984644	0.14408782	0.0492392	0.04471832	0	0	0.00494422
TCGA.3A.A9IV.01A	0.0040007	0	9.55E-05	0	0	0.28054345	0	0.01122614	0.00115375	0	0.00585425	0.09390019	0.01442102	0.35372694	0	0.18792933	0	0	0.04520505	0	0.00194372	0
TCGA.HZ.8315.01A	0.075254	0	0.079182	0.092317	0	0.1295228	0.04278751	0.0052777	0.01433241	0	0	0	0.00032518	0.11087494	0.14895524	0.14227216	0.06760904	0	0.0846359	0	0	0.00665418

TCGA.Q3.AA2A.01A	0	0.05918602	0.018059	0.01318	0	0.11336048	0	0	0.01753672	0	0.02185997	0.00130535	0	0.45437059	0.01718197	0.2213483	0.02348491	0	0.03912605	0	0	0
TCGA.HZ.7925.01A	0.0197916	0.00403486	0.013088	0.026116	0	0.17213171	0	0.00058994	0.02855572	0	0	0.02451709	0.00035851	0.24982497	0.0237378	0.25425038	0.00525007	0.03756164	0.05414131	0	0.03097413	0.05507596
TCGA.HZ.7289.01A	0.0107097	0	0	0	0	0.36411085	0	0	0.07107742	0.01507495	0	0.06915914	0	0.3717911	0	0.02546723	0	0.01420178	0.05584119	0	0.00256668	0
TCGA.HZ.8519.01A	0.061386	0	0.002803	0.200835	0	0.19399824	0.00368257	0.00046421	0.01115732	0	0	0.01906836	0.01876176	0.11233645	0.06827005	0.14758147	0.08317968	0	0.07647573	0	0	0
TCGA.F2.A44G.01A	0.0001098	0.01781645	0.001257	0.058795	0	0.23102412	0	0.00092853	0.01973036	0	0	0.04248754	0.01362925	0.18948633	0.0796208	0.15486023	0.06970248	0.00221593	0.07127593	0	0.00209995	0.04495997
TCGA.HZ.8638.01A	0.1371164	0	0.242852	0.17484	0	0.04752092	0.07842097	0	0.00359957	0.00259862	0.0088619	0	0.00355772	0	0.06483945	0.06043942	0.04110018	0.00323998	0.07320454	0	0	0.05780827
TCGA.3A.A9IU.01A	0.0240761	0.02223632	0.085922	0.028388	0	0.04635961	0	0.01174537	0.02251005	0	0	0.02984628	0	0.55052279	0.04736512	0.07695464	0	0	0.05407315	0	0	0
TCGA.2J.AABR.01A	0.051732	0	0.047346	0.191909	0	0.25176703	0.02467129	0	0.02166077	0	0	0.0296623	0.02419616	0.06351009	0.05768731	0.10617752	0.07412757	0.00177619	0.04616013	0	0	0.00761708
TCGA.2J.AABA.01A	0.1107829	0	0.187202	0.125893	0	0.24736198	0.0211539	0	0.04813127	0	0.00328564	0	0	0.10521224	0.01153264	0.08919366	0.00685918	0.01327319	0.02472163	0.0053972	0	0
TCGA.F2.6880.01A	0.0410156	0	0.021281	0.142041	0	0.10305539	0	0.01119539	0	0	0	0.07841607	0.01740826	0.11435715	0.04005304	0.13878918	0.03240452	0	0.2599833	0	0	0
TCGA.F2.A7TX.01A	0.0524517	0	0.034247	0.162776	0	0.17590229	0.09549704	0.0238727	0.00310866	0	0	0.03146066	0.00477296	0.06553231	0.12169522	0.14317395	0.00370095	0.00666224	0.0711581	0	0	0.00398811
TCGA.HZ.A4BK.01A	0.0153139	0	0.048029	0.126005	0	0.12164894	0.00758319	0	0.0574562	0	0	0.0086161	0.0581361	0.29545877	0.03213419	0.09780187	0.04757249	0	0.0842434	0	0	0
TCGA.HZ.A77P.01A	0.0280129	0	0.01053	0.135523	0	0.15178438	0.01285915	0	0.02402062	0	0.01021056	0	0.03382098	0.05001203	0.03592224	0.30825159	0.07726538	0.01087379	0.11091332	0	0	0

TCGA.3A.A9J0.01A	0.0205119	0.01030762	0.185305	0	0	0.0485304	0	0.02022784	0.01577667	0.01738907	0.00119738	0.01071847	0	0.45912535	0.02848511	0.16365566	0	0	0	0.01595204	0	0.00281795
TCGA.HZ.A8P1.01A	0	0.04951122	0	0.062866	0	0.27178919	0	0	0.03087384	0	0.03690153	0	0	0.07719747	0.04081878	0.30920377	0.04592777	0	0.07439031	0	0	0.00052033
TCGA.2L.AAQL.01A	0	0.02496404	0.025643	0.152567	0	0.12887857	0.01604088	0	0.02994132	0	0	0.01890433	0.01607849	0.1963021	0.06292306	0.17170494	0.07107095	0	0.07703876	0	0	0.00794244
TCGA.HZ.8317.01A	0.0132186	0	0.006122	0.075225	0	0.29771416	0	0	0.00117931	0	0	0	0.01986462	0.12217611	0.01930127	0.23117576	0.02640112	0.01330212	0.04953903	0.09298054	0	0.03180022
TCGA.HZ.7920.01A	0.1472266	0	0.060019	0.142724	0	0.22926752	0.05350631	0	0	0	0	0	0.05079569	0	0.03429736	0.1304006	0.07371879	0	0.07349643	0	0	0.00454865
TCGA.HZ.A49I.01A	0.0745257	0	0.017801	0.045988	0	0.12115539	0.01611533	0.00774857	0.02307754	0	0	0.00312436	0.33245649	0.1693824	0.00443808	0.10001966	0.00424317	0.02884838	0.0510759	0	0	0
TCGA.HZ.A9TJ.01A	0.0851201	0	0.092535	0.055702	0	0.22319818	0.00188931	0	0.00804658	0	0	0.00488497	0.01192912	0.16091921	0.07044298	0.16644548	0.00819882	0.01778311	0.09290448	0	0	0
TCGA.YB.A89D.01A	0	0.02317645	0.018764	0.057706	0	0.17302821	0	0.00775687	0.05206855	0	0	0.01421436	0.03598716	0.08546793	0.01425041	0.2702377	0.1124816	0.08346869	0.00322704	0.03899279	0	0.00917251
TCGA.IB.7649.01A	0.0151025	0	0.076472	0.103577	0	0.15592577	0.03527963	0	0.03417817	0	0	0.04213103	0.1163763	0.0691162	0.01620745	0.08543283	0.07568546	0.01034923	0.15910037	0	0	0.00506553
TCGA.LB.A8F3.01A	0	0.03629511	0.04364	0.051411	0	0.14547623	0	0.01080225	0.02338264	0	0	0.03789296	0.0132063	0.2162234	0.06205412	0.2440024	0.0214425	0	0.04951006	0	0	0.04466064
TCGA.2J.AAB8.01A	0.0676133	0.0082553	0.055999	0.09105	0	0.14311865	0.00217071	0.03573901	0.05425336	0	0	0.01414358	0	0.09626295	0.03297893	0.20842396	0.11466068	0.03002729	0.03719782	0	0	0.00810637
TCGA.3A.A9IR.01A	0	0.02450459	0.023693	0.167536	0	0.25079039	0	0	0.00672178	0.00434057	0	0.04125787	0.0216897	0.06270496	0.07047023	0.08545757	0.01422631	0	0.21689419	0	0	0.00971253
TCGA.2L.AAQE.01A	0.0688929	0.11356725	0.029983	0.162741	0	0.11254612	0	0	0.13546042	0	0	0.05060673	0	0.22018093	0.01022457	0.06025745	0	0.00463168	0.03090736	0	0	0

TCGA.3A.A9I7.01A	0.0730987	0.01637469	0.117234	0.143017	0	0.17666133	0	0.01162942	0.07509345	0	0	0.02557587	0.00561054	0.07078172	0.05434194	0.11462775	0.05626843	0	0.05968547	0	0	0
TCGA.IB.7890.01A	0.0037451	0.00990129	0.070579	0.021205	0	0.08361021	0.01482998	0	0.03459224	0	0.00666684	0	0	0.40331838	0	0.227396	0.03311843	0	0.08568466	0	0	0.00535129
TCGA.LB.A9Q5.01A	0	0.01616161	0.00632	0.060215	0	0.14149203	0	0.00772722	0.02104026	0	0	0.03814891	0.03497571	0.1884778	0.03795133	0.17223017	0.10513794	0.00813713	0.07045573	0	0	0.09152991
TCGA.HZ.7924.01A	0.0510156	0	0.111836	0.074337	0	0.27606717	0.00375798	0	0	0	0.00149832	0.00771223	0.00765914	0	0.01417404	0.1789251	0.04402902	0.01980324	0.17649423	0	0.02057778	0.01211259
TCGA.HZ.8002.01A	0.0443114	0	0.019366	0.143666	0	0.19654647	0.01462883	0	0.00318989	0	0	0.0110327	0.0784132	0.12181425	0.02289624	0.19308291	0.05854557	0.01298649	0.06994623	0	0	0.00957439
TCGA.FB.AAPY.01A	0.0783292	0.06288486	0.060964	0.084617	0	0.13602584	0.00172267	0.03711728	0.08158522	0	0	0.00889523	0.00247686	0.07498701	0.023345	0.16392849	0.07331391	0.03684828	0.06032653	0	0	0.01263294
TCGA.F2.A44H.01A	0.0370287	0	0.011752	0.027032	0	0.08648702	0	0	0.01269961	0	0.00256387	0	0.03490154	0.51854969	0.01784972	0.16570116	0.03976773	4.95E-05	0.03672386	0	0	0.00889383
TCGA.IB.A5SO.01A	0.0567301	0.06032013	0.06583	0.127365	0	0.12251797	0.00673159	0.00882336	0.03754445	0	0	0.00029186	0	0.27286763	0.02548957	0.12548345	0.03773879	0.01908483	0.0331819	0	0	0
TCGA.FB.AAQ3.01A	0.0024703	0	0.004191	0.040711	0	0.18760253	0.01682092	0.0003372	0.02995356	0.04615665	0	0	0.02012727	0.13027827	0.12415014	0.3000559	0.04222435	0.01109505	0.03247367	0	0.01066377	0.00068798
TCGA.HZ.7918.01A	0.0208589	0	0.087125	0.145809	0	0.26017943	0.02738777	0	0.01029047	0.00784036	0	0.00080002	0.00311754	0.07240121	0.12170861	0.09780685	0.06431344	0	0.07011164	0	0	0.01024933
TCGA.H8.A6C1.01A	0.0126546	0.09731139	0.032342	0.067985	0	0.13594808	0	0	0.08358876	0	0	0.02537539	0.00086127	0.03463729	0.01490651	0.16613073	0.1538106	0.06551121	0.07455573	0	0	0.03438218

Supplementary Table 13: Enrichment of 28 immune cell types by ssGSEA algorithm in TCGA_PAAD cohort.

TCGA_ID	Activated B cell	Activated CD4 T cell	Activated CD8 T cell	Activated dendritic cell	CD56bright natural killer cell	CD56dim natural killer cell	Central memory CD4 T cell	Central memory CD8 T cell	Effector memeory CD4 T cell	Effector memeory CD8 T cell	Eosinophil	Gamma delta T cell	Immature B cell	Immature dendritic cell	Macrophage	Mast cell	MDSC	Memory B cell	Monocyte	Natural killer cell	Natural killer T cell	Neutrophil	Plasmacytoid dendritic cell	Regulatory T cell	T follicular helper cell	Type 1 T helper cell	Type 17 T helper cell	Type 2 T helper cell
TCGA.2J.AAB1.01A	-0.748865	0.869113	1.545942	-0.218801	0.1135516	0.452645	0.251325	-0.670009	-1.762861674	1.168743683	-0.5810955	-1.012895866	-0.347243509	-0.715981867	-0.229613096	-0.254715645	0.011695246	-1.427723685	1.636688341	-0.648010601	-0.190662242	-1.655269654	-0.951136326	-0.117433283	0.205559017	1.369088499	1.378813572	1.023003
TCGA.2J.AAB8.01A	-0.0871	0.548182	-0.81953	-1.87635	0.824878	0.346834	0.786745	1.450299	0.384783826	-1.7101968	-0.428125	1.756742755	0.237380322	-0.670937224	-0.490683443	-0.24990222	-1.026478073	0.534191732	-0.301273197	1.165943615	0.226985854	-0.114080038	-0.233416675	-0.020667348	-0.679593891	1.248150369	-0.757880106	1.634658
TCGA.2J.AABA.01A	0.346355	0.370101	0.106865	-0.79015	0.376523	0.733968	-0.37406	0.295016	0.434755157	-0.585713274	-0.421876	0.519436196	0.010298192	-0.44298036	-0.961951803	0.401548314	-0.979858929	1.51602329	0.003859654	0.679303504	-0.922973554	-0.279593504	0.915836954	-0.816399888	-2	1.744165035	-0.0987998	1.207011
TCGA.2J.AABF.01A	0.155491	1.139727	0.11995	0.734642	-1.30468	-0.16296	-1.4452	0.312074	0.651793194	0.772590279	-1.765538	-0.461981134	0.478942573	-0.536204041	1.056860942	0.517594761	1.504692656	-1.445129757	-0.237545403	-0.402289581	-1.058738928	0.899754572	-1.575280065	1.549596613	1.11167917	-0.801024974	-0.663279944	-1.1059
TCGA.2J.AABO.01A	0.238975	-1.26	0.079467	-0.36	0.93922	0.295962	1.181391	-0.13	0.873819675	-1.5987	-1.528	0.40619582	-0.3300	0.026374249	-1.0108	0.372559143	-1.3658	0.009971142	0.108394251	1.283033992	0.29210175	-0.0857	0.484992113	-0.6360	-0.0819	1.840699893	0.17793608	0.640751

		344		839				887		6898	829		11038		29094		19775					99929		30454	95211			
TCGA.2J.AABP.01A	-0.12816	1.646892	0.963883	-1.52003	-1.25506	-0.27431	0.86856	0.463636	0.216635624	0.983654916	-0.390543	1.459778068	-0.321568727	1.92574945	1.636189951	-0.567757373	1.426791135	-0.188551859	-0.803855264	-0.147328532	-0.645022532	-1.16299223	0.777195124	0.985649722	1.701401051	-1.928727411	-2	-0.14851
TCGA.2J.AABR.01A	0.830537	1.35588	1.024454	-0.46016	-0.83152	-1.7194	-0.40931	-1.88849	-1.184420559	1.062941146	0.3124901	-2	0.258114613	0.088232984	0.986436277	0.542030637	0.775105268	-1.352820148	0.038587829	-0.398822416	-0.370571249	-0.572480327	-0.781985006	0.49227059	-0.273521634	1.214991017	-0.233998609	-0.112244
TCGA.2J.AABU.01A	0.41621	0.706518	0.917747	-0.16557	-0.14419	0.070026	0.672704	-0.92279	-0.387159923	-0.321097985	-0.012775	0.64629063	0.190985325	0.30245295	-2	-0.676615915	-1.325216548	0.524944948	-1.234798021	0.671195448	0.601568436	-0.162251141	0.452391785	-0.83323138	-0.558441214	0.647945488	0.370993733	1.4994
TCGA.2L.AAQA.01A	-1.42268	1.308402	-0.51042	0.675211	0.509096	1.07323	0.831119	0.523371	-1.305863084	-0.561309827	0.6670434	0.855235423	-1.411984524	-0.372261072	-1.220026841	-0.432559345	-1.179573067	1.859480792	-0.166222554	-1.006382993	2	0.868317805	1.168384698	-0.339441208	-1.148982845	-0.621852917	1.013739863	1.12387
TCGA.2L.AAQE.01A	0.757532	0.630503	1.376867	-0.55916	0.811012	-0.52007	0.948977	-0.63789	-0.886235677	0.838863998	-0.644815	0.150332708	0.967563735	-0.27620693	-1.444344846	-0.776421681	-1.029529728	0.048360627	1.386260795	0.816159809	-0.477217854	-0.981931871	0.833971131	-1.487133514	-2	-0.126601676	-0.04599972	0.577358
TCGA.2L.AAQI.01A	-0.50084	0.552446	-0.77634	0.732548	1.564233	-0.76865	0.693263	1.825737	0.862273867	-0.214071338	0.6074332	0.476832053	-1.337172715	1.401267862	-0.877055171	-1.040109759	-1.46162492	0.642429819	0.458938398	-0.2943502	0.850286785	-0.430000135	0.288069569	-1.687431315	-0.998956866	0.073811926	1.340847984	0.541116
TCGA.2L.AAQJ.01A	1.387697	0.230333	0.98647	0.124919	-0.40609	1.500369	1.324025	0.253883	-1.875053543	-1.103840152	0.2424186	-1.014309739	1.002332596	-1.606610563	-1.575185854	-0.500488427	-0.725260333	-0.159775697	1.134649055	-0.638670688	-1.097483849	-0.330388584	0.257331604	-0.524890198	-0.343874129	0.943808274	0.25781868	-0.18225
TCGA.2L.AAQL.01A	-0.65	-1.37	0.512214	0.666642	0.782367	0.607549	0.791538	0.643596	-1.0550	1.411030299	-0.342	0.610874166	-0.7430	-0.6323	0.876260145	0.034033683	0.08122508	-1.1788	1.597783974	1.304919556	-0.0137	-1.0727	-0.4019	-0.3670	0.251942722	0.229396024	0.773469619	-1.73

	187	117							60719		675		0907	12743				52155			41904	03322	19465	46795				517
TCGA.3A.A9I7.01A	1.68224	-0.35814	0.741774	-1.22932	-1.27742	-0.09072	-0.2799	-0.21039	0.584090935	0.306853682	-0.591392	1.163260396	1.614588404	-1.817569284	-0.527888473	-0.393735378	-0.379671712	-1.160614734	0.443499575	0.699189102	-0.97132972	-0.167740821	-1.280155481	-0.253272805	-0.159314786	1.814918843	0.02101526	-0.79668
TCGA.3A.A9IB.01A	-1.80158	-0.13511	-1.47116	0.087945	0.305354	0.86188	1.207895	0.560406	-1.541825125	-0.447930597	-0.671308	2	-1.440828654	0.109342098	0.574474683	1.283390537	0.434065441	-0.631273052	-0.698182069	1.292355617	0.127907426	-1.142539437	0.023616424	1.308969879	1.273449512	0.170322782	-0.176286604	0.655363
TCGA.3A.A9IH.01A	-0.67022	0.150678	-0.66148	1.343564	0.942938	0.606522	1.670045	-0.46217	0.245049541	0.191271193	-0.606497	0.347811565	-1.122972208	1.065444622	-1.099486937	-1.764972868	-1.416119776	-0.322416862	1.525357029	0.747924213	0.125417534	0.024265483	0.578379346	-1.077542317	-0.432156177	-0.241492126	0.921489948	1.535445
TCGA.3A.A9IU.01A	0.487407	-0.95032	-1.38185	1.923786	1.115219	1.597363	-0.67494	0.0894	0.385915448	-1.42979458	0.082967	-0.317438327	0.718027481	0.035544984	-0.513689612	-0.739067842	-0.819854807	0.220327418	0.404898227	1.471189011	-0.301862033	-0.14860223	1.33887147	-0.811326172	-0.498905348	-1.296072089	0.75938567	0.328178
TCGA.3A.A9IX.01A	1.622305	0.47331	1.021664	-2	-0.78622	-2	-0.58637	-1.92425	0.482420555	0.441053708	0.3621099	-1.232068977	1.434935309	-1.087541264	0.253618557	1.590088809	0.651341905	-0.547713617	-0.901751649	-0.627170087	-1.630368945	-1.661807252	-0.952054881	0.910201487	0.889335462	1.485185634	-0.534249524	0.146884
TCGA.3A.A9IZ.01A	-1.16998	1.09198	-0.47216	1.300947	0.71387	1.027662	-0.29675	0.831654	0.575485733	-0.868769459	-0.179698	-0.341524245	-1.560434623	0.717941488	-1.439489103	-0.639754917	-1.133103822	0.002010766	-0.056547296	-0.217910509	1.448402606	0.990140768	0.631885343	-1.33611969	0.061224632	-0.315683172	1.263174743	1.861561
TCGA.F2.6879.01A	-1.81759	0.31867	-1.13295	0.699876	0.507804	0.404413	1.830185	1.416819	1.221996863	-1.17938375	-0.767043	0.917724596	-0.914868637	0.452979966	-1.730807138	0.691137211	-0.788312415	2	0.547715083	0.923472075	-0.100404	0.356604415	-0.283941512	0.376256509	-1.035759035	-0.688088501	-0.048325292	2
TCGA.F2.7273.01A	-0.0619	-0.17	-0.43	-0.06	-1.5543	-1.75	-0.09	-0.35	0.434887306	0.865472689	0.4559699	-2	0.084283911	0.865945953	0.622444445	0.788783556	1.06420539	0.527495412	-0.2330	0.531824579	0.189194452	0.173173087	-0.5145	1.069806194	2	0.407086627	-0.6492	-0.62

		151	163	812		424	614	818											63471				24698				13813	419
TCGA.F2.7276.01A	1.272241	0.279484	0.186519	-0.47803	-1.8328	-2	-0.44992	-0.77257	0.446846613	0.683967274	0.4266305	-1.343165531	1.315338035	0.015277291	-0.343380192	1.554478062	1.031385429	-0.811376	-0.596511454	-0.236404163	0.107940224	-0.017178232	-0.765255368	0.844326108	0.342952963	0.763816386	-1.068346041	-1.23736
TCGA.F2.A7TX.01A	0.04004	2	1.841822	-0.08845	-0.2641	-0.28507	-1.04861	-0.11653	0.415626551	1.044469394	-2	0.334809453	0.792528002	-0.184646315	-0.594269432	0.436986131	0.474707879	-0.275605643	-0.913102702	-1.21176049	-1.100410731	-1.266787169	-1.293891267	0.579884233	-0.924776444	0.452473459	-1.062361061	0.740535
TCGA.F2.A8YN.01A	-1.43186	-1.02513	-1.22582	0.887754	1.119101	0.319753	0.667263	0.005227	0.477932824	-0.857743974	-1.179811	0.737415172	-1.629663345	1.551148562	-0.274045868	0.172945076	0.408456664	1.336988883	1.338290318	-0.391337275	0.336000398	1.638248217	1.619451744	-0.426267015	-0.465476549	-0.075817254	0.418535006	-0.50146
TCGA.FB.A4P5.01A	1.72454	0.938067	1.464732	-0.54261	-0.97378	-0.96299	-1.3609	-2	-0.631436102	2	-0.927993	-0.522109171	1.992608363	-2	0.87563361	0.227150219	1.51920959	-0.837964466	-1.196915229	-0.3234665	-1.175542299	-1.823270688	-1.561425995	1.344780576	0.294521657	0.260294279	-2	-1.43925
TCGA.FB.A7DR.01A	-0.9201	-1.71982	-0.33527	-1.21652	-0.72764	0.766298	-0.30906	1.542272	0.62138351	-0.337042568	0.0032166	0.479928192	-0.570741108	-0.020312531	0.349569775	1.224567623	0.102478686	-1.065554755	-0.776646871	1.917020103	1.099837401	1.179123942	0.350848383	0.503563291	-0.969215834	1.719935372	-0.796530227	0.890174
TCGA.FB.AAPQ.01A	-0.81314	0.347773	-1.16295	1.355914	1.439334	1.566408	1.127353	0.514154	-0.001664007	-0.82653914	-0.0077562	-1.026919685	-0.970089696	-0.479252402	-1.357611454	-0.944162586	-0.928080985	-0.494224895	1.06929442	-0.501381139	2	1.642270482	0.133077509	-1.334737694	-1.037578882	-0.389406567	1.416133466	0.501148
TCGA.FB.AAQ1.01A	-0.77252	-0.25104	-1.00745	2	0.767194	-0.74216	0.759641	-0.5809	0.53198059	-0.856194409	-0.0033784	0.971079844	-1.358613709	1.367187519	0.79189311	-0.356890823	0.640402869	-0.236367669	1.739017714	0.230850277	0.117729395	-0.504223132	0.22638163	0.237772182	0.651837445	-1.188585504	-0.049544479	-0.71133
TCGA.FB.AAQ2.01A	0.23465	1.255018	-0.35	0.86895	0.390326	0.405219	0.186468	0.732636	0.830050911	-1.0505	0.147252	1.213045841	0.141108717	1.120922951	-1.6439	-0.5766	-1.4969	0.791462039	0.158494718	-0.3452	0.865962262	0.032549481	0.064328844	-0.4428	0.104269426	-1.5239	-0.2746	0.674551

			121							39002					57419	03548	34587			0471				57795		6257	89243	
TCGA.FB.AAQ3.01A	-1.147745	0.259905	0.760463	0.421373	0.89016	0.70231	0.673227	-0.60102	-0.939682667	-1.217127384	-0.01199	2	-1.175773615	0.725652312	0.871593078	-0.061801367	-0.656073608	-0.333436767	0.576933906	-0.76343879	1.618955685	-0.225824821	0.114516777	-0.676953991	-1.254581875	-0.475587617	0.575531535	0.5998
TCGA.H6.8124.01A	-0.928859	0.039215	-1.49838	0.985673	1.066945	0.54713	1.351805	-0.29764	0.513285145	-0.545303436	0.0104401	0.897735434	-1.402743073	1.18412411	-1.197160703	-0.572682807	-2	1.477221161	-0.314984016	1.678259892	1.171707601	-0.077399115	1.460372968	-0.605446006	-1.371862913	0.069099197	0.593482945	1.350438
TCGA.HV.A5A3.01A	-0.95929	-1.01075	-1.0417	1.000501	1.510869	1.209491	1.809091	0.784241	-0.50103058	0.110219572	-0.005216	-0.169046101	-0.540822046	0.956371588	-1.211396114	-1.072001408	-1.196710318	-0.126349772	0.267856195	1.634990722	0.176820448	0.484431755	1.647883348	-0.555190543	-0.688983574	-1.436501336	1.433966161	0.215485
TCGA.HV.A7OL.01A	1.465036	0.173432	1.584033	-0.18016	-0.28448	1.215794	-0.08446	0.356339	-1.727156292	0.265034398	-0.681755	0.850464065	1.047284175	-0.3252676	0.162180077	-0.493259169	1.050340607	-1.204139952	0.787650859	-1.358808259	-2	0.356342583	-1.587997538	-0.193540485	-0.354354435	-0.847700266	-0.932105966	-0.93525
TCGA.HV.AA8X.01A	-0.42566	0.367558	0.043667	0.442837	1.392988	0.510862	0.150663	-0.25115	-1.631677097	0.731450114	-0.476724	0.53144666	-0.717653263	0.7752755	-1.77126862	-1.46350342	-1.513059103	0.564028806	0.547663212	0.176172802	0.542155015	0.524364032	0.670066507	-1.217918901	-0.153630321	0.504651718	0.576250846	1.602543
TCGA.HZ.7289.01A	-0.63452	-0.36221	-0.13307	0.407245	0.964984	1.821837	0.44936	1.084742	0.892584543	-1.435833989	0.462585	0.565389541	-0.918975132	-0.47463464	-0.097140374	-0.79834072	-1.146490545	1.579698199	1.702477527	-1.029414898	0.648882059	1.392209494	0.826170976	-1.875712266	-1.000624839	-2	1.609396991	0.985653
TCGA.HZ.7918.01A	0.821077	1.006085	1.601821	-0.38665	-1.3742	-0.70342	-0.46352	0.561023	0.462862698	0.940354655	-1.524599	-1.664498093	0.800980623	-0.477336435	-0.551534519	0.11711882	1.07271806	-0.495161318	-1.068846958	0.052197556	0.116786399	-1.717303009	-1.360349704	1.008185474	1.233557598	1.073383592	-1.122916325	-1.3502
TCGA.HZ.7919.01A	-0.76	1.431186	1.034151	-0.12	0.43231	-0.92	0.813768	-0.17	-0.4927	0.597159411	-0.179	-0.1553	-0.1623	0.28005748	-1.3190	0.57933338	-0.1851	0.906927407	-0.3778	0.589148524	0.596277617	-1.5688	-0.6088	-0.3036	0.551253233	-2	0.246352716	2

	683			364		962		834	70078		629	22058	27967		53401		84412		08941			94383	97693	75673				
TCGA.HZ.7922.01A	0.495152	0.279459	-0.90659	-0.63543	-1.37829	-0.96017	0.857246	0.705817	0.373024131	-0.117766653	-1.547912	-0.517304064	0.910368248	-0.655829559	-0.421856772	-0.077493487	-0.13613731	1.732663059	0.629600508	1.407649265	-1.79703446	-0.123655736	0.200310622	0.957701814	0.838083959	0.662963723	-0.634081248	0.848035
TCGA.HZ.7925.01A	-1.59703	0.190499	-2	-1.02245	-0.33438	-0.4602	-0.00997	1.277164	1.198785442	0.061504584	-0.056721	0.923982901	-0.693506926	-0.21597665	0.592399493	1.474968319	0.400297341	0.951108013	-1.518894701	1.325457077	0.746933522	1.371733795	0.474675111	0.89462627	0.186842247	-0.19112738	-0.925552037	0.990273
TCGA.HZ.7926.01A	1.010644	0.76444	0.611226	-0.9957	-1.0773	-0.30682	-0.13826	0.719627	-1.10646182	-0.725105491	0.1537308	-1.97446227	1.637965422	1.335995564	-1.614808803	-0.439709381	-0.96575055	-0.595915614	1.364202795	-0.961233521	0.487169485	0.812047875	0.088406828	-0.430355742	-1.883575447	1.158229634	0.343940732	0.484817
TCGA.HZ.8003.01A	1.446522	0.649227	0.96873	-0.61297	-1.52835	-1.3781	-2	-0.19509	0.018101406	0.400642665	0.3934088	-1.530409837	1.734283934	-0.39284287	0.658041093	1.091530053	0.86233431	0.085322036	-1.2706287	-1.320830539	-1.616196632	0.693891705	-1.347858741	1.059414933	1.231067971	-0.093901252	-0.850508012	-0.88411
TCGA.HZ.8005.01A	-1.44434	0.666469	-0.37819	0.718875	-0.39305	0.160959	0.843176	1.942959	0.49447731	0.781415984	-2	0.564369838	-0.281049843	-0.944384869	-0.753110489	-0.14559881	0.285872582	0.022520823	-1.667368421	1.408575368	0.639032738	-0.517996752	1.013188831	1.012645141	0.311832989	1.057715882	-0.463977203	-0.68005
TCGA.HZ.8315.01A	-0.24731	0.520295	0.658639	-0.12768	-1.32021	-2	-1.05462	0.2379	0.080485103	1.739677989	0.606982	-0.218061433	0.047050498	0.882502535	0.24051245	0.207891113	0.480142477	0.043698049	-0.93221466	1.756052575	-1.255168184	-0.704897909	-0.599560315	0.782078522	1.322245997	-0.105096815	-0.235998393	0.296197
TCGA.HZ.8317.01A	-0.79112	0.666583	-1.37323	-0.25737	0.078995	-0.98019	-0.65271	0.41575	1.471362481	-1.763249858	0.8205833	0.532924684	-1.420696629	0.228027507	-0.228872555	1.432393658	-1.490946957	1.242680051	-0.784389066	-0.638165157	2	2	0.531922053	-0.200112656	-1.981331934	0.459548866	0.448613689	2
TCGA.HZ.8636.01A	-0.97	0.422852	-1.15	-0.26	-1.30764	-0.90	0.656412	0.201332	0.534758984	0.76247009	-0.852	-0.4191	-0.0119	-0.6339	0.330012102	0.854245087	1.026481642	0.933306023	-0.9593	1.142597251	-0.9451	-0.1500	0.120225521	1.734290226	1.627551591	-0.2470	0.176205947	-0.01

	064		908	169		244					032	24755	08162	79408					05672		46824	10028				15368		928
TCGA.HZ.A4BH.01A	0.700762	2	1.108826	-0.78379	-0.50531	0.376768	-0.77759	-0.84191	-0.061657592	0.535292133	0.6834877	-2	1.17783352	-1.20224474	0.496876991	-1.131218896	0.054081715	-0.480790093	-2	0.13197087	-0.41324186	-1.34032701	-2	0.94984657	0.994289331	1.997793838	-1.402486458	0.361665
TCGA.HZ.A4BK.01A	0.328755	-1.23824	0.85606	0.527906	-0.02285	0.33771	0.276952	-1.02646	-1.40174826	0.302850288	-1.549302	-1.229327854	0.091952224	-0.379213573	1.658596161	0.152375426	0.850207692	-1.506492893	1.141467906	0.20532262	-2	-0.834727593	-0.630205946	0.384885245	0.110638559	1.381040632	0.726298631	-0.40413
TCGA.HZ.A77O.01A	-0.17788	-0.14786	-0.23808	2	0.866574	0.558241	-0.00884	0.428411	-0.872279493	-1.062362334	-0.535295	2	-1.329972977	0.255446418	0.075711294	-1.126330995	-0.656385129	0.14368907	-0.456108636	0.967711096	1.576381712	0.30182566	-0.296858139	-0.265407534	-1.847544394	-0.493745796	0.570843629	-0.6472
TCGA.HZ.A77Q.01A	0.960782	-0.3163	-0.00135	-0.30898	-1.06863	-0.84776	-0.79133	0.070729	-1.088825803	0.436639676	-0.858755	-0.197278103	1.387275363	-1.565217672	1.110882318	1.359958277	1.338578601	-1.113127765	-0.825633287	0.469633274	-1.184978901	0.497222697	-0.41785761	1.114112364	-0.397118503	0.962844481	-2	-0.39186
TCGA.IB.7644.01A	-1.36116	0.081023	0.396961	-0.37962	0.042166	-0.15896	-0.51201	1.659191	-0.342529762	0.311794334	-0.458135	0.867148053	-1.218832296	0.024827722	-1.193666375	0.487528472	-0.481812386	2	0.616505934	-0.40479099	1.01995733	0.267221146	-0.526232496	0.359230149	0.127795491	-0.810639064	0.903422456	1.880229
TCGA.IB.7645.01A	0.724778	0.27347	0.500077	-1.25831	-1.45799	-1.38938	-0.78745	0.151004	-0.059609223	0.290646887	0.2748243	-0.719596094	0.785917361	-0.897133503	0.841380055	2	-1.439243823	-0.146515553	-1.470737906	0.825450455	-0.950795385	0.009860563	-2	1.125465012	1.064128122	0.815658026	-1.550012102	-0.72773
TCGA.IB.7646.01A	-1.7372	1.632188	-0.92348	0.787878	0.023064	-0.75549	0.368095	1.054222	-0.730488335	0.401305007	-0.036187	-0.381086272	0.047285402	0.398621066	-0.524100106	0.846228667	0.62948638	-0.59222081	-0.502728082	0.276916756	1.167251626	0.302279712	-0.312940733	1.298769286	-0.292519867	-0.464836508	-1.081742615	0.619411
TCGA.IB.7649.01A	0.382472	-0.47	0.411184	0.288037	-0.70522	-1.81	0.83019	-0.75	0.076522067	-0.0464	-1.009	0.353734501	0.750386117	0.8024598	2	1.572709978	0.841467857	-1.0919	0.19870021	0.494113889	-0.7467	-1.8066	-1.5585	0.780479834	-0.0080	0.084308471	-0.6893	-0.77

		208				608		253		93142	581							29321			55583	17742	90462		86724		86039	839
TCGA.IB.7651.01A	0.423458	0.471366	0.557151	-0.04188	-1.34356	-0.64802	-0.00404	0.984936	0.581656071	1.333888898	-2	-0.109782117	0.915775738	-1.326264835	-0.822049089	-0.134391174	1.065991778	-1.000339928	-0.303179845	1.012574617	0.12393844	-1.781320112	-0.638587537	1.160895631	0.085705235	0.492646803	-0.695047677	-0.24016
TCGA.IB.7652.01A	-0.40742	-1.23651	-1.69925	0.504593	-1.48022	-0.35906	-0.9822	0.175167	-0.09862781	-0.437663863	-0.712216	0.004576234	0.447743348	1.04234388	1.555297843	1.476335569	1.126016564	-1.414745856	0.260036143	0.44713945	0.155371524	-0.455223997	-0.035850303	1.471437208	1.777196215	0.481039241	-0.555579896	-0.17273
TCGA.IB.7654.01A	0.421032	-1.75393	-1.1836	0.212419	0.761612	0.387335	-0.18961	0.862687	0.336049311	-0.463640576	-0.366875	-0.118872948	0.151384858	0.448473142	-0.551648436	-0.085023379	-1.44362955	1.993324156	0.056405565	2	1.019990501	-0.841620797	-0.050968257	-0.690801887	0.066994686	1.517094499	0.671667493	-1.08111
TCGA.IB.7885.01A	0.795246	0.28238	0.270346	0.366987	-2	-0.85646	-1.81105	0.740871	-1.553904606	-0.265819878	-2	1.034101835	0.874593054	0.304922487	0.435549638	-0.758823475	1.191957297	-1.151650524	-0.470346851	-0.271061994	-0.80054287	1.275772145	-0.727360407	1.382367448	-0.960190381	1.863046398	-0.707330574	-0.65012
TCGA.IB.7886.01A	-1.10646	1.083687	0.370894	-1.18523	-0.46412	0.122833	-0.19907	1.799001	-1.026649099	1.465327067	-0.57131	0.500075182	0.32060548	-1.72330743	-0.080385619	0.94717702	0.440108485	-0.037742789	-0.504030164	0.228776136	0.152697692	0.500283507	-1.269763554	1.026501457	0.810020458	-0.566280085	-0.598620477	0.98301
TCGA.IB.7887.01A	-0.68863	-1.09119	1.135316	0.074071	0.198883	0.302956	0.158641	1.26953	0.477333364	-0.095136317	-2	-0.011611623	-1.245389003	1.184382907	-1.815406339	-0.500913683	0.129257377	0.347031377	-1.325853275	1.820629158	0.277165831	1.433444337	-0.245573186	-0.178033339	-0.1445578	0.635963571	0.6042502	0.654545
TCGA.IB.7888.01A	1.611189	0.194375	0.737581	-1.52573	-1.72355	-0.92386	-0.45073	-0.66103	0.300233761	0.780157888	-0.172765	-1.990727883	1.900383603	-0.206601278	0.477192962	0.831739275	1.521193485	0.016524214	-0.934866601	-0.701823735	-2	0.369174398	-1.327427519	0.664894314	0.605299709	0.380660183	-1.384226465	-0.54823
TCGA.IB.7890.01A	-0.25	-1.3181	-2	0.656817	-0.12889	-1.16	1.34156	0.853964	1.40821086	0.256803872	-0.686	-0.3611	-0.2994	0.338492598	0.476688942	0.991942636	-0.0707	0.07260874	-1.6822	1.648225946	0.394860508	-0.7981	0.026064743	1.132004732	2	0.614566087	-1.4674	1.048674

	863					679					024	18486	66208				39812		73675			03022					21028	
TCGA.IB.7891.01A	1.073061	0.207309	0.24254	0.077568	-1.78082	-1.19325	-0.72571	-0.52372	-0.378153758	1.500307286	-1.3181	-0.961640869	1.027575159	-0.120185923	0.583612957	0.792674252	0.98886871	-0.855619779	-0.438324365	0.496144331	-0.679522387	-1.654272851	-1.518717727	1.310132611	1.509586271	0.948476171	-0.886647076	-0.94493
TCGA.IB.7893.01A	-1.17747	0.284696	-2	0.662433	-0.12098	-0.87098	0.69461	1.117558	1.294164396	-0.51266585	-0.151758	0.168660389	-0.774554828	0.891099343	0.33257178	0.662895082	0.029310489	1.194407413	-1.134278161	1.219174234	1.129005009	-1.135260272	0.016997848	0.801329548	2	0.089163668	-1.51303545	0.96847
TCGA.IB.8127.01A	-0.654336	0.176753	0.495225	0.350857	0.427927	-0.550337	0.274938	1.134829	0.900673667	0.623984099	-1.485428	0.389462607	-0.26604509	-0.599650784	0.629052541	0.969263029	0.497985779	0.674870088	-0.9109992	0.643421518	-0.641798305	-1.061765578	-1.245106062	0.872255243	0.490107122	0.564858983	-0.489410548	-1.04851
TCGA.IB.A5SO.01A	1.568754	0.669172	0.542625	-2	-0.796	-0.381134	-0.54338	0.70683	-0.711535378	1.64827715	0.5405958	-2	1.780920539	-1.736908693	0.678319814	0.598218346	0.921694742	-0.941688369	-1.660445131	0.8146145	-1.640358139	-2	-1.085545818	0.723801284	0.742723409	0.645064665	-1.485185304	0.01308
TCGA.IB.A5SS.01A	-0.92183	-0.64908	-0.90135	0.580139	-0.70146	0.545896	0.97312	1.597198	-0.532969784	-0.051368074	-1.298981	2	-0.967982518	-0.351239865	0.757779167	-0.549776878	0.693481605	0.283443258	-0.312333778	0.055475398	-0.266555893	-0.676908588	1.011623855	1.184140095	-0.720701367	1.020580623	-0.066532319	0.004412
TCGA.IB.A5ST.01A	1.881228	0.228055	1.092133	-1.77475	-1.17037	-0.82538	-1.13528	-0.65911	-0.276671132	0.585805806	-0.980168	-0.12376045	2	-2	1.112189956	1.011869839	1.293213871	-0.853168344	-0.854783264	-0.528137437	-2	0.670317914	-0.753077636	0.520673287	0.704295784	0.723239811	-1.81951678	-2
TCGA.IB.A6UG.01A	1.22101	0.665282	0.537512	0.771737	0.289265	0.124332	0.169339	-1.04924	-0.115353778	-0.230963948	0.8503674	0.685707222	0.755862125	-0.449285491	-0.473383136	-0.947473397	-1.240866001	0.742244485	0.451728213	-1.862037493	-0.028010891	0.53754971	0.295030043	-1.07366929	-1.899058081	-1.077273273	0.323158333	0.451942
TCGA.IB.A7LX.01A	-1.07	0.054104	-0.73	0.379507	1.61424	1.304285	1.289654	0.084565	0.573066577	0.472488122	0.1806957	0.740217536	-0.5464	-0.2234	-0.7749	-1.0019	-1.6645	-0.2067	-0.6570	1.265867476	0.860136044	0.896989644	0.95358591	-1.3943	-1.3054	0.671827674	0.461274257	0.707382

	362		522										45149	84698	27698	22648	03091	6227	34331					18035	18471			
TCGA.IB.A7M4.01A	0.769506	1.530083	2	-1.37228	1.865567	1.250026	0.785069	0.10213	-1.070966811	-0.565835856	-2	0.783907966	0.894525714	-0.524650775	-1.551800228	-1.904645026	-0.618286881	0.683525108	-0.185957703	-0.834635874	-1.049907324	0.662210778	-1.128634682	-0.548048937	-1.331503192	-0.5790645	-0.742846431	0.749927
TCGA.IB.AAUN.01A	0.782123	1.446823	0.076442	0.63298	0.756809	-0.43715	0.189918	-0.39703	-0.288968768	1.413300014	-1.230329	-0.200482271	0.135794534	-0.68132156	-1.608097627	-1.379308304	-1.613967074	0.447872161	-1.113579089	-0.038373145	0.808187697	0.109191577	1.028538595	-0.977501084	-0.522242981	-0.774427292	0.914439992	0.831627
TCGA.IB.AAUO.01A	-0.92485	0.669141	0.68824	1.793415	-0.30502	1.053572	0.941833	0.501938	-1.816304746	0.262462903	-0.632984	0.083083368	-0.655800266	0.051358089	0.320297321	-2	-0.0095959	0.549893507	-0.283152223	0.711302843	-0.08192587	0.484484208	-0.665994335	-0.729814566	-0.261606694	0.524083682	1.13551466	-0.31637
TCGA.IB.AAUP.01A	1.991039	1.440173	1.21739	-1.19044	-0.8958	-0.22967	-0.56541	-1.53216	0.585177063	1.48889915	-1.442284	-1.263619335	2	-2	-0.332248225	-0.029548651	0.920058387	0.623436861	-1.516807828	0.001422597	-0.624404188	-1.45781935	-1.22014141	0.529112432	0.403188173	-0.138209184	-1.871561254	-0.71268
TCGA.IB.AAUS.01A	0.08485	-0.45905	0.435196	0.334957	-1.14652	-0.74827	-0.51412	0.135481	-0.687530683	-0.016314896	-0.309872	0.148088178	0.381428911	-1.085866706	2	1.539190645	1.910983169	-1.517914804	-0.429042131	0.042125522	-0.420400229	-0.082189774	-0.378265907	1.577314317	0.623055426	0.126027709	-1.979408019	-1.92456
TCGA.IB.AAUU.01A	-1.71988	-1.1325	-1.43092	1.024689	1.005931	0.786662	0.957481	0.991028	1.179799659	-1.641386756	0.0492094	0.476923319	-1.34459609	0.597803634	0.293641013	-0.365279549	0.776616285	-0.29035096	1.358865204	-0.558018187	0.444715287	0.671305706	0.0413672	0.799308511	0.129289055	-0.970991449	1.351423696	-0.77738
TCGA.LB.A7SX.01A	0.311788	1.127601	0.418528	-0.42532	0.77976	0.565603	0.616666	0.008592	-0.825234519	-0.141369576	0.7225641	0.102711374	-0.514142175	0.485015978	-0.460730028	-2	0.061940773	1.276888366	-0.236784009	-0.015800456	-1.092373812	1.447400845	0.443010622	-1.133372266	-1.41144949	-0.851292025	0.285687232	0.75256
TCGA.M8.A5N4.01A	-1.34	-0.70	-1.92	1.965968	-0.96835	0.619632	0.754265	1.070583	-1.8472	-0.0300	-0.58924	-0.6044	-0.0577	0.344171861	0.796746862	-0.3322	1.336391354	-0.9026	-0.0915	0.860580769	0.525635873	1.519766985	0.411029483	0.739091289	0.476781278	-0.1574	0.608068059	-0.90

	024	984	168						59928	47897		26592	40573			24197		2677	11566							45213		889
TCGA.OE.A75W.01A	-1.09341	0.584874	1.687664	0.228699	0.920382	1.563424	0.632003	0.275449	-1.563014465	1.199337189	-0.785385	0.800670272	-0.700021741	-1.837645984	0.256018188	0.367653683	0.597621068	-0.017358347	0.13019558	-0.529376693	0.35243102	0.598996312	-0.052387892	-1.027697907	-1.083401374	-0.609557105	0.420015341	-1.10708
TCGA.PZ.A5RE.01A	-1.40372	-1.31298	-0.98678	0.778564	-0.34471	1.247019	0.369987	1.928077	-0.471798711	0.368131949	0.2363878	0.696077831	-0.142389284	0.975860967	1.867257682	0.466943369	0.856187936	-0.648419681	0.157599252	0.403450049	-0.992305407	0.469117849	0.368214476	0.789213512	0.418843975	-1.954500347	-0.049190105	-0.39483
TCGA.Q3.A5QY.01A	1.928979	0.863949	1.216266	-0.83114	-2	-0.38179	-0.54613	-2	-0.350044227	1.349478052	0.3578482	0.294248592	2	-2	0.559365165	-0.487246883	1.119227777	-0.155966668	0.005046094	-1.288281937	-0.516993662	-1.564956644	-1.2335433	0.3574879	0.198718224	0.253374404	-1.767833068	-0.77615
TCGA.Q3.AA2A.01A	-0.85836	0.044047	-0.35583	0.791163	0.848367	1.418186	1.258006	0.830799	-1.424269944	-0.4727847	0.7528723	2	-1.078530503	0.607067173	0.898080888	-0.612154809	-0.429848103	-1.293213034	1.10749292	0.219383791	0.226400382	-0.308387883	0.566154506	-1.013075823	-1.587751523	-1.96289019	1.58756371	-0.34701
TCGA.RB.A7B8.01A	0.092047	-0.84812	0.945647	0.386758	-1.27919	0.067997	0.5332	0.503717	-1.486245003	-0.965708077	-1.090523	0.101814268	0.373042213	-1.385477233	1.038129972	-0.177127826	0.826468242	-1.315901817	-0.90800433	0.092510297	-0.740226005	0.07414125	-1.092741159	1.247078053	0.652492599	1.631533873	0.569420705	-0.21803
TCGA.S4.A8RP.01A	-0.54669	-0.14435	-0.59591	0.246698	0.270723	-0.57032	0.173269	-0.63658	-1.257607434	2	-0.380362	0.972216372	-0.91215501	0.90741607	0.989536354	-0.598315008	0.493921185	-0.387311804	-0.264257732	0.039613248	0.061971863	-1.144141194	0.384387221	0.677345643	0.231669355	-1.199036804	1.140516897	0.358769
TCGA.US.A774.01A	1.537674	-1.62189	0.728659	-0.0256	-0.92919	-1.17131	-1.61633	0.677867	-1.635757828	0.175254439	-1.098854	0.408302884	1.129534834	-0.299181443	0.535881531	-0.231845827	0.588169595	-0.672140043	-0.847847463	0.263096998	-0.617522133	-0.694927792	-1.298886144	1.454510706	1.053920768	1.467609567	-0.217409417	-1.76854
TCGA.XN.A8T3.01A	-1.11	1.091539	0.597394	0.423707	0.570258	-0.33	0.035947	-1.98	0.353980952	1.042358383	0.0563265	-0.4480	-1.3291	1.044275568	-0.6147	-0.2197	0.022735836	0.526016715	0.089987023	1.740440168	0.224023292	-1.3089	1.020540107	-0.4566	0.152574169	-0.1237	0.094111304	0.748684

	844					099		584				55716	53857		76394	77411						47661		60381		38045		
TCGA.YB.A89D.01A	-0.9136	-0.65594	-0.53964	-0.58634	-0.30689	0.304001	-0.08414	1.71024	0.393840566	1.643255518	-0.0219	-0.331677381	-0.780297642	-1.83220047	0.62935732	1.693076823	1.064318116	-0.655641141	-0.590971204	0.969808488	-0.320373706	-1.057471212	-0.284677995	1.816330565	1.399089754	0.207377344	-0.325848954	-0.52511
TCGA.YH.A8SY.01A	-0.84951	0.757894	-1.99279	1.482026	-0.3779	1.308486	0.429833	1.603617	-0.762357575	-1.390173255	0.8411035	0.263761099	-1.105066178	0.109019309	-0.62044912	0.14744932	-0.269482069	0.583525066	-0.564026679	0.531593434	1.201038623	0.652454791	0.780253057	0.524674006	0.150177049	0.050668548	-0.315993204	0.557243
TCGA.YY.A8LH.01A	-0.10541	0.174787	0.140464	0.524202	1.777906	1.849401	1.190983	0.765174	-1.106846466	-0.498303498	0.4321663	-0.221819788	-0.313551428	-0.540086764	-0.583772789	-0.495674546	-0.886415012	0.556118856	0.799567851	-0.880869282	-0.036230092	0.583154649	-0.341628653	-1.933470757	-0.490085123	-1.160124974	1.286202594	0.616991
TCGA.2J.AAB4.01A	-0.89512	1.113786	-0.04747	0.090593	0.518257	-1.51626	-1.48621	0.162421	0.288638954	0.620996162	-0.215407	-1.19939108	-0.971079233	1.382355577	-0.92834767	-0.636258933	0.24952742	-0.544679958	-0.30974243	-0.788383742	2	0.581969508	-0.086165785	-0.36674325	1.275062683	0.100556766	1.586856663	0.388734
TCGA.2J.AAB6.01A	-1.61508	0.310301	-1.63128	1.540548	1.247324	0.44128	0.229255	0.604128	0.988742486	-0.913697781	-0.601791	1.293950539	-0.533713626	1.244629583	-0.556883049	0.215803073	-0.084356888	0.091299119	-0.059080051	1.614550205	0.622503541	0.08055138	0.860823937	0.368781988	-0.274651757	-1.194357514	-0.460026329	-0.03606
TCGA.2J.AAB9.01A	0.366832	-1.65515	0.576029	0.569818	-0.002816	-1.32839	0.145082	-1.55429	0.073165406	0.049822428	-0.843862	0.021858116	-1.491669755	1.209922661	0.951399082	0.268688564	-0.066968486	-1.295583602	0.872858801	1.633037195	0.841534234	-1.014767997	-1.804296303	0.06515506	0.7819005	2	-0.176793193	-0.02478
TCGA.2J.AABE.01A	-0.92663	-1.46114	-2	0.553328	-0.24192	0.373766	0.551428	0.494876	-1.724568796	-1.456615215	0.1743164	-0.363359825	-0.09235377	0.265162067	1.973624955	0.596572907	0.923604166	0.182448058	0.049033819	0.968899074	0.226688085	0.299460444	1.386099487	0.588305845	-0.101057574	0.159638468	0.628487812	-0.09659
TCGA.2J.AABH.01A	-0.84	-0.72	-0.89	1.557782	0.82783	0.538474	1.418734	1.112035	-1.3760	0.507501639	-0.47332	-1.2407	-0.9813	1.337427075	-0.7053	0.140934921	-0.7787	0.953219284	0.350210811	1.80707028	0.800641752	1.178620707	-0.2153	-0.9529	0.883650468	-0.8116	0.917714651	-0.24

	971	076	404						57816			41198	75277		41856		00189						96828	62515		21475		927
TCGA.2J.AABI.01A	1.188462	1.110642	0.800126	0.00131	0.178091	0.670373	-0.26612	-1.49348	0.409336362	0.668498353	-0.985263	-0.151620591	0.995341515	-0.872144076	-0.381569147	-1.158020001	0.641310933	-0.868681395	-0.580025702	-0.755692261	-0.527434545	-0.249482488	0.344725928	-0.303404601	-2	0.205532868	-0.496220373	-0.28899
TCGA.2J.AABK.01A	0.230347	0.438772	0.502137	-0.33243	1.856023	0.39852	-0.16203	0.403067	0.155204939	-1.189049495	1.0407741	-0.034825511	-0.788152669	1.615999754	-0.472029796	-0.784437345	-1.104678028	0.21703632	0.436862074	-1.705402456	0.130013753	1.154712001	-0.691054708	-2	0.069261332	-0.921420399	1.334855027	0.601301
TCGA.2J.AABT.01A	-0.45044	-0.60953	-0.85162	-0.35391	-2	-1.04328	-0.35067	-0.189	1.746401572	0.746379164	0.2127301	0.3636102	0.547112387	-0.467938663	1.762653702	0.261857386	1.408198413	-1.139329326	-1.033151675	0.920255161	-0.467472712	0.3286939	0.090857716	0.834798949	0.48312436	0.694066617	-1.131655085	0.588366
TCGA.2J.AABV.01A	0.59777	-0.38022	0.214688	0.514725	0.198089	0.498403	-1.91657	-1.21407	0.370097673	-1.141249946	2	0.200997001	0.087448539	-0.468492714	0.770594613	1.158691703	-1.013936208	0.625586822	-0.563319851	-2	2	1.73832966	-1.170440661	-0.977573817	-0.509140704	-0.29114706	1.1348403	-0.64127
TCGA.2L.AAQM.01A	-0.27025	-1.98993	0.814118	-0.52855	0.517906	1.706683	0.391966	-0.85329	1.050115671	-0.396563907	0.4506209	1.07527027	-0.526002591	-1.066313682	-1.454205934	-0.665592605	-1.197065859	0.337099952	2	-0.250862661	1.172161117	0.941259603	2	-0.724369124	0.046133546	-1.219939185	0.615170898	-0.18574
TCGA.3A.A9I5.01A	0.580404	0.057271	-0.3621	-0.02341	1.139533	0.311625	0.950301	0.486984	0.593635157	-1.953989342	0.3148855	1.332902594	0.238318096	-0.259120114	0.05084055	-1.332772534	0.952094961	-0.52511042	2	-0.623800931	0.597101989	0.843942341	-0.033012671	-1.419338097	0.612313371	-0.804524572	-0.21619598	-0.41701
TCGA.3A.A9I9.01A	-0.563	-1.57599	-0.27911	1.69504	-0.00547	0.095848	-1.77834	-1.56709	-0.943023346	-0.685586003	0.4342087	0.806382773	-0.507173404	0.922830707	0.987027103	1.301987763	0.896401071	-1.001584175	0.205621657	-0.415359614	0.521077792	1.826321976	0.812827438	0.080161766	1.114896784	-1.483638009	1.383192955	-1.75292
TCGA.3A.A9IC.01A	-1.20	-0.23	-1.31	-0.29	0.410896	0.21822	0.303599	1.689361	-0.8097	-0.8951	1.1908413	0.915586912	-1.5954	0.927224524	0.969750025	0.531082499	-0.2296	-0.1342	-0.7319	1.048388	1.087205461	1.355749245	0.138755201	-0.0411	0.35260259	-0.3138	-0.4658	1.352256

	893	304	162	634					45271	27584			47184				01987	43689	78325					69315		8344	00846	
TCGA.3A.A9IJ.01A	-0.43696	-1.60877	-0.06515	-0.06499	1.130182	0.396635	0.503187	-0.36321	2	-1.717433514	2	0.631695432	-0.327675787	1.406522886	-0.184899988	-0.168091494	-1.322444286	0.512620578	1.433282783	-0.116683735	-0.060004528	0.347435613	1.270270752	-0.57815297	0.543653175	-1.237076998	0.642979536	-0.6262
TCGA.3A.A9IL.01A	-0.12621	-2	-0.59861	-0.22361	0.854626	0.528345	-1.09788	0.286064	1.103644825	-0.289242697	2	-0.433308086	-0.5834333	0.745638713	-0.933699275	-0.076816186	-1.035685693	1.31540252	2	-1.106956543	1.623900043	0.127118553	2	-0.087759169	0.733706408	-1.761791079	0.811217429	-0.071222
TCGA.3A.A9IN.01A	-0.16517	-1.66541	-0.06506	-1.11594	0.54863	0.610517	-1.35002	-0.57457	0.758886152	0.256042243	2	0.102510955	-0.506982365	0.60498356	-0.814095622	1.061660868	-1.567107616	1.7639514	0.065241911	-0.968498411	0.388387863	0.201596728	1.659133792	-0.007333455	1.274985236	0.153308322	0.608371648	-0.076648
TCGA.3A.A9IO.01A	-0.08792	-1.54847	0.12547	-0.25947	0.919347	-0.46003	-0.30499	0.549523	2	-2	2	1.226532237	-0.397675117	1.385900329	-0.975119662	-0.685719915	-1.513288921	1.371888484	0.850059516	-0.423293259	0.430505264	0.537482025	1.502149416	-0.401965152	0.804557335	-1.684401676	0.594552818	-0.06897
TCGA.3A.A9IR.01A	-0.3395	-1.66336	-0.06121	0.850394	1.156859	1.22777	-0.22944	-0.39547	1.485667353	-1.876064223	1.5921531	0.931493001	-0.658809283	0.52828057	-1.5777006	-0.101357342	-1.413902955	1.11213765	1.777683981	0.101021924	0.515412474	0.844499312	2	-0.786342321	-0.275982098	-1.743014653	1.465624378	-1.24987
TCGA.3A.A9IS.01A	-0.13039	-0.73957	-0.27237	-0.2828	0.539987	-0.09087	0.434	-0.16013	2	-1.43706401	1.8335884	0.236136204	-0.625776345	1.057744123	-1.252488399	-0.776356834	-1.547830523	1.486310651	2	0.149780781	0.461109258	0.374931049	1.640783146	-0.83460331	0.59575581	-1.939541919	0.915411679	0.522001
TCGA.3A.A9IV.01A	-0.36415	-0.43761	-0.14228	-0.13354	0.552567	0.253128	-1.02053	0.732607	2	-1.233623433	1.9967114	-0.168213517	-0.662039121	0.288038323	-1.752730648	-0.968486815	-1.259561345	2	1.972503491	-0.224972535	0.462556443	0.746729284	1.293811111	-0.274698566	0.166383497	-1.500865974	0.898680855	0.494448
TCGA.3A.A9J0.01A	0.153258	0.416025	-0.99	1.086801	0.944359	0.656829	1.276494	0.813923	-0.5188	-1.9914	0.0717994	1.165049803	-0.0783	-0.5187	-0.0723	0.377326659	-0.3882	0.690660203	-0.7167	0.892602342	-0.0282	-0.6553	-0.1646	-0.2513	0.428764698	-0.0696	-0.2129	-0.33

			558						90976	61626			70506	33782	26772		04379		86482		11606	94661	56869	84758		72075	48646	442
TCGA.3E.AAAY.01A	0.337686	0.038773	1.065939	-0.964559	-0.60114	-0.09617	-1.52753	-0.13221	-0.042378102	1.735850301	0.9282359	-0.851071322	0.486352685	-0.538036773	1.410857842	0.898912059	1.249360483	-1.859245567	-0.603924859	0.28543324	-2	0.604419964	-1.759201363	0.922720253	0.408122166	0.617280421	-0.486813565	-1.09968
TCGA.3E.AAAZ.01A	-0.270996	-0.290662	-0.352998	-0.616612	0.479817	0.090749	1.624214	0.77202	0.990078945	-1.20712394	1.0392396	1.511272605	-0.698351554	0.123888361	-0.358198743	0.458887648	-1.628454657	0.622626362	-1.273200831	-0.387178008	1.241733061	0.321872872	1.108396825	-1.22260589	-1.287844724	-0.170054475	0.151797079	2
TCGA.F2.6880.01A	0.469562	-0.9117	-0.83855	0.41357	0.482113	0.246145	-2	-0.84454	1.323016158	-0.885542969	2	-0.021102057	0.405842677	0.737561535	0.107947243	0.822985264	-1.764478753	0.857463845	-0.200960094	-1.730339881	1.20195419	2	0.371721205	-1.33501397	0.585933267	-0.061543541	1.311355818	-0.19866
TCGA.F2.A44G.01A	-1.79466	0.778606	-1.56623	0.836099	-0.15956	0.733913	-0.1428	0.873087	0.17464651	-0.622550628	0.2312744	1.114017172	-1.687280351	0.213215108	0.033804362	-0.483539946	0.389992182	0.831973289	-0.158871471	0.178636032	0.634252718	1.649689169	0.904864284	0.214579482	-1.309294893	0.434289117	0.314550734	0.621022
TCGA.F2.A44H.01A	-0.78161	-1.0661	-2	1.23303	-0.06233	0.56153	-1.77891	-1.14077	1.36361224	0.315677272	-1.14842	0.756787086	-0.313399512	-0.041476379	1.487936803	1.449852102	1.026715047	-0.838877724	-0.544124502	-0.378079105	1.07173667	-0.060332061	0.167993889	1.312687483	0.744580394	0.781783317	-0.089527392	-0.96745
TCGA.FB.A4P6.01A	0.302616	-0.30191	0.311129	0.125587	-0.13363	-1.05666	0.150409	-0.89935	0.885704044	1.18427425	0.1613455	-1.62467502	0.133602163	0.599830855	0.505884849	0.213182649	0.541814426	-0.574274914	0.275692992	-0.324098381	-0.457248127	-1.258590775	0.203904355	0.305188935	-0.323954682	0.680277976	0.433371896	-0.606661
TCGA.FB.A545.01A	-0.73842	0.267212	-0.71076	0.56027	0.852623	1.09224	1.039739	0.604262	-1.504329754	-0.272716931	0.7010453	-0.146303537	-0.540761575	0.693477637	-1.183574125	-1.281215018	-1.124512639	1.325758824	-0.032405614	2	1.118868103	0.101599826	0.712831212	-1.282563236	-0.698726296	-0.684414912	0.394539977	1.550286
TCGA.FB.A5VM.01A	0.688241	1.449608	1.090327	-1.05	0.316799	0.807577	0.686382	-0.80	-1.0038	-0.4902	-0.699	1.669108809	-0.4063	-0.2016	1.619818776	-0.0719	0.77094241	-0.1450	-0.1474	1.5845	-2	-0.3965	-0.1576	-0.1083805	-0.6848	0.354827797	-1.3406	-0.22

				064				797	32762	29053	794		11867	1239		7788		27195	61119	94191		52457	33462		68209		52193	373
TCGA.FB.A78T.01A	-1.33907	-1.00808	-0.7592	1.604793	0.57716	1.218147	0.736416	0.901323	-0.512896165	0.58431485	0.5971272	0.043651552	-0.616210663	0.526249994	0.720887761	-0.453252105	1.249702434	-1.954048997	0.825365977	-1.20687566	0.265774789	-1.182385502	-0.529878503	-0.287436242	-0.196810272	0.383291686	1.380031124	-0.513332
TCGA.FB.APP.01A	-0.57806	0.406357	0.5621	0.004345	0.850506	1.410842	1.345893	1.115294	0.792984704	-1.398650818	0.6055653	0.877751349	-0.852638644	0.110092426	-0.592856264	-2	-1.102947211	0.444712591	2	-0.48262384	-0.316643606	0.887521802	1.548239192	-2	-1.307322494	-2	1.516581344	1.654405
TCGA.FB.APS.01A	1.272647	0.110055	0.727409	-1.35425	-1.35717	-0.00296	0.396185	-0.60992	-1.070951271	1.253201891	-0.499509	-0.100279989	1.348584354	-1.834698019	1.422002962	-0.61251491	0.750872718	-0.848341473	-1.333571209	1.242525462	-0.374428828	-1.888415648	0.057345304	1.132652769	0.030381615	1.021336665	-1.646422914	-1.51946
TCGA.FB.APU.01A	-0.2619	0.737741	-0.73126	1.100489	1.008375	0.775057	0.041121	1.020801	0.074945342	-0.720644469	1.3298954	-0.259861055	-0.555409497	1.079665461	-0.82568121	-1.070005807	-1.449620121	0.797533384	1.038052188	-0.908061176	1.100904436	0.25426861	0.5335513	-2	-0.618685163	-0.421694238	0.665912076	1.104201
TCGA.FB.APY.01A	1.383981	-0.04303	0.837876	0.563903	-0.79102	0.515045	-0.16662	-1.57363	-0.965895678	1.245135498	0.654544	-0.132665542	1.130221453	-0.641155532	1.137455742	0.332995717	0.874447035	-1.887866981	0.861716947	-1.05254112	-0.893097263	-0.46964889	-1.650942914	0.20961935	-0.656199212	-0.77975284	0.863939476	-2
TCGA.FB.APZ.01A	-0.62454	1.648247	0.571295	-0.32391	-0.16637	0.804798	1.039306	0.141434	-1.543460507	-1.143711975	0.0019353	0.583385122	-0.660780161	-0.046808053	1.068195213	0.908829006	-0.021187474	-1.291508352	-0.335925337	0.210603772	-0.076924867	-0.615323817	0.9216684	-0.063213908	-0.804453892	0.336009394	-1.104171003	0.752126
TCGA.FB.AQ0.01A	-0.97415	-0.54354	-0.62682	0.59033	1.652227	1.603707	1.218237	1.307306	-0.486014621	-0.709188292	0.730028	0.065890766	-0.708706385	-0.418596277	1.329748592	-1.023010961	-1.094979704	1.969184893	1.336972832	-0.339506254	1.692885169	0.473017729	1.163126038	-1.230900725	-0.104562166	-1.103032226	0.819137954	0.058559
TCGA.FB.AQ6.01A	-0.29	-0.05	-0.27	0.722085	1.828925	1.179827	1.342062	1.177976	0.68505338	-1.0676	-0.056	0.492276112	-0.7219	-0.0166	-1.1714	-1.9001	-0.8343	1.261089795	0.564535551	-1.0517	0.474160613	1.394739589	0.91407913	-1.7711	-1.1852	-0.6293	0.758162159	0.174752

	105	996	743							07888	007		26332	38365	09673	65003	00325			97252				71931	06655	7049		
TCGA.H6.A45N.01A	1.212309	0.477205	0.739377	-0.18687	-0.74317	-0.61251	-1.8138	-1.44446	-0.765349722	2	0.0990325	-2	1.013014446	-0.394932172	0.998298809	0.122840321	0.875903086	-0.298132661	-0.801064215	-0.386572189	-1.021159271	-1.473209902	-0.441861687	1.401045243	1.2243873853	-0.287479073	-0.656656398	-1.1442
TCGA.H8.A6C1.01A	0.764815	0.822403	-0.71446	-0.11543	0.140525	0.001422	-0.3595	-0.85604	1.297443807	0.463794594	-0.572918	-0.72668496	0.979006434	-0.174931313	-0.164298192	-1.150764712	0.120738797	0.182927485	-0.052477097	-1.110274796	-0.413135918	1.434659398	-0.519198085	-0.259401613	-0.538524815	-0.117269592	1.445532102	0.505941
TCGA.HV.A5A4.01A	-0.3892	-0.05195	-0.25465	0.454279	1.036126	0.398651	-0.88682	0.396529	-1.859910498	0.092053185	0.6089946	-0.622538169	-0.598277917	-1.147767916	-0.470660696	-1.618134841	-0.148729894	0.713530321	-0.443921303	0.803122768	-0.033433069	-0.911274918	-0.331944313	-0.265027533	1.154170666	-0.342929514	1.474011634	1.621036
TCGA.HV.A5A5.01A	-0.15801	-1.0399	0.051262	1.051282	0.382425	0.306098	-0.35572	-0.09325	-1.66782985	0.483215797	-0.188632	-0.663187439	-0.464109024	1.638545998	0.157458401	-1.057005163	0.760066798	-2	0.216474309	-0.315401384	-0.952409273	0.747065312	-0.797565594	0.785807056	2	0.292497113	0.500708701	-0.34675
TCGA.HV.A5A6.01A	-1.67246	0.163452	-0.78051	0.936167	1.059544	0.315509	1.8682	-1.07412	1.195203859	-0.102689518	-0.007678	0.764736484	-1.316848852	1.244060938	-0.51387859	-1.043530639	-1.400351292	0.626496	0.791761885	0.434105367	0.72546331	-0.523351675	1.384171449	-0.292386325	-0.961432607	-0.364541871	0.514029613	1.521537
TCGA.HV.A7OP.01A	-0.13931	0.018424	0.209965	1.164656	0.545185	1.30974	0.227143	-0.25413	-0.508775641	-0.901383604	0.5826132	0.333518229	-0.535666847	-0.289327073	-0.194095342	-0.51715247	-0.557986625	0.744490899	1.539641038	-0.259268991	0.385657435	1.234403088	-0.145005742	-1.521473105	0.260430452	0.02943541	0.44446391	-0.48066
TCGA.HV.A8V.01A	-0.08523	-2	-1.84013	1.043719	-0.19845	0.177059	0.062805	0.459624	-1.153044912	-0.4168727	-0.310468	1.30185501	-1.274330401	1.110079464	1.527971594	0.070239132	0.287875889	-0.951497485	-0.046763719	2	0.340904199	-0.616518392	0.5412575	1.015035571	1.435077299	0.182692797	0.470841218	-0.72662
TCGA.HZ.7920.01A	1.049412	0.094186	0.474587	-1.42	-1.14953	-2	-0.09	-0.8342	1.335257195	-0.0935	1.5187476	-1.4186	0.987519999	0.976066079	0.489267427	1.04391258	0.752552702	0.045343957	-0.2658	-1.7036	-0.8343	-0.6943	0.161021666	1.205159271	0.517043255	0.025331575	-1.3873	-0.41

				749			616			96759		35496							87909	40338	70199	85124					36395	288
TCGA.HZ.7923.01A	0.826167	0.346216	0.676458	-0.67458	-1.27869	-1.72252	-1.21392	-0.95426	0.830465574	1.254211078	0.8262028	-1.810611636	1.109193092	0.155126123	1.585376608	1.490377527	1.391523094	-0.234178622	-1.190020473	-0.597649729	-1.982586559	-0.65073989	-1.126633966	1.423995104	0.553918318	0.440038803	-1.968420937	-0.8077
TCGA.HZ.7924.01A	1.161522	0.923776	-0.15771	-1.26055	-0.10883	-0.72473	-0.382	0.554218	1.11518406	-1.167702145	-0.34175	0.209342585	0.725720114	1.055649256	-1.087649049	0.062022674	-1.078312248	0.282444713	0.986580067	-1.277450843	-0.964443917	1.045878612	0.691328703	-0.474651654	-0.443016075	-0.881040014	0.054999248	1.3631
TCGA.HZ.8001.01A	1.291507	-0.78721	1.139002	-1.99169	-0.62944	-0.84056	-0.31476	0.812109	-0.55423805	-0.15781909	-0.29065	0.656147786	0.719851599	-1.06770737	1.305662964	2	1.356384044	-1.565683813	-0.69666642	0.214686556	-1.422153342	0.243141743	-0.429663586	0.840235236	0.054108117	-0.320122717	-1.976945285	-1.07822
TCGA.HZ.8002.01A	0.194805	-0.07635	0.465342	-1.7504	-0.8011	-2	-0.31471	-0.70613	1.262975794	0.632484775	0.2016494	-0.756219634	0.072770509	0.43872828	1.155547956	1.924641375	0.828368705	-0.055777455	-1.155510437	0.011045546	-0.044940327	-0.381404141	-0.832630882	1.051849832	0.463877443	0.990465574	-0.975474472	-0.29944
TCGA.HZ.8519.01A	-0.04996	-0.57396	0.873681	-1.28439	-0.31667	-1.61059	-0.67266	-1.08407	1.687599984	1.287778164	1.504501	-1.785747807	0.23938704	0.87355242	-0.221240926	1.194641598	0.226506587	1.156838922	-0.090775669	-0.396495885	-1.29307994	0.214884241	-0.601723951	0.765311245	1.596065349	1.422362773	-1.307003174	-0.19627
TCGA.HZ.8637.01A	1.3631	1.702837	1.283176	0.074577	-1.3056	-1.32447	-1.69607	-0.84262	0.557506005	1.472161767	-1.125171	-0.497809943	1.727602757	-0.848511616	0.689157674	-0.599001641	1.524426671	-1.224538223	-1.065647035	-1.08375902	-1.53607599	-0.112590371	-2	-1.288405514	-1.18469104	-0.498785163	-1.533487369	-1.58725
TCGA.HZ.8638.01A	-0.15757	1.565866	1.17457	-1.14945	-0.43364	-0.65257	-0.47923	0.024957	1.357883731	0.126493977	-0.862684	-0.822164592	-0.704557828	0.912085999	-0.949093173	0.012311136	-0.810240125	-0.0073111	-0.217292476	-0.829483368	0.161948135	2	0.568956678	-1.270731758	-1.682195172	-0.057683991	0.522273752	2
TCGA.HZ.A49G.01A	-0.49	-1.02	0.038343	0.816338	-0.5001	-0.16	-1.86	0.10976	-0.5155	1.072905743	-0.309	-1.3205	0.204699971	0.325568897	1.684539875	0.649251874	1.703717138	-1.3613	0.108213375	-0.5564	0.166349627	-1.5029	-0.2684	1.550641349	1.749916551	0.637695286	-0.2570	-1.77

	609	578				283			25825		796	26587						6748		03571		06646	88576				51019	651
TCGA.HZ.A49H.01A	1.021314	0.749174	1.192671	-1.49097	-0.66815	-0.73379	-0.85361	-1.17288	0.425515402	0.914804348	1.0549268	-0.954730691	0.139007513	-0.451181832	0.27109289	1.092756815	1.092814874	-1.572731764	-1.41774897	-1.128451775	-1.226306396	0.999669772	-1.544928318	0.251734355	-0.917417917	0.936198087	-0.235660374	0.169409
TCGA.HZ.A49I.01A	0.189744	-1.10439	-0.06286	0.260699	-0.85259	0.960552	0.248779	-1.39799	-0.445084831	1.185158968	0.6112019	-1.735023101	0.378372466	0.800083771	2	-0.796786262	1.27514078	-2	0.858030125	-0.344829449	-1.484904312	-0.835514695	-0.49258745	0.545996509	1.711822679	0.873428336	0.120552122	-1.75954
TCGA.HZ.A77P.01A	-0.63295	-2	0.209927	-0.54112	-1.08744	-1.12578	0.76058	-0.30825	2	0.585208818	-0.167421	1.032970743	-0.828567552	0.704551653	1.229698178	1.483650841	0.749464752	-0.21674829	-1.1308196	0.419792745	-0.582212523	0.056924348	0.68929985	0.391278624	-0.055407573	-0.386747957	-0.276494066	1.140582
TCGA.HZ.A8P1.01A	-0.75338	0.490029	0.134753	-0.17101	0.944708	1.511278	0.663503	1.073407	1.070376801	-1.446874314	0.8282256	0.839267062	-1.015014246	-0.226851598	-0.432029934	-2	-0.894847437	1.560739971	0.896869943	-1.583741789	0.816058287	1.278654561	0.937485975	-2	-0.903459002	-0.481245705	0.855111648	1.488892
TCGA.HZ.A9TJ.01A	-0.39005	-0.93326	-1.01209	0.879549	1.989828	1.276593	1.359455	1.425855	-0.437683024	-0.958771257	1.2304125	-0.759960964	-0.607420174	1.606254064	-0.479230525	0.003835679	-1.6915008	-0.777118558	0.329829223	0.221941102	2	0.03567817	-0.639219334	-1.62930956	0.299367469	-0.784283221	1.073820714	-0.00279
TCGA.IB.7889.01A	1.460034	-0.31269	1.61095	-0.46274	-1.21229	-0.46003	-0.27146	-1.31929	-1.501293657	0.979277104	-1.773386	0.790911738	1.560631698	-0.788849043	1.030120341	0.740708116	1.240712905	-1.403769119	-0.743561945	-0.990340083	-0.839861369	-1.708911567	-1.442115259	0.495227912	0.117060696	0.078565861	-0.851153656	-1.1108
TCGA.IB.7897.01A	0.487911	0.359333	0.85213	-1.89781	-1.29572	-2	-0.88307	-0.67041	1.235351102	1.069407268	1.2145177	-1.406902784	0.66730141	0.332792792	0.155009972	1.37230348	0.777946578	0.582013743	-1.347107228	-0.093752203	-1.287717615	0.005726346	-0.945657085	1.053749182	1.192496218	0.657780913	-1.241019011	0.230934
TCGA.IB.8126.01A	1.299976	0.918571	1.37119	-1.09	-0.5623	-2	-2	-1.35	0.079715039	0.20893528	1.597877	-2	1.268040508	0.349295361	-0.0384	1.23035934	-0.0560	0.59551795	-1.4175	-2	-0.6250	0.79800314	-1.3475	0.045318775	0.655723359	1.510938607	-0.2654	-0.10

				706				233							42117		33161		19095		57114		08828				69976	712
TCGA.IB.A5SP.01A	-1.03412	-0.61731	0.279689	1.036993	1.78792	1.622304	1.203241	1.016223	-0.350680246	-0.630398874	0.5308053	1.063758527	-1.418642587	0.271306655	-1.772078983	-1.25383573	-0.135425213	0.562395146	1.607532801	-0.203239398	-0.341181156	0.642952632	0.942146051	-0.668775996	-1.21417406	-1.004170377	0.942538625	-0.158555
TCGA.IB.A5SQ.01A	0.697476	0.698644	-0.86041	-0.28925	-0.77411	-0.77499	-0.70694	-1.51281	-1.169944606	-1.07118498	-0.229302	0.719670118	0.147891125	0.941088752	1.009015631	1.461490068	0.747574516	-0.265836055	-0.670289351	0.071188942	-1.472039655	-0.447237507	-0.255579456	1.442173776	-0.207798038	1.70527591	-0.6063152	-0.48772
TCGA.IB.A6UF.01A	-1.36293	0.237102	-1.32186	1.286228	1.729609	0.734871	1.199903	-0.44272	-0.328185307	0.021796222	0.0896831	0.490797052	-1.000499741	1.093692059	-0.194988881	0.474255932	-0.151726494	-0.035711307	-0.050428625	-0.075417323	1.537976751	0.487633869	2	-0.82115894	-0.466487675	-1.828834285	0.343575816	-0.95558
TCGA.IB.AAUM.01A	1.250759	-0.67358	1.043191	-0.32145	0.125906	-0.32072	-2	-1.31122	0.731054349	0.317986531	1.1689037	-1.051686347	0.883629569	-0.258401817	0.598677468	0.065316188	-0.823982535	0.438743746	-1.092578876	-2	0.141747052	0.487809464	0.637389703	-0.488303016	0.141006691	1.445140552	0.448736808	-0.73525
TCGA.IB.AAUQ.01A	-0.54826	-0.20058	-1.43225	0.873586	1.445963	0.52681	0.211275	0.752469	0.543975825	-0.662591446	0.1492357	0.398716506	-0.615940167	0.446168522	0.252629668	0.442279909	-0.706892121	-0.252671592	-1.18280051	1.027081556	1.079276933	-0.273864512	0.608216068	-0.200310435	0.547347955	-0.901203455	-0.235207296	0.888247
TCGA.IB.AAUR.01A	1.910149	0.627017	1.314405	-1.78193	-1.48661	-0.59259	-0.512	-1.6877	0.390067075	1.398518493	0.2021805	-0.943643027	2	-2	0.272239776	0.658371731	1.071515814	-0.368267961	-0.48704143	-1.657415488	-0.346408529	-1.107206662	-0.819499948	0.214672164	-0.440433257	0.737003444	-1.390029218	-1.25527
TCGA.IB.AAUT.01A	0.669251	-0.14213	1.274859	-0.34551	-1.15893	-0.41072	-0.52943	-0.62562	0.716644307	1.029306537	-0.131468	-1.518710756	0.491360271	0.226198079	1.139912044	0.522802511	1.139736497	-1.107970997	-0.298531981	0.263791072	-0.299228719	-1.619746091	-0.684315599	0.674516615	2	-0.058679583	-0.499333752	-1.34477
TCGA.IB.AAUV.01A	1.077514	-0.19	0.943385	-1.62	-1.10386	-1.43	-0.97	-0.5586	0.902556295	0.861412736	-0.35177	-0.1675	0.885416386	-1.2292	0.664083349	1.689687644	1.406451546	-0.3467	-1.3857	0.408060022	-1.2405	-0.3048	-0.6542	0.968588468	0.576654477	1.165252321	-1.8671	-1.42

		411		724		579	737					4398		36528				54021	2135		47987	10293	94578				73257	985
TCGA.IB.AAUW.01A	1.5732	-0.83481	0.127241	-1.28469	-0.47221	-1.79894	-1.47033	0.007097	-0.183063781	0.834697629	0.7401226	-1.519763652	1.219803775	1.024744442	0.395900452	0.638067416	-0.076917338	0.284157549	-0.277101864	-0.894618996	-1.097558073	1.41315495	-0.158827243	-0.111356631	-0.538225937	0.162169743	0.029568392	0.16252536
TCGA.L1.A7W4.01A	-1.131039	0.860235	-0.84819	0.595253	1.132578	0.02585	-0.23502	0.560597	0.459853108	-1.487699226	0.953223	0.141591278	-0.326428685	0.245130529	-1.524774542	-1.289838682	-0.834469535	1.643323772	0.120665923	0.187101181	-0.321766705	0.37561064	2	-1.004328658	-1.074924884	0.712408239	1.043175469	1.285452
TCGA.LB.A8F3.01A	-0.80602	-0.46166	-0.70353	0.416621	1.444184	0.897052	0.62207	0.703517	-0.866957919	-1.523793277	0.9707189	0.918477189	-0.913360288	0.057431939	-0.966025799	0.17855691	-0.132805691	-1.118637028	2	-0.600077988	0.783497102	1.106837127	0.230521261	-1.711029175	0.305328066	0.345017528	0.817705674	0.498277
TCGA.LB.A9Q5.01A	-0.0462	-1.07325	-1.05881	1.637799	-0.8676	-0.43951	-2	-0.60712	-1.225434923	-0.556153573	1.3356644	-0.364360434	0.816396203	-0.156276058	1.094213378	1.028912537	1.153558447	-0.022657432	-1.132427479	-1.597987936	1.880045051	1.826766564	-0.03297971	0.69880766	1.197465963	0.235127315	-0.092784344	-0.96845
TCGA.RB.AA9M.01A	0.383675	0.646078	-0.31118	-0.43351	0.550786	-0.99484	0.453032	0.265773	0.912776756	-0.56730254	0.9331723	0.062423997	-1.083990512	1.218878647	-0.985975209	-0.787936929	-1.878137002	0.598567281	0.546265295	1.066168225	0.007760486	0.677665686	0.803006987	-1.431526136	-1.042065011	0.203384291	0.7299681	0.939176
TCGA.RL.AAAS.01A	1.577086	-1.08974	1.738357	-1.43771	-0.87219	-1.14051	-0.18764	-1.49269	0.200598548	1.354368367	-1.531696	0.433440236	1.356606362	-1.550398741	1.537908489	0.976808003	0.947210887	-2	0.34561676	-0.379625816	0.149729272	-2	-0.963955433	0.702273715	-0.904996051	-0.309404725	-0.501699666	-0.86423
TCGA.S4.A8RM.01A	-0.61675	0.425606	0.736409	-0.13624	0.8377	0.70414	0.523788	0.372967	0.456566053	0.861508013	1.1868333	-0.501238198	-0.228318392	-0.6259475	-1.60624555	-2	0.087918534	1.293465035	0.365038531	-0.8167044	0.438126606	0.039838968	0.336659117	-2	-1.245294569	0.086524053	1.378746285	0.898964
TCGA.S4.A8RO.01A	-1.46	2	-0.23	1.14395	0.251843	0.946943	-0.0071	0.808912	1.153093334	-0.4859	-0.970	1.149749542	-0.7250	0.923290214	0.442196745	0.275015576	-0.7422	0.010904527	0.026781405	-1.3081	1.583867759	-0.4562	0.082107806	0.02043881	-0.3431	-1.6158	-0.2004	0.689425

	696		222							93127	019		88851				86849			58618		62572			34542	5742	14208	
TCGA.US.A776.01A	-0.31997	0.271205	0.375722	-0.69958	1.302594	1.536994	0.994675	0.466744	0.707984969	-1.483120666	0.7617084	-0.132905962	-0.905564869	0.907263982	-0.140824057	-1.481587007	-0.77984344	1.462735396	1.079747947	-0.36859676	0.471501446	0.255499749	1.479338377	-1.98621384	-1.478672586	-2	1.22164044	1.304224
TCGA.US.A779.01A	-0.3532	-1.65028	-0.1989	0.877064	1.533363	1.248114	1.370542	1.126056	-0.253973953	-1.382835432	1.2236715	1.464778385	-0.903371641	0.98193254	-0.294842386	-1.376159705	-1.624124092	0.599266502	2	-0.93975418	0.140866135	-0.428774126	0.865718074	-1.757087706	-0.333591739	-0.67596346	1.910389553	-1.0116
TCGA.US.A77E.01A	-0.42734	-0.9477	-1.27669	0.950074	0.931466	1.842341	1.416871	0.903251	-0.010824104	-0.375654658	-0.353993	-0.733538477	-0.51666954	0.259057557	-0.401261636	-2	-1.189904389	0.663484451	0.903709148	1.777822149	1.20848157	0.39839327	0.667761903	-0.46878279	-0.895911846	-0.888305187	1.511070416	-0.569899
TCGA.US.A77G.01A	-0.82556	0.25228	-0.51379	0.231376	1.315909	0.366808	0.844951	-0.25537	0.593069426	0.174844244	0.0108902	-0.550101343	-0.709124905	0.716887076	-0.78956815	-1.465354811	-0.702844475	0.627885871	1.909127855	-0.184432804	-0.026882031	-0.196154838	0.934178403	-1.681760268	-1.291806361	0.29334285	2	1.162503
TCGA.US.A77J.01A	1.940219	0.375086	1.43059	-1.00565	-1.36516	-0.8299	-0.19326	-2	-0.480589486	1.498628003	0.3267032	-0.073548748	2	-2	0.629791093	0.190230578	1.211602861	-0.845685496	-0.089144937	-1.33714386	-0.367024691	-1.926087228	-1.071748464	0.818097097	-0.12551091	-0.247497422	-1.56355862	-1.2215
TCGA.XD.AAUG.01A	0.757483	-1.04114	-1.30838	-1.43463	-0.17183	-0.83664	-0.0361	-0.87297	1.127518737	0.034033243	1.6913568	-0.894134611	-0.208951369	0.309337728	0.641518713	1.288511526	-0.375633756	1.800738191	-1.377543162	1.169758157	-0.35205234	-1.117757729	0.193906295	0.728811315	1.316429092	1.08716854	0.017314385	-0.61233
TCGA.XD.AAUH.01A	1.690021	0.426121	0.889248	-2	-0.50957	-1.2039	-0.93454	-1.70579	0.790202957	0.421648501	2	-1.891549888	1.801073411	0.243336096	-0.181333283	1.161143825	0.437300689	0.660540019	-0.747823844	-0.620302225	-0.626670521	-1.323090873	-0.782253614	0.172947029	0.006017042	0.716486617	-1.268945306	-0.63294
TCGA.XD.AAUI.01A	1.464144	1.141085	0.21368	-0.23	0.256179	0.785362	-0.76	-1.68	-0.4795	-0.6845	-1.059	-0.0558	1.465471469	-1.1539	-0.0410	-0.1776	-0.1951	0.287930083	-0.1687	-1.2976	0.111633723	-0.6984	-0.7404	0.131080725	0.773274613	1.408343535	-0.3087	-1.87

				628			319	692	50248	13405	898	26982		95833	7088	2156	9494		91772	24812		37979	75635				14291	912
TCGA.XD.AAUL.01A	-1.58375	-1.11638	-1.63437	1.712864	-0.68142	0.800982	1.255473	0.960718	0.020554352	-1.400031374	-1.334748	0.644707075	-1.148191685	0.727491476	0.733279137	0.458055615	0.474852075	-0.312139183	1.723732776	2	0.615661634	1.493904857	0.516189884	1.058890683	-2	-0.875566521	0.295574635	-0.43951
TCGA.XN.A8T5.01A	1.573939	0.171775	1.464122	-2	-1.24002	-0.84618	-0.09403	-2	0.519454309	0.9850506	0.3636149	-0.914651364	1.74243574	-2	-1.112623338	1.116246683	0.786343068	-0.387077047	-0.593884983	0.11528792	-0.489143837	-0.720841888	-0.265310919	0.486909175	-0.74762463	2	-1.22816781	-0.4955
TCGA.Z5.AAPL.01A	2	2	1.594356	-1.1256	-1.59981	-0.44326	-0.43353	-2	0.611744579	1.578308586	0.2933633	-0.631024453	2	-2	-1.243830311	-0.74345216	0.729853373	0.706998258	0.017651408	-1.73309429	-0.515016425	-1.468690981	-1.565857782	0.357509528	-1.369613227	-0.289153021	-1.464998359	-0.2727

Supplementary Table 14: T cell differentiation score and subgroups by GSVA algorithm for TCGA_PAAD dataset.

TCGA_ID	Th1_diff_score	Th2_diff_score	Th17_diff_score	Th1/Th2_Polarization	Th17_group
TCGA.2J.AAB1.01A	0.263433726	-0.102952053	0.189186459	Th1_tend	Th17_diff_up
TCGA.2J.AAB4.01A	0.290160903	0.242038268	0.296473846	Th2_tend	Th17_diff_up
TCGA.2J.AAB6.01A	-0.291987279	-0.181454992	-0.199925191	Th2_tend	Th17_diff_down
TCGA.2J.AAB8.01A	-0.005277024	0.038204521	-0.064168178	Th2_tend	Th17_diff_down
TCGA.2J.AAB9.01A	0.13402391	0.157514239	0.326094723	Th2_tend	Th17_diff_up
TCGA.2J.AABA.01A	0.260106214	0.100167748	0.122774294	Th1_tend	Th17_diff_down
TCGA.2J.AABE.01A	-0.095170756	0.21371043	0.072064182	Th2_tend	Th17_diff_down
TCGA.2J.AABF.01A	0.471070531	0.300825917	0.507003102	Th1_tend	Th17_diff_up
TCGA.2J.AABH.01A	-0.196269778	-0.00221952	-0.199775059	Th2_tend	Th17_diff_down
TCGA.2J.AABI.01A	0.233208611	0.256839581	0.195206482	Th2_tend	Th17_diff_up
TCGA.2J.AABK.01A	-0.10371624	-0.265597406	-0.161592433	Th1_tend	Th17_diff_down
TCGA.2J.AABO.01A	-0.005825697	0.094153015	-0.113649685	Th2_tend	Th17_diff_down
TCGA.2J.AABP.01A	0.344601414	0.109969752	0.327453783	Th1_tend	Th17_diff_up
TCGA.2J.AABR.01A	0.466975217	0.251640129	0.588886902	Th1_tend	Th17_diff_up
TCGA.2J.AABT.01A	0.137933799	0.272579769	0.313447292	Th2_tend	Th17_diff_up
TCGA.2J.AABU.01A	0.079882024	0.247865376	-0.009012907	Th2_tend	Th17_diff_down
TCGA.2J.AABV.01A	-0.279686534	-0.465893395	-0.352436111	Th1_tend	Th17_diff_down
TCGA.2L.AAQA.01A	-0.096752077	-0.034181658	-0.147471247	Th2_tend	Th17_diff_down
TCGA.2L.AAQE.01A	0.06685361	0.18665606	0.1148447	Th2_tend	Th17_diff_down
TCGA.2L.AAQI.01A	-0.027692976	0.015701792	-0.000849551	Th2_tend	Th17_diff_down
TCGA.2L.AAQJ.01A	0.058129267	0.136095817	-0.015957143	Th2_tend	Th17_diff_down
TCGA.2L.AAQL.01A	-0.012010815	-0.100374193	-0.063667912	Th2_tend	Th17_diff_down
TCGA.2L.AAQM.01A	-0.183485285	0.057509354	-0.186563167	Th2_tend	Th17_diff_down
TCGA.3A.A9I5.01A	-0.18677469	0.212693578	-0.078358734	Th2_tend	Th17_diff_down
TCGA.3A.A9I7.01A	0.265634928	0.195951779	0.177324366	Th2_tend	Th17_diff_up
TCGA.3A.A9I9.01A	-0.041756903	-0.341081685	-0.314613047	Th1_tend	Th17_diff_down
TCGA.3A.A9IB.01A	0.021276394	0.229467018	0.077113203	Th2_tend	Th17_diff_down
TCGA.3A.A9IC.01A	-0.127337443	0.172401328	-0.113082074	Th2_tend	Th17_diff_down
TCGA.3A.A9IH.01A	-0.04304325	-0.156238347	-0.172667803	Th1_tend	Th17_diff_down
TCGA.3A.A9IJ.01A	-0.202168703	-0.411690899	-0.280049322	Th1_tend	Th17_diff_down
TCGA.3A.A9IL.01A	-0.159067447	-0.26425212	-0.21851114	Th1_tend	Th17_diff_down

TCGA.3A.A9IN.01A	-0.011820429	-0.236431602	-0.043159885	Th1_tend	Th17_diff_down
TCGA.3A.A9IO.01A	-0.057171957	-0.332679211	-0.218909437	Th1_tend	Th17_diff_down
TCGA.3A.A9IR.01A	-0.218094787	-0.198301821	-0.322062831	Th2_tend	Th17_diff_down
TCGA.3A.A9IS.01A	-0.163833866	-0.295729602	-0.151179786	Th1_tend	Th17_diff_down
TCGA.3A.A9IU.01A	-0.310722471	0.270389237	0.008994837	Th2_tend	Th17_diff_down
TCGA.3A.A9IV.01A	-0.072081413	-0.11964211	-0.09573282	Th2_tend	Th17_diff_down
TCGA.3A.A9IX.01A	0.431499693	-0.080203027	0.552262314	Th1_tend	Th17_diff_up
TCGA.3A.A9IZ.01A	0.066289694	0.121366988	-0.006166682	Th2_tend	Th17_diff_down
TCGA.3A.A9J0.01A	-0.397703031	0.094582586	-0.215511377	Th2_tend	Th17_diff_down
TCGA.3E.AAAY.01A	0.406240095	0.082149522	0.436336072	Th1_tend	Th17_diff_up
TCGA.3E.AAAZ.01A	-0.280551317	0.083404331	-0.168616931	Th2_tend	Th17_diff_down
TCGA.F2.6879.01A	-0.070804064	0.11081452	0.00656046	Th2_tend	Th17_diff_down
TCGA.F2.6880.01A	-0.213910909	-0.466264042	-0.36163173	Th1_tend	Th17_diff_down
TCGA.F2.7273.01A	0.33513028	0.121276682	0.550744893	Th1_tend	Th17_diff_up
TCGA.F2.7276.01A	0.364500642	0.2348899	0.58836803	Th1_tend	Th17_diff_up
TCGA.F2.A44G.01A	-0.185006284	0.277024336	-0.012762821	Th2_tend	Th17_diff_down
TCGA.F2.A44H.01A	-0.127214449	-0.172360222	0.119510576	Th2_tend	Th17_diff_down
TCGA.F2.A7TX.01A	0.417828443	0.426582003	0.372572733	Th2_tend	Th17_diff_up
TCGA.F2.A8YN.01A	-0.266202979	0.104649913	-0.281295991	Th2_tend	Th17_diff_down
TCGA.FB.A4P5.01A	0.45782466	0.312153445	0.499554978	Th1_tend	Th17_diff_up
TCGA.FB.A4P6.01A	0.368390772	0.341952526	0.419579536	Th2_tend	Th17_diff_up
TCGA.FB.A545.01A	-0.187953686	0.209230877	-0.129448901	Th2_tend	Th17_diff_down
TCGA.FB.A5VM.01A	0.201655134	-0.115866792	0.085481105	Th1_tend	Th17_diff_down
TCGA.FB.A78T.01A	-0.199622413	0.152730532	0.103176368	Th2_tend	Th17_diff_down
TCGA.FB.A7DR.01A	0.139752291	0.348061556	0.147498127	Th2_tend	Th17_diff_up
TCGA.FB.AAPP.01A	-0.41523886	-0.094238543	-0.274609685	Th2_tend	Th17_diff_down
TCGA.FB.AAPQ.01A	-0.159441182	0.12131319	-0.077736501	Th2_tend	Th17_diff_down
TCGA.FB.AAPS.01A	0.303188443	0.173736563	0.335304371	Th1_tend	Th17_diff_up
TCGA.FB.AAPU.01A	-0.087594101	0.191512225	-0.162005	Th2_tend	Th17_diff_down
TCGA.FB.AAPY.01A	0.29189901	0.096057096	0.266917052	Th1_tend	Th17_diff_up
TCGA.FB.AAPZ.01A	0.346341542	0.424913102	0.347735312	Th2_tend	Th17_diff_up
TCGA.FB.AAQ0.01A	-0.199024976	0.189362976	-0.178141437	Th2_tend	Th17_diff_down
TCGA.FB.AAQ1.01A	-0.227186612	-0.204161987	-0.069449088	Th2_tend	Th17_diff_down

TCGA.FB.AAQ2.01A	-0.251152395	-0.359000637	-0.268574346	Th1_tend	Th17_diff_down
TCGA.FB.AAQ3.01A	-0.129133387	-0.350173271	-0.186233459	Th1_tend	Th17_diff_down
TCGA.FB.AAQ6.01A	-0.206919379	0.085408792	-0.299969935	Th2_tend	Th17_diff_down
TCGA.H6.8124.01A	-0.319029626	0.150188788	-0.090242519	Th2_tend	Th17_diff_down
TCGA.H6.A45N.01A	0.357252929	0.178964753	0.477933387	Th1_tend	Th17_diff_up
TCGA.H8.A6C1.01A	0.159125268	0.350589695	0.12311845	Th2_tend	Th17_diff_down
TCGA.HV.A5A3.01A	-0.137827479	0.373125789	-0.033989773	Th2_tend	Th17_diff_down
TCGA.HV.A5A4.01A	-0.065410029	0.275812335	0.092622245	Th2_tend	Th17_diff_down
TCGA.HV.A5A5.01A	0.33603099	0.226033491	0.424831886	Th1_tend	Th17_diff_up
TCGA.HV.A5A6.01A	-0.048450373	0.142453794	-0.159247345	Th2_tend	Th17_diff_down
TCGA.HV.A7OL.01A	0.294459259	0.100966986	0.130907242	Th1_tend	Th17_diff_down
TCGA.HV.A7OP.01A	-0.220573213	-0.359364424	-0.307935045	Th1_tend	Th17_diff_down
TCGA.HV.AA8V.01A	-0.250876951	0.166902553	0.099441144	Th2_tend	Th17_diff_down
TCGA.HV.AA8X.01A	-0.120703827	0.018893427	-0.227979902	Th2_tend	Th17_diff_down
TCGA.HZ.7289.01A	-0.162846177	-0.123678464	-0.188013008	Th2_tend	Th17_diff_down
TCGA.HZ.7918.01A	0.465597545	0.223997891	0.638173869	Th1_tend	Th17_diff_up
TCGA.HZ.7919.01A	0.308106907	0.145933037	0.398042443	Th1_tend	Th17_diff_up
TCGA.HZ.7920.01A	0.352262598	0.165607046	0.430405195	Th1_tend	Th17_diff_up
TCGA.HZ.7922.01A	0.119837974	0.267168864	0.37009648	Th2_tend	Th17_diff_up
TCGA.HZ.7923.01A	0.520014469	0.21632898	0.58803644	Th1_tend	Th17_diff_up
TCGA.HZ.7924.01A	0.286816112	0.113253485	0.247642496	Th1_tend	Th17_diff_up
TCGA.HZ.7925.01A	0.05868002	0.114951881	0.248791028	Th2_tend	Th17_diff_up
TCGA.HZ.7926.01A	0.337512707	0.365547061	0.357495463	Th2_tend	Th17_diff_up
TCGA.HZ.8001.01A	0.337712351	0.116044978	0.347830951	Th1_tend	Th17_diff_up
TCGA.HZ.8002.01A	0.369094696	0.213779962	0.54535144	Th1_tend	Th17_diff_up
TCGA.HZ.8003.01A	0.412117195	-0.187767523	0.414088499	Th1_tend	Th17_diff_up
TCGA.HZ.8005.01A	-0.016594925	-0.051104021	-0.012177779	Th2_tend	Th17_diff_down
TCGA.HZ.8315.01A	0.373061768	0.19517392	0.505265454	Th1_tend	Th17_diff_up
TCGA.HZ.8317.01A	-0.183204176	-0.317188482	0.012544982	Th1_tend	Th17_diff_down
TCGA.HZ.8519.01A	0.384729771	0.05784918	0.356995498	Th1_tend	Th17_diff_up
TCGA.HZ.8636.01A	0.252777315	0.236280325	0.403781078	Th2_tend	Th17_diff_up
TCGA.HZ.8637.01A	0.52256027	0.1701095	0.577066793	Th1_tend	Th17_diff_up
TCGA.HZ.8638.01A	0.30189148	0.038652626	0.235279856	Th1_tend	Th17_diff_up

TCGA.HZ.A49G.01A	0.38797464	0.199756046	0.544662839	Th1_tend	Th17_diff_up
TCGA.HZ.A49H.01A	0.426762203	-0.181702225	0.369868362	Th1_tend	Th17_diff_up
TCGA.HZ.A49I.01A	0.179595319	0.118970886	0.357212638	Th2_tend	Th17_diff_up
TCGA.HZ.A4BH.01A	0.412406766	0.278027147	0.478087215	Th1_tend	Th17_diff_up
TCGA.HZ.A4BK.01A	0.379313638	0.327774691	0.365228081	Th2_tend	Th17_diff_up
TCGA.HZ.A77O.01A	-0.21840431	-0.135839163	-0.194724688	Th2_tend	Th17_diff_down
TCGA.HZ.A77P.01A	-0.002358725	0.218626146	0.167341593	Th2_tend	Th17_diff_up
TCGA.HZ.A77Q.01A	0.459545255	0.374311191	0.586140209	Th2_tend	Th17_diff_up
TCGA.HZ.A8P0.01A	-0.314194704	-0.064270748	-0.278713262	Th2_tend	Th17_diff_down
TCGA.HZ.A8P1.01A	-0.01548523	-0.006950529	-0.329866855	Th2_tend	Th17_diff_down
TCGA.HZ.A9TJ.01A	-0.224354307	-0.063342849	-0.259655828	Th2_tend	Th17_diff_down
TCGA.IB.7644.01A	0.182731292	0.158556145	0.145111084	Th2_tend	Th17_diff_up
TCGA.IB.7645.01A	0.395850004	0.242386191	0.63836827	Th1_tend	Th17_diff_up
TCGA.IB.7646.01A	0.160184665	0.17470001	0.343133933	Th2_tend	Th17_diff_up
TCGA.IB.7649.01A	0.354694221	0.018598234	0.505255356	Th1_tend	Th17_diff_up
TCGA.IB.7651.01A	0.470083136	0.245612633	0.50015636	Th1_tend	Th17_diff_up
TCGA.IB.7652.01A	0.041288533	0.011450592	0.365343888	Th2_tend	Th17_diff_up
TCGA.IB.7654.01A	-0.181584405	0.024878192	0.203692453	Th2_tend	Th17_diff_up
TCGA.IB.7885.01A	0.286667677	0.441773009	0.489098193	Th2_tend	Th17_diff_up
TCGA.IB.7886.01A	0.33610948	0.20646703	0.306842468	Th1_tend	Th17_diff_up
TCGA.IB.7887.01A	0.165860889	0.263256915	0.217845879	Th2_tend	Th17_diff_up
TCGA.IB.7888.01A	0.42116113	0.160845788	0.610433485	Th1_tend	Th17_diff_up
TCGA.IB.7889.01A	0.316859234	-0.074331363	0.363720038	Th1_tend	Th17_diff_up
TCGA.IB.7890.01A	-0.120788191	0.131675622	0.244576526	Th2_tend	Th17_diff_up
TCGA.IB.7891.01A	0.436463306	0.045097068	0.565425724	Th1_tend	Th17_diff_up
TCGA.IB.7893.01A	-0.062285563	0.194789684	0.283776271	Th2_tend	Th17_diff_up
TCGA.IB.7897.01A	0.447150136	0.157650312	0.600006311	Th1_tend	Th17_diff_up
TCGA.IB.8126.01A	0.261307685	-0.288912642	0.239898537	Th1_tend	Th17_diff_up
TCGA.IB.8127.01A	0.340162431	0.25583119	0.428119341	Th2_tend	Th17_diff_up
TCGA.IB.A5SO.01A	0.326516506	0.123963165	0.437731255	Th1_tend	Th17_diff_up
TCGA.IB.A5SP.01A	-0.180048971	-0.0442385	-0.348489533	Th2_tend	Th17_diff_down
TCGA.IB.A5SQ.01A	0.122031188	0.300948374	0.333792785	Th2_tend	Th17_diff_up
TCGA.IB.A5SS.01A	-0.146662573	0.143350057	0.138983778	Th2_tend	Th17_diff_down

TCGA.IB.A5ST.01A	0.462096526	0.284823633	0.496121764	Th1_tend	Th17_diff_up
TCGA.IB.A6UF.01A	-0.220826475	0.190763967	-0.244498027	Th2_tend	Th17_diff_down
TCGA.IB.A6UG.01A	-0.010568543	0.107738264	0.054519927	Th2_tend	Th17_diff_down
TCGA.IB.A7LX.01A	-0.030004381	0.09820458	-0.111841431	Th2_tend	Th17_diff_down
TCGA.IB.A7M4.01A	0.138172407	0.275374091	-0.004874678	Th2_tend	Th17_diff_down
TCGA.IB.AAUM.01A	0.194354347	-0.444412429	-0.019503648	Th1_tend	Th17_diff_down
TCGA.IB.AAUN.01A	0.009104842	-0.414608306	-0.011472546	Th1_tend	Th17_diff_down
TCGA.IB.AAUO.01A	0.059686462	0.041647603	0.002964518	Th2_tend	Th17_diff_down
TCGA.IB.AAUP.01A	0.429022564	0.376147513	0.585296447	Th2_tend	Th17_diff_up
TCGA.IB.AAUQ.01A	-0.140935052	-0.316220016	-0.054436909	Th1_tend	Th17_diff_down
TCGA.IB.AAUR.01A	0.440022869	0.44442979	0.578179007	Th2_tend	Th17_diff_up
TCGA.IB.AAUS.01A	0.351031929	0.257466012	0.543247169	Th1_tend	Th17_diff_up
TCGA.IB.AAUT.01A	0.384825532	0.200726203	0.550610002	Th1_tend	Th17_diff_up
TCGA.IB.AAUU.01A	0.027311725	0.314398096	0.143425504	Th2_tend	Th17_diff_up
TCGA.IB.AAUV.01A	0.329135613	0.144147983	0.456418331	Th1_tend	Th17_diff_up
TCGA.IB.AAUW.01A	0.378980826	0.145350484	0.498051199	Th1_tend	Th17_diff_up
TCGA.L1.A7W4.01A	-0.091037573	0.151673845	-0.24363631	Th2_tend	Th17_diff_down
TCGA.LB.A7SX.01A	0.026313514	0.108590842	-0.104854031	Th2_tend	Th17_diff_down
TCGA.LB.A8F3.01A	-0.186667345	-0.162899476	-0.278774624	Th2_tend	Th17_diff_down
TCGA.LB.A9Q5.01A	-0.120075813	-0.47743973	-0.019639572	Th1_tend	Th17_diff_down
TCGA.M8.A5N4.01A	0.130373764	0.323046765	0.216114735	Th2_tend	Th17_diff_up
TCGA.OE.A75W.01A	0.125367534	0.037599059	-0.040244068	Th2_tend	Th17_diff_down
TCGA.PZ.A5RE.01A	0.127207094	0.15713476	0.092956986	Th2_tend	Th17_diff_down
TCGA.Q3.A5QY.01A	0.475292731	0.336088613	0.508887311	Th1_tend	Th17_diff_up
TCGA.Q3.AA2A.01A	-0.037813255	0.156630994	-0.244889023	Th2_tend	Th17_diff_down
TCGA.RB.A7B8.01A	0.223061354	-0.06266711	0.082428991	Th1_tend	Th17_diff_down
TCGA.RB.AA9M.01A	0.063442415	0.14846587	0.161066356	Th2_tend	Th17_diff_up
TCGA.RL.AAAS.01A	0.419165106	-0.070872227	0.376218413	Th1_tend	Th17_diff_up
TCGA.S4.A8RM.01A	0.213178314	0.097378574	0.086471857	Th1_tend	Th17_diff_down
TCGA.S4.A8RO.01A	-0.175118405	0.203772183	-0.149183443	Th2_tend	Th17_diff_down
TCGA.S4.A8RP.01A	0.211433834	0.167559123	0.185158582	Th2_tend	Th17_diff_up
TCGA.US.A774.01A	0.395442632	0.352606117	0.463992699	Th2_tend	Th17_diff_up
TCGA.US.A776.01A	-0.13376041	-0.01797	-0.250992654	Th2_tend	Th17_diff_down

TCGA.US.A779.01A	-0.193815056	-0.049442215	-0.260195965	Th2_tend	Th17_diff_down
TCGA.US.A77E.01A	-0.202146319	0.097223292	-0.107566439	Th2_tend	Th17_diff_down
TCGA.US.A77G.01A	-0.103221603	0.040924344	-0.1082911	Th2_tend	Th17_diff_down
TCGA.US.A77J.01A	0.400658668	0.273639646	0.444142392	Th1_tend	Th17_diff_up
TCGA.XD.AAUG.01A	0.072396361	-0.243377317	0.228687628	Th1_tend	Th17_diff_up
TCGA.XD.AAUH.01A	0.321407506	0.216811279	0.439042505	Th1_tend	Th17_diff_up
TCGA.XD.AAUI.01A	0.178886548	0.241253483	0.320182393	Th2_tend	Th17_diff_up
TCGA.XD.AAUL.01A	-0.048769006	0.331343349	0.0861283	Th2_tend	Th17_diff_down
TCGA.XN.A8T3.01A	0.215716877	0.115948828	0.115090537	Th1_tend	Th17_diff_down
TCGA.XN.A8T5.01A	0.467431951	0.287619675	0.456428176	Th1_tend	Th17_diff_up
TCGA.YB.A89D.01A	0.090519369	0.153992039	0.337681108	Th2_tend	Th17_diff_up
TCGA.YH.A8SY.01A	-0.260820329	0.186784488	-0.10647078	Th2_tend	Th17_diff_down
TCGA.YY.A8LH.01A	-0.209916045	0.212714436	-0.11927367	Th2_tend	Th17_diff_down
TCGA.Z5.AAPL.01A	0.480322803	0.383224232	0.559732082	Th1_tend	Th17_diff_up
